# A supramolecular metalloenzyme possessing robust oxidase-mimetic catalytic function

**DOI:** 10.1038/s41467-023-39779-6

**Published:** 2023-07-07

**Authors:** Shichao Xu, Haifeng Wu, Siyuan Liu, Peidong Du, Hui Wang, Haijun Yang, Wenjie Xu, Shuangming Chen, Li Song, Jikun Li, Xinghua Shi, Zhen-Gang Wang

**Affiliations:** 1 State Key Laboratory of Organic-Inorganic Composites, Key Lab of Biomedical Materials of Natural Macromolecules (Beijing University of Chemical Technology, Ministry of Education), Beijing Laboratory of Biomedical Materials, College of Materials Science and Engineering, Beijing University of Chemical Technology, Beijing, 100029 China; 2 Laboratory of Theoretical and Computational Nanoscience, CAS Center for Excellence in Nanoscience, National Center for Nanoscience and Technology, Beijing, 100190 China; 3 Department of Chemistry, Tsinghua University, Beijing, 10084 China; 4 National Synchrotron Radiation Laboratory, CAS Center for Excellence in Nanoscience, University of Science and Technology of China, Hefei, 230029 China; 5 Institute of Chemistry, Chinese Academy of Sciences (ICCAS), Beijing, 100190 China

**Keywords:** Self-assembly, Catalysis

## Abstract

Enzymes fold into unique three-dimensional structures to distribute their reactive amino acid residues, but environmental changes can disrupt their essential folding and lead to irreversible activity loss. The de novo synthesis of enzyme-like active sites is challenging due to the difficulty of replicating the spatial arrangement of functional groups. Here, we present a supramolecular mimetic enzyme formed by self-assembling nucleotides with fluorenylmethyloxycarbonyl (Fmoc)-modified amino acids and copper. This catalyst exhibits catalytic functions akin those of copper cluster-dependent oxidases, and catalytic performance surpasses to date-reported artificial complexes. Our experimental and theoretical results reveal the crucial role of periodic arrangement of amino acid components, enabled by fluorenyl stacking, in forming oxidase-mimetic copper clusters. Nucleotides provide coordination atoms that enhance copper activity by facilitating the formation of a copper-peroxide intermediate. The catalyst shows thermophilic behavior, remaining active up to 95 °C in an aqueous environment. These findings may aid the design of advanced biomimetic catalysts and offer insights into primordial redox enzymes.

## Abstract (web summary)

The de novo synthesis of enzyme-like active sites remains a challenge, due to the difficulties in imitating the spatial arrangement of the functional groups. Here, the authors report the construction of a supramolecular mimetic enzyme possessing catechol oxidase-like copper-cluster active sites.

## Introduction

Metalloenzymes catalyze reactions through the cooperation between exquisitely-arranged amino acid residues and metal-based cofactors. Protein structure is susceptible to environmental changes, which often cause non-recoverable loss of enzyme activity. Burgeoning research into enzyme-inspired supramolecular self-assembly and catalysis, which entails creating enzyme-like active sites, has achieved chemical reactions with substrates similar to those catalyzed by enzymes^1–4^. Despite some advancements toward synthetic enzyme mimics of proteins^5–12^, the de novo creation of enzyme-like metallocluster active sites, which have a strict requirement for the geometric distribution of ligand groups and are capable of catalyzing some of the most remarkable reactions in nature, remains largely unresolved.

Metallocluster-dependent enzymes are capable of catalyzing the most basic oxidation-reduction transformations, which generate both chemical building blocks and energy to fuel metabolic processes^13^. In these enzymes, such as nitrogenases, hydrogenases, methane monooxygenases and catechol oxidases, electronic interactions between metals allow the metallocenters to bind the substrates and shuttle electrons into and out of the active site^13^. In the example of catechol oxidases (a family of copper proteins), three types of Cu (Type 1 Cu [T1Cu], Type 2 Cu [T2Cu] and Type 3 Cu [T3Cu]), with distinct coordination spheres, have been identified in the active sites^14–16^. T2Cu is coordinated in either a square-planar or distorted tetrahedral geometry by two nitrogen atoms from a histidine residue, with the remaining site ligated by water. T3Cu contains a dicopper core, in which each copper atom is coordinated by three histidine residues and bridged by a hydroxyl ion. T2Cu and T3Cu are organized into a trinuclear copper cluster (TNC)^14,17^. Moreover, there is coordination unsaturation for the copper cluster of the natural enzymes, despite the abundance of ligands (e.g., the side chain imidazole and the backbone carbonyl groups) available from the protein chains. These structural characteristics facilitate oxygen binding and activation at the copper site, promoting the catalytic oxidation. Several groups have attempted to create catechol oxidase-mimicking catalysts^18–22^. However, the structural complexity of the coordination spheres of the synthetic copper complexes was far from those of natural copper centers, leading to considerably lower activity than natural enzymes (e.g., laccase).

Native enzymes rely on well-defined tertiary structures, which are formed through noncovalent interactions in the protein chain, to organize essential functional groups in a pocket where the active sites are created. We were inspired to arrange ligands in an ordered manner, via designed molecular self-assembly (or folding), for clustering metals. The noncovalent interactions among the molecular building block may allow the self-assemblies to have a great structural flexibility, similar to native enzymes, which facilitates the access of the molecular substrates to the active sites inside the supramolecular entity. This approach presents the challenge of producing coordination spheres through the noncovalent association of small molecules. Successful generation of such self-assembled functional groups may shed light on the poorly understood mechanisms by which the mixture of simple molecules and metals evolved into metallocluster-dependent enzymes.

**Fig. 1:**
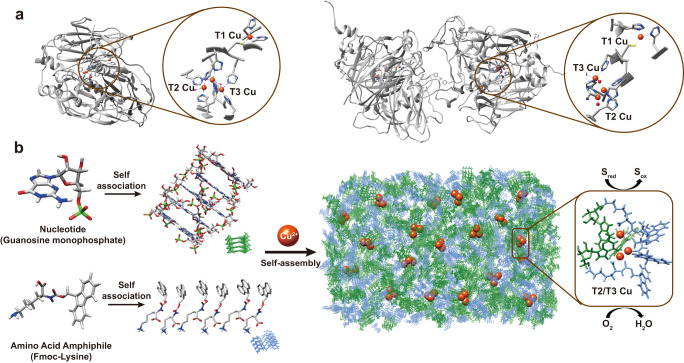
Design of copper-dependent supramolecular catalysts inspired by natural catechol oxidases. **a** Structures of *Trametes versicolor* laccase (PDB: 1GYC) and ascorbic acid oxidase (PDB: 1AOZ), showing the copper cluster and distributed residues in the active sites. **b** Schematic self-assembly of nanoaggregates comprising nucleotides (e.g., guanosine monophosphate, GMP), amphiphilic amino acids (e.g., Fmoc-Lysine-OH, Fmoc-K) and Cu^2+^ ions, that form oxidase-mimetic copper cluster active sites and catalytic activities. Atom colors in the chemical structures: Nitrogen, blue; oxygen, red; phosphorous, green; carbon, dim gray; hydrogen, light gray.

Here, we describe the self-assembly of Cu^2+^ with nucleotides and fluorenylmethyloxycarbonyl (Fmoc)-modified amino acid components to construct catechol oxidase-like copper-cluster active sites (Fig. 1). The fluorenyl moiety with an ortho-fused tricyclic structure may stack in a directional manner, which allows for the ordered arrangement of ligand groups from the side chain or backbones of the amino acids, for creating coordinatively unsaturated copper centers. Nucleotides can provide nucleobase or phosphate ligands^23,24^. It is expected that these two components can self-assemble to create coordination spheres of Cu^2+^ with a chemical environment similar to the enzymatic copper site. Here, we demonstrate the generation of hybrid self-assemblies that exhibit synergistic catechol oxidase-mimicking activities that were significantly greater than previously reported synthetic catechol oxidase mimics. Moreover, the catalysts showed thermophilic activities, suggesting a robust catalytic effect.

## Results

### Structures of the assemblies

**Fig. 2:**
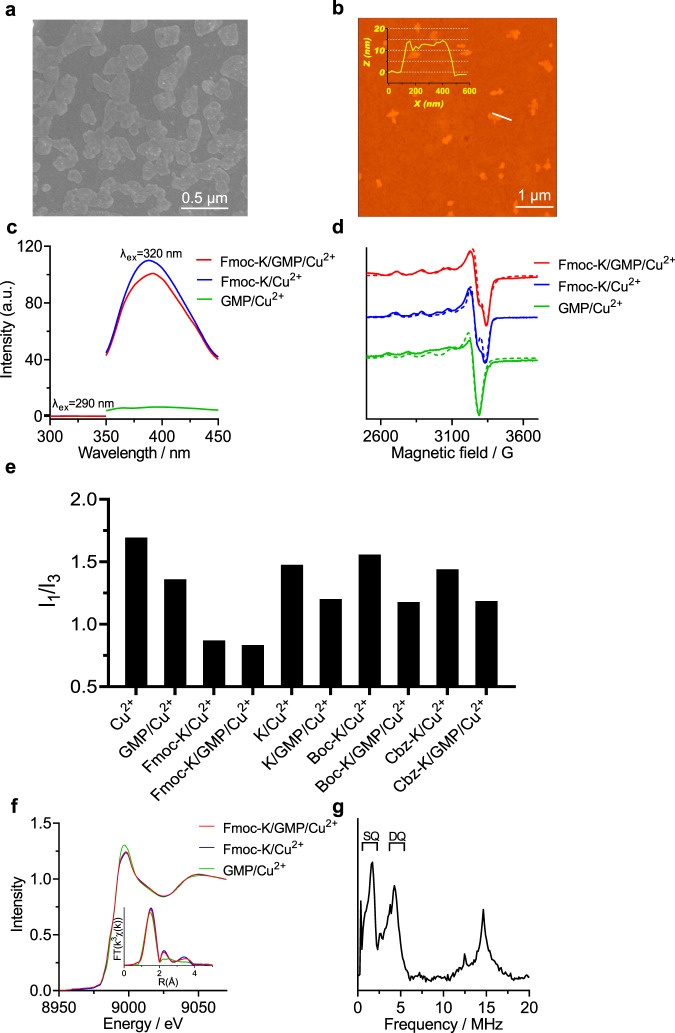
Structural analysis of the Cu^2+^-containing complexes. **a** SEM and **b** AFM images of the self-assembled Fmoc-K/GMP/Cu^2+^ complex. Inset is the cross-section analysis of the sample. [GMP]: 10 mM. [Fmoc-K]: 5 mM. [Cu^2+^]: 5 μM. *n* = 3 independent experiments. **c** Fluorescence emission spectra of the GMP/Cu^2+^, Fmoc-K/Cu^2+^ and Fmoc-K/GMP/Cu^2+^ complexes. [GMP]: 10 mM. [Fmoc-K]: 5 mM. [Cu^2+^] = 5 μM. **d** Experimental (solid) and simulated (dotted) EPR spectra of Fmoc-K/GMP/Cu^2+^, Fmoc-K/Cu^2+^ and GMP/Cu^2+^. [GMP]: 10 mM. [Fmoc-K]: 5 mM. [Cu^2+^] = 100 μM. **e** I_1_/I_3_ values of the mixtures of pyrene and Cu^2+^-containing complexes. [Pyrene] = 5 μM, [Cu^2+^] = 5 μM. [GMP] = 10 mM, [Fmoc-K] = 5 mM, [K] = 5 mM, [Boc-K] = 5 mM, [Cbz-K] = 5 mM. **f** K-edge X-ray absorption spectra and Fourier transforms of the k^3^-weighted EXAFS modulations for Fmoc-K/GMP/Cu^2+^ (red), Fmoc-K/Cu^2+^ (blue) and GMP/Cu^2+^(green). [GMP]: 10 mM. [Fmoc-K]: 5 mM. [Cu^2+^] = 200 μM. **g** Three-pulse ESEEM spectrum at 3436 G of Fmoc-K/GMP/Cu^2+^. τ = 200 ns. *k* = 1.81, *η* = 0.43. [Cu^2+^] = 250 μM. [Fmoc-K] = 5 mM, [GMP] = 10 mM.

We first used guanosine monophosphate (GMP) and Nα-9-fluorenylmethoxycarbonyl-lysine (Fmoc-K; Supplementary Fig. 1) to self-assemble with Cu^2+^. SEM, AFM and TEM imaging revealed that GMP/Cu^2+^ or Fmoc-K/Cu^2+^ self-assembled into nanoscale fibers, while self-assembly of GMP with Fmoc-K and Cu^2+^ produced irregular nanosheets with lateral dimensions of less than 500 nm and heights of less than 20 nm (Fig. 2a, b, Supplementary Fig. 2 and Supplementary Fig. 3). The circular dichroism (CD) spectrum of GMP shows positive bands at 280 nm, a negative band at ca. 250 nm and a positive long-wavelength tail at 304 nm. The bisignate CD signal is characteristic of a G-quartet maintained by π stacking and hydrogen bonding (Supplementary Fig. 4)^25–27^. The CD spectrum of Fmoc-K/Cu^2+^ fibers displays a negative peak at ca. 212 nm and an absorbance between 240 nm and 320 nm, which can be ascribed to backbone hydrogen bonding (e.g., a mixed β-sheet and disordered structure) and transitions from fluorenyl−fluorenyl interactions. The CD spectra of the self-assembled Fmoc-K/GMP/Cu^2+^ at different Cu^2+^ concentrations exhibit the spectral features of GMP/Cu^2+^ and Fmoc-K/Cu^2+^, but not simply an overlap of their respective spectra (Supplementary Fig. 4). Fmoc-K/Cu^2+^ exhibits a fluorescence emission at 384 nm, which is characteristic of a parallel arrangement of fluorenyl rings^28–31^, along with a very slight emission at 315 nm, which is characteristic of free Fmoc groups without self-assembly (Fig. 2c)^28,29^. No fluorescence emission of GMP was found. A small decrease in the emission intensity of Fmoc-K/Cu^2+^ was observed after assembly with GMP, indicating that GMP slightly interfered with the aggregation of Fmoc-K. These results suggest the successful assembly of GMP, Fmoc-K and Cu^2+^, retaining the self-associating structures of GMP and Fmoc-K.

We evaluated the ground-state electronic states of the Cu^2+^ by low-temperature X-band continuous-wave electron paramagnetic resonance (EPR; Fig. 2d and Supplementary Fig. 5). All the simulated EPR spectra were generated using MATLAB toolbox EasySpin to obtain the g (g_//_ and g _┴_) and A values^32^, which reflect the coordination environments and the unpaired copper electrons in the *d*_x_^2^_–y_^2^ orbital pointing directly at the ligands^33–35^. In particular, the g-shift ∆g (≈ g - g_e_) (g_e_, free electron g factor, ~2.0023) is frequently used to indicate the strength of the crystal field. As shown in Supplementary Table 1, 2 and 3, the complexes show similar ∆g_┴_ (≈g _┴_ - g_e_). The Fmoc-K/Cu^2+^ complex exhibited two sets of characteristic EPR parameters (g_//_, 2.300, A_//_, 168 × 10^-4^ cm^-1^; g_//_, 2.250, A_//_, 187 × 10^-4^ cm^-1^), attributed to two Cu (II) species with distinct coordination environments. The EPR spectra of Fmoc-K/GMP/Cu^2+^ show only a single EPR hyperfine feature (g_//_, 2.269, A_//_, 175 × 10^-4^ cm^-1^), the parameters of which were different from those of the Fmoc-K/Cu^2+^ and GMP/Cu^2+^ complexes (g_//_, 2.350, A_//_, 158 × 10^-4^ cm^-1^), indicating a significant change in the ligand environments of Cu^2+^, upon Fmoc-K and GMP self-assembly. This result may also reflect the coordination of Cu^2+^ to both Fmoc-K and GMP components. The g_//_ and A_//_ values of Fmoc-K/Cu^2+^ and GMP/Fmoc-K/Cu^2+^ are consistent with the formation of a paramagnetic T2-Cu site (g_//_, 2.22~2.30, A_//_,158~200 × 10^-4^ cm^-1^)^15,16,36,37^. To further understand the structure of the Cu^2+^ site, we obtained XAS (X-ray absorption spectroscopy) spectra of these copper complexes at the Cu-K edge (XAFS), which exhibited a intense transition at ~8986 eV and a very weak 1s → 3d pre-edge transition at ~8979 eV, indicating the oxidation state of copper in these complexes^38^. Very similar 1s → 3d transition peak between Fmoc-K/Cu^2+^ and Fmoc-K/GMP/Cu^2+^ complexes indicate they have similar coordination structures of copper^39^. The Fourier transform (FT) data of extended X-ray absorption fine structure (EXAFS) (Fig. 2f) show two Cu-Cu distances in both Fmoc-K/Cu^2+^ (~2.59 Å, ~3.75 Å) and GMP/Fmoc-K/Cu^2+^ (~2.59 Å, ~3.84 Å), the longer distances are similar to the dicopper of T3Cu (~3.43 Å). There were no signals corresponding to the Cu-Cu observed in GMP/Cu^2+^, and its g and A values were closer to those of CuSO_4_ in water (g_//_, 2.389, A_//_ 134 × 10^-4^ cm^-1^). These spectral results indicate that there were both T2-mimetic single copper (paramagnetic) and T3Cu-mimetic dicopper sites in the Fmoc-K-based self-assemblies.

We further investigated the formation of Cu^2+^ sites by varying the molecular structures of the building blocks (Supplementary Fig. 1), to determine the possible coordination groups. In some cases, we changed the hydrophobic segment of the amphiphilic amino acids from Fmoc to a tert-butoxycarbonyl (Boc) or benzyloxycarbonyl (Cbz) moiety to yield Boc-K or Cbz-K, or removed the hydrophobic segment to yield unmodified lysine (K). Alternatively, we altered the hydrophilic segment from lysine to arginine (R) or histidine (H) to yield Fmoc-R or Fmoc-H (other Fmoc-modified amino acids were not investigated, due to poor water solubility). Histidine is the main residue providing the coordinating groups for natural copper-cluster sites. We found that Boc-K/GMP/Cu^2+^ (g_//_, 2.254, A_//_, 184 × 10^-4^ cm^-1^) or Cbz-K/GMP/Cu^2+^ (g_//_, 2.349, A_//_, 157 × 10^-4^ cm^-1^) exhibited hyperfine splitting patterns and parameters almost identical to Boc-K/Cu^2+^ (g_//_, 2.251, A_//_, 181 × 10^-4^ cm^-1^) or Cbz-K/Cu^2+^ (g_//_, 2.299, A_//_,161 × 10^-4^ cm^-1^; Supplementary Fig. 6). On the other hand, Cu^2+^ that self-assembled with Fmoc-H/GMP (g_//_, 2.244, A_//_, 175 × 10^-4^ cm^-1^) or Fmoc-R/GMP (g_//_, 2.26, A_//_, 184 × 10^-4^ cm^-1^) hybrids showed spectral features and hyperfine parameters distinct from those of the individual components (Supplementary Fig. 7 and Supplementary Table 1). The EPR results indicate that self-assembly of Fmoc-modified amino acids with GMP altered the ligand environment of Cu^2+^ in a different manner from Boc- or Cbz-modified residues. The Fmoc fluorenyl group has stronger aromatic stacking and hydrophobic interactions than the Cbz and Boc groups, which lends Fmoc a stronger propensity for intermolecular self-assembly, and may also lead to more effective interactions of the amino acids with GMP. We verified the aromatic stacking of fluorenyl rings in Fmoc-containing amphiphiles by fluorescence emission at 384 nm (Supplementary Fig. 8). The fluorescence of pyrene (the intensity ratio of the first and third vibronic peaks, I_1_/I_3_) was used to probe the polarity of the microenvironments within the self-assembled complexes^40,41^. The I_1_/I_3_ values of pyrene in Fmoc-K/GMP/Cu^2+^ (~0.83) and Fmoc-K/Cu^2+^ (~0.87) were significantly lower than that in water (~1.69) and of other lysine-containing complexes, reflecting the greater hydrophobic association of the Fmoc moiety than Boc or Cbz (Supplementary Fig. 9 and Fig. 2e). Boc-K/GMP/Cu^2+^ (~2.67 Å, ~3.83 Å), Cbz-K/GMP/Cu^2+^ (~2.60 Å, ~3.69 Å) and Fmoc-R/GMP/Cu^2+^ (~2.59 Å, ~3.64 Å) also show two Cu-Cu distances, while Fmoc-H/GMP/Cu^2+^ only exhibits one Cu-Cu distance (~3.89 Å) (Supplementary Fig. 10), which indicates that T3Cu-mimietic dicopper sites were formed in these self-assemblies.

We changed the base portion of the nucleotide from guanine (GMP) to adenine, cytosine or uracil to yield adenosine monophosphate (AMP), cytidine monophosphate (CMP) or uridine monophosphate (UMP) (for molecular structures, see Supplementary Fig. 1). The N3 position of uracil must deprotonate (pKa 9.9) before it can form a moderate coordination with a metal, while the carbonyl oxygen has a weak affinity for metal^23^. The N7 position of guanine (pKa 2.1), N1 position of adenine (pKa 3.5) and N3 position of cytosine (pKa 4.2) have deprotonated sites for coordination to Cu^2+^, while the oxygen atoms exert a cooperative chelation effect^23^. As shown in Supplementary Fig. 11 and Supplementary Fig. 12, the Fmoc-K/CMP/Cu^2+^ (g_//_, 2.286, A_//_, 175 × 10^-4^ cm^-1^) and Fmoc-K/AMP/Cu^2+^ (g_//_, 2.291, A_//_, 175 × 10^-4^ cm^-1^) exhibit one Cu species with similar hyperfine parameters, although the CMP/Cu^2+^ shows a ^14^N superhyperfine pattern in the perpendicular (g _┴_) region corresponding to four nitrogen nuclei around the Cu^2+^ ion. It is noteworthy that Fmoc-K/UMP/Cu^2+^ shows two Cu species, the g and A values (g_//_, 2.308, A_//_, 167 × 10^-4^ cm^-1^; g_//_, 2.250, A_//_, 184 × 10^-4^ cm^-1^) of which were almost identical to those of Fmoc-K/Cu^2+^, indicating UMP may have a negligible effect on the coordinate environment of Fmoc-K/Cu^2+^. This may be attributed to the weak coordination of the UMP base to Cu^2+^^[ 23^, as indicated by the similar hyperfine parameter of UMP/Cu^2+^ to that of CuSO_4_, compared to GMP/Cu^2+^, CMP/Cu^2+^ and AMP/Cu^2+^. We then changed the monophosphate group to diphosphate, or triphosphate, to yield GDP or GTP, or removed the group to yield guanosine. We observed that the hyperfine parameters of the Fmoc-K/GTP/Cu^2+^ and Fmoc-K/GDP/Cu^2+^ complexes were almost the same as those of CuSO_4_ (Supplementary Table 1 and Supplementary Fig. 13). The additional phosphate groups in GDP and GTP may both localize Cu^2+^ within the nucleotide components, due to multi-dentate complexation and electrostatic attraction between the phosphate ions and Cu^2+^^[ 24^, and reduce the cooperation effect of the nucleobase and amino acid components. It is worth noting that our observation of EPR signals within Fmoc-K/guanosine/Cu^2+^ (g_//_, 2.252, A_//_, 181 × 10^-4^ cm^-1^), which were similar to Fmoc-K/GMP/Cu^2+^, indicates that the effective coordination groups of the nucleotides were from the base to form T2Cu, rather than the phosphate (Supplementary Fig. 14). However, the phosphate groups can improve the solubility of the nucleobases, enhance the association between the nucleobases and amphiphilic acids and buffer the pH of the reaction mixture. The EPR results show that the ∆g_//_ (g_//_ - g_e_) (e.g., ~0.348 for GMP/Cu^2+^, ~0.379 for Fmoc-K/GTP/Cu^2+^ and 0.389 for Fmoc-K/GDP/Cu^2+^) of the Cu^2+^ complexes containing GDP or GTP, or single nucleotide components, were higher than other complexes containing T2Cu-mimietic sites. The higher ∆g values indicate a lower crystal-field strength^42^ as a result of the phosphate groups replacing the π-acid type ligands, such as histidines in copper enzymes and nucleobases in our complexes, as the ligand coordinating to Cu^2+^^16^, and may also indicate that phosphate-Cu^2+^ binding was not dominant in the Fmoc-modified amino acids/monophosphate nucleotides (e.g., GMP) hybrids. Cu-Cu distances of <4.0 Å were also found in Fmoc-K/UMP/Cu^2+^ (Supplementary Fig. 15).

To further characterize the Cu^2+^ coordination, we obtained three-pulse electron spin echo envelope modulation (3P-ESEEM) spectra at a magnetic field that corresponds to the g_┴_ region of the continuous-wave EPR signal at X-band frequencies (∼9.5 GHz)^43–45^. The spectrum for Fmoc-K/Cu^2+^ (Supplementary Fig. 16a) had only one peak at twice the Larmor frequency (ca. 1.95 MHz), indicating a significantly weak hyperfine coupling between Cu^2+^ and a ^14^N from the side chain amino group or backbone amide. The GMP/Cu^2+^ spectrum (Supplementary Fig. 16b) showed three peaks (0.43 MHz, 0.92 MHz, and 1.58 MHz) in the range of 0–2 MHz, the sum of the former two lower frequencies was close to the highest one, indicating the nuclear quadrupole interactions of a weakly coupled ^14^N. The three peaks in the ESEEM spectrum are typical of the remote, moderately-coupled ^14^N from a guanine ring bound to Cu^2+^. It is evident that the ^14^N-Cu^2+^ coupling in Fmoc-K/Cu^2+^ was much weaker than that in GMP/Cu^2+^, indicating a greater distance between ^14^N and Cu^2+^ in the former complex. It is noteworthy that ESEEM is well suited for measuring weak hyperfine couplings, so the direct coordination of N7 of guanine to Cu^2+^ cannot be shown by the ESEEM spectra. The spectrum of Fmoc-K/GMP/Cu^2+^ (Fig. 2g) has two sharp peaks (~0.37 MHz and ~1.59 MHz) and one broad shoulder peak (~1.10 MHz) that arise from the single quantum (SQ) transition of ^14^N, and a broad feature around 4.00 MHz, due to the double quantum (DQ) transition of the ^14^N^45,46^. The ESEEM spectrum of Fmoc-K/GMP/Cu^2+^ was different from that of Fmoc-K/Cu^2+^ or GMP/Cu^2+^, and not a superposition of the two, indicating the contribution by both component ligands to the ^14^N coupling to Cu^2+^. Compared with Fmoc-K/Cu^2+^, the ESEEM spectrum of Fmoc-H/Cu^2+^ (Supplementary Fig. 17a) or Fmoc-R/Cu^2+^ (Supplementary Fig. 17b) shows four peaks typical of a weakly coupled ^14^N, which indicates greater contribution of imidazole of Fmoc-H and guanidino of Fmoc-R to ^14^N-Cu coupling than that of Fmoc-K. Similarly, Fmoc-K/Cu^2+^ (Supplementary Fig. 16a), Cbz-K/Cu^2+^ (Supplementary Fig. 17c) shows only one peak at twice the Larmor frequency in the ESEEM spectrum, again indicating a significantly weak coupled ^14^N from Cu^2+^. When these amphiphilic amino acids assembled with GMP, the ESEEM spectra were different from those of each component/Cu^2+^ (Supplementary Fig. 18). All simulation parameters (e^2^qQ/h and η) are presented in Supplementary Table 4. These ^14^N ESEEM results indicate: (i) Fmoc-H and Fmoc-R can provide nitrogen and oxygen as the coordinating atoms, while Fmoc-K can only provide oxygen; (ii) the self-assembly of the heterogeneous components can change the ligand environments of Cu^2+^. The peak at ca. 14.40 MHz for all ESEEM spectra are from the weakly coupled protons that may come from either solvent or the ligand^46^.

To gain further insight into the coordination of Cu^2+^ to the guanine ring, we conducted hydrogen nuclear magnetic resonance (^1^H-NMR) measurements of guanosine/Cu^2+^ in DMSO-d6. Line broadening and shifting are mainly observed on the NMR signals of hydrogen nuclei that are in close proximity to the paramagnetic Cu^2+^ ions. This is due to the interaction between the magnetic field of the unpaired electron of Cu^2+^ and the nearby NMR nucleus, which causes the relaxation time of those nuclei to become shorter^47–49^. As shown in Supplementary Fig. 19, after adding Cu^2+^, a broadening and shifting of the peak at ca. 7.93 ppm was observed, which was attributed to the hydrogen on C8 of guanine ring. This finding confirms the coordination of N7 of guanine to Cu^2+^. The hyperfine constant, A_iso_, is estimated to be 0.18 MHz from ^1^H-NMR shift (Details of the analysis and calculation can be found in methods “Estimation of hyperfine constant from NMR shifts”). No line broadening was observed for the hydrogens of Fmoc-K/Cu^2+^ (Supplementary Fig. 19b), indicating the hydrogens were away from Cu^2+^. A similar line broadening of the guanine ring was also observed for Fmoc-K/guanosine after adding Cu^2+^ (Supplementary Fig. 19a). Based on the results of ^1^H-NMR, cwEPR, and ESEEM, it can be reasoned that the coordination sphere of Cu^2+^ in Fmoc-K/guanosine/Cu^2+^ (or Fmoc-K/GMP/Cu^2+^) was composed of N7 of guanine base and carbonyl/carboxylate of the lysine moiety.

### Structural modeling

We performed density functional theory (DFT) calculations to understand the coordination of Cu^2+^ to Fmoc-K or GMP. The model complexes at the single-molecular level are presented in Supplementary Fig. 20. The NH_3_^+^ side chain of Fmoc-K, with a pKa of 10.79, poorly coordinates to Cu^2+^. Thus, Cu^2+^ is mainly coordinated to the carbonyl oxygen atoms (complex *i* model). Considering that the phosphate groups did not contribute to the formation of the copper center in Fmoc-K/GMP hybrids, Cu^2+^ may form the complexes with GMP in two ways (complex ii and/or iii; the phosphate is not shown). In model complex ii, Cu^2+^ is coordinated to the N3 atom and 2’-OH of the ribose moiety, while in complex iii, Cu^2+^ is coordinated to the N7 and O6 atoms. The 2’-OH is absent in the deoxyguanosine monophosphate (dGMP), while the EPR parameters (g_//_, 2.274, A_//_, 173 × 10^-4^ cm^-1^) of the Fmoc-K/*d*GMP/Cu^2+^ complex (Supplementary Fig. 21), reflecting the strength of the crystal field and the coordination environments, and the dicopper distances (2.57 and 3.63 Å, Supplementary Fig. 22), were almost identical to those of Fmoc-K/GMP/Cu^2+^. These results exclude the participation of the ribose (complex ii). Complexes i and iii constitute the basis of the construction of theoretical T2 and T3Cu models.

**Fig. 3:**
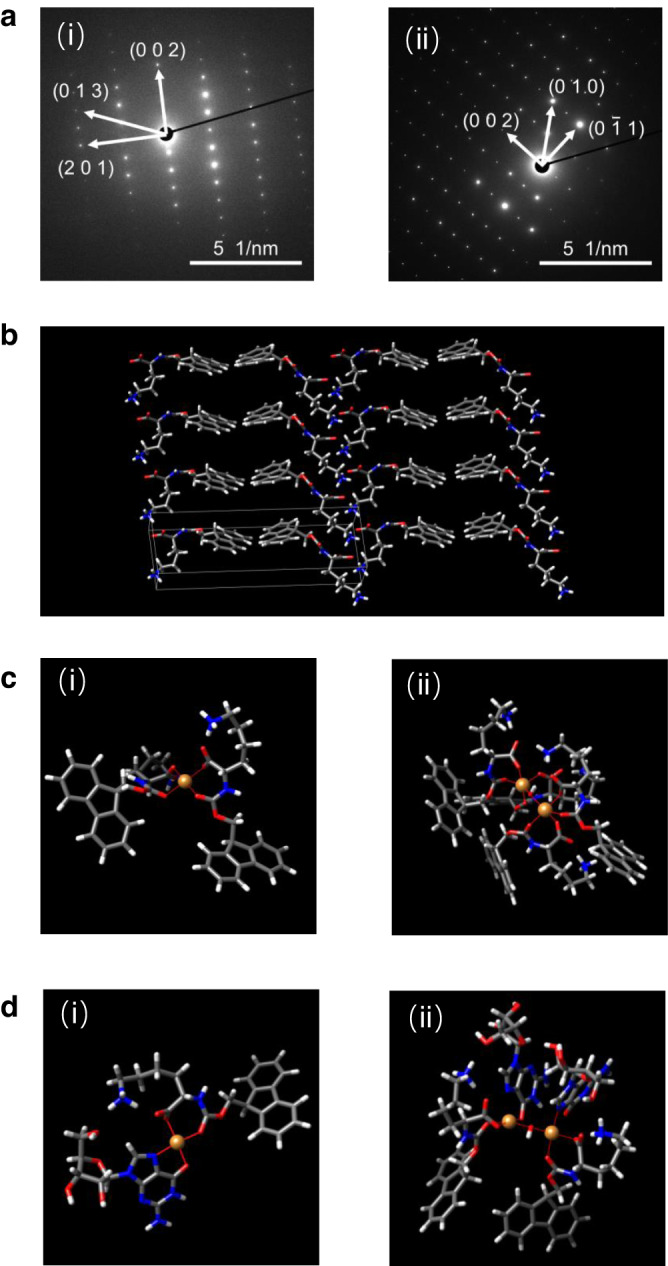
Structural model of the Fmoc-K crystal and Cu^2+^-containing complexes. **a** SAED patterns at different areas of the Fmoc-K crystal structure (0.1 M). **b** Theoretical model of the molecular packing in the Fmoc-K crystal. Each unit outlined in white contains two Fmoc-K molecules linked through π-stacking interactions. **c** Theoretical model of single copper (i) and dicopper (ii) (facet (1 0 0)) in the Fmoc-K self-assembly, based on the model in **b**. **d** Theoretical model of single copper (i) and dicopper (ii) in Fmoc-K/GMP self-assembly. N, O, C, H and Cu atoms are indicated in blue, red, dim gray, light gray and orange, respectively.

To reveal the Cu^2+^ center in the supramolecular materials, we investigated the self-assembled structures of Fmoc-K, by resolving the structures of nanoscale Fmoc-K crystals (Supplementary Fig. 23) that were formed after incubating an Fmoc-K aqueous solution for ca. six months. The typical selective-area electron diffraction (SAED) pattern recorded perpendicularly to the surfaces shows a single-crystalline nature of the self-assembled Fmoc-K structures (Fig. 3a and Supplementary Fig. 24). We then performed a first-principles calculation to elucidate the packing structure of the Fmoc-K molecules (Fig. 3b). The simulation revealed that Fmoc-K adopted a triclinic unit cell with the *P*1 space group and cell lengths, *a* = 4.593(8) Å, *b* = 7.281(8) Å, *c* = 24.841(2) Å, and angles, *α* = 96.1015°, *β* = 96.5859°, *γ* = 108.7390°. The diffraction peaks in the simulated XRD pattern (Supplementary Fig. 25) using this single-crystal data matched those of the SAED pattern (Fig. 3a and Supplementary Fig. 25) and low-temperature (120 K) powder XRD pattern (Supplementary Fig. 26). The Fmoc-K molecules assembled through π-π stacking, electrostatic interactions and hydrogen bonding. The Cu^2+^ can coordinate to Fmoc-K molecules along facet (0 0 1) (Supplementary Fig. 27) or (1 0 0) (Fig. 3c, i) to form a single-copper species with a coordination environment of distinct configuration and crystal-field strength, as observed by EPR (Supplementary Fig. 5). In the Fmoc-K aggregate, the neighboring copper sites along facet (0 0 1) or (1 0 0) (Fig. 3c, ii) can form a dicopper site (Supplementary Fig. 28), corresponding to the observed Cu-Cu distance of 2.59 Å or 3.73 Å (EXAFS, Fig. 2f). It is noteworthy that, in both aggregates, the dicopper of 3.73 Å is expected to be more catalytically active than that of 2.59 Å (also observed in CuO), in which the copper had a lower binding affinity to O_2_. The GMP that assembled with Fmoc-K slightly disturbed the fluorenyl-fluorenyl stacking (as observed from the fluorescence emission of Fmoc-K, Fig. 2c) leading to the coordination of Cu^2+^ to both GMP and Fmoc-K (Supplementary Fig. 29), and the formation of single-copper sites with a similar crystal field (as revealed by EPR, Fig. 2d). The coordination sphere of the single-copper site in GMP/Fmoc-K was composed of the N7 and O6 atoms from the guanosine moiety and the two carbonyl oxygen atoms from Fmoc-K (Fig. 3d, i). The dicopper sites (Fig. 3d, ii) formed in the Fmoc-K/GMP aggregate were similar to those in the Fmoc-K aggregate, as revealed by EXAFS (Fig. 2f), and had a distance of 3.76 Å. The ligands distributed around the single copper (T2) or dicopper (T3) sites can provide potential binding sites for Cu^2+^ to form additional catalytic centers, which may result in proximal copper arrangement, cooperating like a multinuclear copper site. No difference was observed in the CD (Supplementary Fig. 4) and fluorescence spectra (Supplementary Fig. 30) of the Fmoc-K/GMP hybrid in the presence or absence of Cu^2+^, indicating that the self-assembled structures were not affected by adding Cu^2+^. This suggests that the Fmoc-K or Fmoc-K/GMP self-assembly creates the framework of the coordination sphere for the copper sites. The nucleobase moiety that coordinates to Cu^2+^ may facilitate the proton transfer to the copper center during catalysis^14^. The Fmoc-H packing model was built with crystallized Fmoc-K as the reference (Supplementary Fig. 31), since the aggregation of these two amphiphilic amino acids can both be dominated by π-π stacking and hydrogen bonding interactions. However, Cu^2+^ coordination occurred on the facet (0 0 1), where flexible imidazole and carbonyl groups are distributed around the Cu^2+^ (Supplementary Fig. 32), leading to a greater coordination number of ligands to copper than that in Fmoc-K/Cu^2+^ (Supplementary Fig. 28). The side chain guanidinium of arginine also has coordination capability^50,51^. It can be concluded that the coordination of Cu^2+^ in Fmoc-R is similar to that in Fmoc-H aggregates.

### Structure-activity relationship

**Fig. 4:**
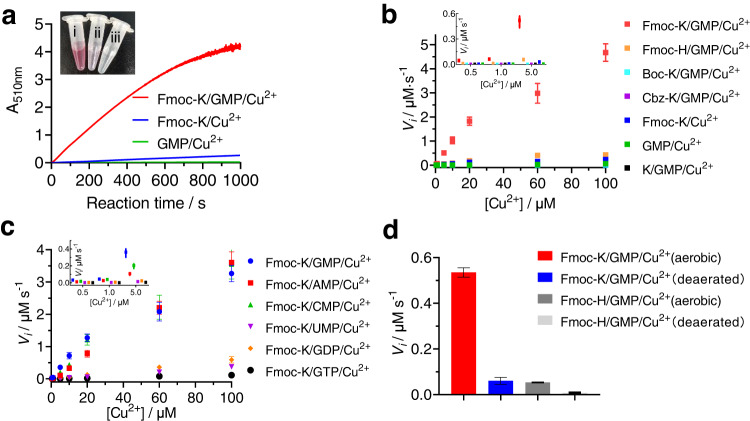
Catalytic activity of the Cu^2+^-containing complexes in 2,4-DCP oxidation, with O_2_ as the reducing substrate. **a** Time-dependent absorbance changes at 510 nm for the indicated complexes after addition of the substrates. Inset: reaction mixtures at 10 min after initiation. (i) Fmoc-K/GMP/Cu^2+^, (ii) Fmoc-K/Cu^2+^, (iii) GMP/Cu^2+^. [Cu^2+^] = 5 μM. **b**, **c** The initial catalytic velocities (*V*_i_) for the indicated Cu^2+^-containing complexes at various Cu^2+^ concentrations. **d** Initial catalytic velocities for Fmoc-K/GMP/Cu^2+^ and Fmoc-H/GMP/Cu^2+^ under aerobic or deaerated conditions. [Cu^2+^] = 5 μM. The data in panels b, c and d are presented as the mean ± s.d. (*n* = 3). [Fmoc-K] = 5 mM, [Boc-K] = 5 mM, [Cbz-K] = 5 mM, [K] = 5 mM, [Fmoc-H] = 5 mM, [GMP] = 10 mM, [2,4-DCP] = 1 mM, [4-AP] = 1 mM. The data in **b**, **c** and **d** are presented as the mean ± s.d., with the error bars representing the s.d. and *n* = 3 independent experiments.

We examined the catalytic activity of the self-assembled Cu^2+^ complexes with dissolved O_2_ and 2,4-dichlorophenol (2,4-DCP) as the substrates. The 2,4-DCP was oxidized into semiquinone radicals, which then reacted with 4-aminoantipyrine (4-AP) to produce a red adduct, with a maximum absorbance at 510 nm (Supplementary Fig. 33). The time-dependent-absorbance changes at 510 nm were recorded for the different Cu^2+^ complexes (Fig. 4a and Supplementary Fig. 34); the reaction rate catalyzed by the Fmoc-K/GMP/Cu^2+^ complex was significantly higher than that of Fmoc-K/Cu^2+^ or GMP/Cu^2+^. The initial catalytic velocity (*V*_i_) was estimated at different Cu^2+^ concentrations (Fig. 4b); the difference in *V*_i_ values was more pronounced at lower Cu^2+^ concentrations. At 5 μM Cu^2+^, the *V*_i_ values for the Fmoc-K/GMP/Cu^2+^, GMP/Cu^2+^ and Fmoc-K/Cu^2+^ complexes were 0.525 ± 0.049 μM s^-1^, 0.003 ± 0.001 μM s^-1^ and 0.031 ± 0.005 μM s^-1^, respectively. At 100 μM Cu^2+^, the respective *V*_i_ values were 4.675 ± 0.363 μM s ^-1^, 0.062 ± 0.001 μM s^-1^ and 0.232 ± 0.039 μM s^-1^. These results reveal a remarkable synergy between the GMP and Fmoc-K components in accelerating the catalysis by Cu^2+^.

To investigate the enzyme kinetics, we plotted *V*_i_ against the concentration of the reductants, in the presence of the Fmoc-K/GMP/Cu^2+^ complex or *Trametes versicolor* laccase. The global fitting approach was used to evaluate the turnover rate (*k*_cat_) and the catalytic efficiency (*k*_cat_ /*K*_m_) per Cu (Supplementary Fig. 35 to Supplementary Fig. 40 and Table 1). We evaluated the amount of Cu in the as-received laccase by inductively coupled plasma mass spectrometry (ICP-MS; 25.1 μg/g). With a chlorophenol (2,4-dichlorophenol [2,4-DCP], 2,4,6-trichlorophenol [2,4,6-TCP], 2.3,5,6-tetrachlorophenol [2,3,5,6-TCP], 3,3′,5,5′-Tetramethylbenzidine [TMB], 3,5-di-tert-butylcatechol [DTBC] or 2,6-dimethoxyphenol [DMP]) as the reducing substrate, the self-assembled complexes all exhibited lower *k*_cat_ and *k*_cat_ /*K*_m_ values than native laccase. It is notable that the *k*^TMB^_cat_ (1.3150 ± 0.0270 s^-1^) and *k*^DTBC^_cat_ (1.1960 ± 0.0370 s^-1^) of the Fmoc-K/GMP/Cu^2+^ complex reached the same order of magnitude to that of laccase (8.3038 ± 1.7595 s^-1^ and 5.3164 ± 0.3418 s^-1^), and *k*^DTBC^_cat_ / *K*^DTBC^_m_ (2.643 s^-1^ mM^-1^) was over 60% of that of laccase (3.7518 s^-1^ mM^-1^). Moreover, the obtained kinetic parameters were markedly greater than previously reported Cu^2+^ complexes in aqueous solution (Supplementary Table 5). The *k*_cat_ value of the catalyst towards the oxidization of DMP was ca. 2.95 s^-1^, which is 300-fold higher than that reported for an amyloid short peptide/Cu^2+^ complex, which also showed the EPR splitting features of T2Cu^21^. A markedly lower activity was observed for tyrosinase that only possess T3Cu sites (the copper amount was evaluated by ICP-MS; Supplementary Fig. 41). We conclude that the catalytic reactions may be synergistically accelerated by the T2Cu- and T3Cu-mimicking sites of the self-assembled complexes. The *k*_TMB_ cat value (1.3150 ± 0.0270 s^-1^) was almost 2-fold that of a recently reported, single-atom Fe nanoenzyme (0.7084 s^-1^) and 120-fold that of the commercially available Pt/C (0.0101 s^-1^)^52^. As shown in Supplementary fig. 34a, in the presence of Fmoc-K/GMP/Cu^2+^ (Cu^2+^,5 μM) and 2,4-DCP (1 mM)/4-AP (1 mM), the absorbance reached to the 4 when the catalyzed reaction proceeded for ca. 10 min. After calculating, one copper can promote oxidation of at least ca. sixty 2,4-DCP molecules, indicating Fmoc-K/GMP/Cu^2+^ is a catalyst. We also calculated the cost of the Fmoc-K/GMP/Cu^2+^ catalyst (ca $12 USD) with laccase (ca. $493 USD; the commercial sources are provided in the Materials and Methods section), with both catalyzing the oxidation of 2,4-DCP with a *V*_i_ value of 0.525 μM s^-1^ in 1 L aqueous solution, revealing that Fmoc-K/GMP/Cu^2+^ is considerably more cost-effective. We also tested the oxidase-mimetic function of a variety of transition metals complexed with the Fmoc-K/GMP hybrid, and found that only Cu^2+^ exhibited catalytic activity (Supplementary Fig. 42a). Moreover, the addition of other transition metals, at equimolar ratios to copper, to Fmoc-K/GMP/Cu^2+^ did not alter the *V*_i_ value, revealing a high specificity of the catalytic activity to Cu^2+^ (Supplementary Fig. 42b).

**Table 1:** Apparent kinetic parameters for laccase and Cu-containing artificial complexes with respect to the catalytic oxidation of a variety of reducing substrates

	*k*_cat_ (s^-1^)	*K*_m_ (mM)	*k*_cat_/*K*_m_ (s^-1^ mM^-1^)
Fmoc-K/Cu^2+^ (2,4-DCP)	0.0232±0.0060	5.1200±1.916	0.0045
GMP/Cu^2+^ (2,4-DCP)	0.0010±0.0002	2.6660±0.7310	0.0004
Fmoc-K/GMP/Cu^2+^ (2,4-DCP)	0.2236±0.0120	0.6962±0.1200	0.3211
Fmoc-H/GMP/Cu^2+^ (2,4-DCP)	0.0751±0.0291	6.7900±3.0000	0.0111
Laccase (2,4-DCP)	5.924±0.1266	0.2912±0.0314	20.6200
Fmoc-K/GMP/Cu^2+^ (2,4,6-TCP)	1.0960±0.0880	0.2494±0.0540	4.6908
Laccase (2,4,6-TCP)	33.7720±1.038	0.2677±0.0351	128.8610
Fmoc-K/GMP/Cu^2+^ (2,3,5,6-TCP)	0.0800±0.0052	0.0683±0.0047	1.1826
Laccase (2,3,5,6-TCP)	1.767±0.5949	0.0440±0.0032	41.3633
Fmoc-K/GMP/Cu^2+^ (TMB)	1.3150±0.0270	0.8018±0.1150	1.6800
Laccase (TMB)	8.3038±1.7595	0.2374±0.1092	48.7215
Fmoc-K/GMP/Cu^2+^ (DTBC)	1.1960±0.0370	0.4530±0.0150	2.4247
Laccase (DTBC)	5.3164±0.3418	1.417±0.1370	3.1801
Fmoc-K/GMP/Cu^2+^ (DMP)	2.9500±0.8820	19.5700±8.4800	0.1507
Laccase (DMP)	15.6835±0.6139	0.0229±0.0029	684.2785

During catalysis by laccase, the clustered Cu^2+^ ions accept electrons from the reducing substrates to transform into Cu^+^ ions, which are then oxidized by O_2_ to return to Cu^2+^^53^. The observed synergistic activity in the self-assembled Fmoc-K/GMP/Cu^2+^ complex may be due to the enhanced reactivity of Cu with O_2_. We deaerated the catalytic system containing Fmoc-K/GMP/Cu^2+^ and 2,4-DCP/4-AP, and found that the formation rate of the red adduct decreased by over 14-fold (Fig. 4d), confirming the key role of O_2_ in the reactions. To understand the structure-activity relationship, we examined the effects of the molecular structures of the components on the initial velocity of the reactions. With 2,4-DCP as the substrate, the *V*_i_ value of Fmoc-K/GMP/Cu^2+^ was 9.8-fold higher than Fmoc-H/GMP/Cu^2+^ (at 5 μM Cu^2+^; Fig. 4b); when the GMP and Fmoc-K (or Fmoc-H) were 10 mM and 5 mM, respectively, the *k*_cat_/*K*_m_ value of Fmoc-K/GMP/Cu^2+^ was ca. 30-fold higher than that of Fmoc-H/GMP/Cu^2+^ (Supplementary Fig. 43 and Table 1). The activity of Fmoc-R/GMP/Cu^2+^ was slightly higher than that of Fmoc-H/GMP/Cu^2+^, but also significantly lower than that of Fmoc-K/GMP/Cu^2+^ (Supplementary Fig. 44). No activity was observed for the Cu^2+^ complexes containing Boc-K and Cbz-K. All these materials contained dicopper and single copper sites. On the other hand, it is noteworthy that the natural copper sites are coordinatively unsaturated, allowing for O_2_ binding to the copper clusters and O_2_ reduction, as a result of the well-defined protein folding. As mentioned above, the considerably weaker aromatic stacking and hydrophobic interactions between Boc-K or Cbz-K molecules (Fig. 2e and Supplementary Fig. 45) than those between Fmoc-K molecules may lead to random distribution of the amino acids around Cu^2+^ and the coordination saturation of the metal. Moreover, there is no simultaneous coordination of Cu^2+^ to both Boc-K (or Cbz-K) and GMP, as observed from hyperfine parameters of EPR (Supplementary Table 1), leading to the inefficient catalytic cooperation between these two components. These two factors may lead to the significantly low activity of Boc-K/GMP/Cu^2+^ and Boc-K/GMP/Cu^2+^. The Fmoc-H or Fmoc-R aggregates showed a greater number of ligands around Cu^2+^ than Fmoc-K, leading to the lower activity of Fmoc-H/GMP/Cu^2+^ and Fmoc-R/GMP/Cu^2+^ than Fmoc-K/GMP/Cu^2+^, and Fmoc-H/Cu^2+^ than Fmoc-K/Cu^2+^. The catalytic activity of Fmoc-H/Cu^2+^ and Fmoc-H/GMP/Cu^2+^ was still significantly higher than Boc-K/GMP/Cu^2+^, Cbz-K/GMP/Cu^2+^, Boc-H/GMP/Cu^2+^, and Cbz-H/GMP/Cu^2+^, illustrating the importance of the ordered aromatic packing of the fluorenyl groups to the formation of the highly active copper site.

The *V*_i_ value decreased slightly for the Cu^2+^ complexes containing GMP, CMP and AMP (Fig. 4c), following the order of base coordination affinity to Cu^2+^^23^, when the Cu^2+^ concentration was below 10 μM. At higher Cu^2+^ concentrations, these complexes showed almost identical activities. In comparison, Fmoc-K/UMP/Cu^2+^ was markedly less active. With varying numbers of phosphate, Fmoc-K/guanosine/Cu^2+^ and Fmoc-K/GMP/Cu^2+^ showed equal activities (Supplementary Fig. 46), while the activity of Fmoc-K/GDP/Cu^2+^ was much lower, and Fmoc-K/GTP/Cu^2+^ was not active (Fig. 4c). The EPR results demonstrate that (i) UMP only had negligible effect on the coordination environments of Cu^2+^ in the Fmoc-K aggregates; (ii) The addition of GDP and GTP to Fmoc-K resulted in the localization of Cu^2+^ to the phosphate environments because of strong Cu^2+^-phosphate binding. These results indicate that the coordination of Cu^2+^ to both Fmoc-K and nucleotide components is important to the oxidase activity of the Cu^2+^.

Despite their different activities, Fmoc-K/GMP/Cu^2+^ and Fmoc-H/GMP/Cu^2+^ had comparable apparent activation energy (71.708 kJ mol^-1^ and 72.032 kJ mol^-1^), as calculated using the Arrhenius equation (Supplementary Fig. 47)^54–56^, indicating that the reactions catalyzed by these two complexes had similar intermediate species and reaction paths. The other less-active or non-active complexes, including Fmoc-K/Cu^2+^ (75.566 kJ mol^-1^), GMP/Cu^2+^ (82.217 kJ mol^-1^), Fmoc-H/Cu^2+^ (125.566 kJ mol^-1^), Fmoc-K/GTP/Cu^2+^ (78.614 kJ mol^-1^), Fmoc-K/UMP/Cu^2+^ (80.513 kJ mol^-1^) and Cbz-K/GMP/Cu^2+^ (77.254 kJ mol^-1^), exhibited higher *E*_a_ values.

The inhibitory effect of the imidazole on the binding of the exogenous ligand was further revealed by adding NaN_3_ to the catalytic system. NaN_3_ has previously been used as an anion inhibitor of multicopper oxidases (e.g., laccase, ascorbate oxidase), that binds T2Cu and bridges T2Cu to T3Cu^57,58^. Excessive NaN_3_ almost fully deactivated the native laccase, but unexpectedly, increased the activity of Fmoc-K/GMP/Cu^2+^ (Fmoc-K 5 mM, Fmoc-K/GMP: 1/2) by ca. 25% (Supplementary Fig. 48a). The absorbance in the range of 350 nm–450 nm increased (Supplementary Fig. 48b), which can be attributed to the N_3_^−^→Cu^2+^ charge transition^59^. When the concentrations of Fmoc-K and GMP were decreased, the activity enhancement (Supplementary Fig. 48c), as well as absorbance change upon addition of N_3_^−^ (Supplementary Fig. 48d), were more pronounced. We also found that N_3_^-^ can enhance the activity of Fmoc-K/Cu^2+^, but less significantly than GMP (Supplementary Fig. 49). We conclude that the N_3_^-^-induced enhancement of Fmoc-K/GMP/Cu^2+^ activity can be ascribed to the coordination of a greater quantity of Cu^2+^ to both ligands, Fmoc-K and GMP (or N_3_^-^). In contrast to Fmoc-K/GMP/Cu^2+^, N_3_^-^ deactivated both Fmoc-H/GMP/Cu^2+^ and Fmoc-H/Cu^2+^, which was more evident at lower Fmoc-H concentrations (Supplementary Fig. 50). The UV-vis spectra show only slight N_3_^−^→Cu^2+^ charge transitions, compared to Fmoc-K/GMP/Cu^2+^ with N_3_^−^ (Supplementary Fig. 51). The mechanism of N_3_^-^-induced deactivation is probably due to the hydrogen bonding of N_3_^-^ to the imidazole of the neighboring Fmoc-H, which leads to an enhancement of the basicity of the imidazole and its coordination to copper, and decreased N_3_^-^ coordination capability. N_3_^-^ decreased the intensity of the fluorescence emission of stacked Fmoc-His at 384 nm, while not affecting that of Fmoc-K (Supplementary Fig. 52). Meanwhile, the multivalent, strong coordination of Fmoc-His to Cu^2+^ also disfavors the Cu^2+^-N_3_^-^ binding.

### Catalytic mechanism

**Fig. 5:**
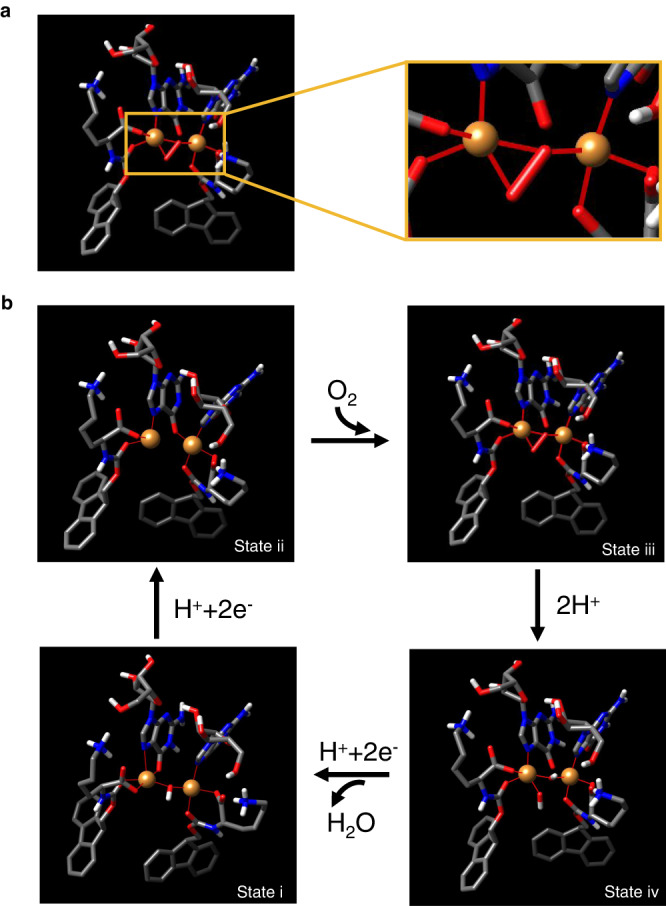
Catalytic mechanism of the Fmoc-K/GMP/Cu^2+^ complex. **a** Density functional theory model of the dicopper/O_2_ reactive intermediate. Inset illustrates the coordination bonding between the copper and O and N atoms. **b** Proposed catalyzed turnover by the dicopper-mimetic site, including the reduction of the dicopper site, reaction with O_2_, cleavage of the peroxide bond and the reduction of O_2_ to water. States i and iii were obtained from DFT calculations. N, O, C, H and Cu atoms are indicated in blue, red, dim gray, light gray and orange, respectively.

To further understand the catalytic mechanism of the enzyme mimic, we performed density functional theory (DFT) to simulate the adsorption of O_2_ to the dicopper site (Fig. 5a), which represents the minimal unit of copper cluster in the aggregate. Successful O_2_ adsorption is a key event leading to the subsequent catalytic reactions. The O-O bridged the copper to form a peroxide species. Figure 5b shows the possible pathway for the catalyzed O_2_ reduction, with the catalytic mechanism of laccase as a refs. ^14,60–62^. The reaction begins when the reductant (e.g. 2.4-dichorophenol, 2,4,6-trichlorophenol, 3,3’,5,5’-tetramethylbenzidine or 3,5-ditert-butyl catechol) donates two electrons to reduce the Cu^2+^ to Cu^+^ (State i to ii). Next, the reduced dicopper reacts with O_2_ to generate a “peroxide intermediate” (PI; State iii), where the two copper atoms are oxidized and have reduced dioxygen by two electrons. The peroxide intermediate decays to the native intermediate (NI; State iv), by accepting two electrons from other reductants, transferring two protons and cleaving the O-O bond. The NI can decay to the resting oxidized (RO) form, which is reduced to start a new catalytic cycle. In short, the dicopper site couples four 1*e*^−^ oxidations of the substrates to the 4*e*^−^ reduction of O_2_ to water. We also modeled the adsorption of O_2_ to trinuclear copper sites (Supplementary Fig. 53), which resembles the peroxide intermediates of T2/T3Cu in natural multicopper oxidases^14^. We propose the role of GMP in the catalysis as follows: (i) The transformation of the copper site from Cu-O4 to Cu-O3(N), as a result of the assembly of GMP with Fmoc-K/Cu^2+^, led to the enhancement of the activity. It has been widely reported that the reactivity of the metal center exhibits dependence on the coordinating atoms (e.g., C, O, N, P, Cl, S)^63–67^. (ii) The guanine base may assist the hydrogen transfer during the catalysis (State iii to iv), similar to COO^-^^14,68^, imidazole^69–72^, etc. The lower turnover rate of the artificial complexes, in comparison to laccase, can be attributed to the following: (i) some of the copper ions that were added did not form the catalytically active T2-/T3-mimetic sites; (ii) the absence of T1Cu, which may significantly retard the decay rate of the intermediate conversion^14,73^, and the redox potential of the copper cluster^16^.

### Structural factors to the catalytic efficiency

Taking the experimental and simulation results above together, it is apparent that Fmoc-K self-assembly, and the heterogeneity of the molecular building blocks, were indispensable to the construction of the superior catechol oxidase-mimicking, Cu^2+^-containing catalysts, which can be attributed to the following structural characteristics of complexes: First, the aromatic packing of the fluorenyl groups of Fmoc-K allowed the ordered distribution of the ligand groups (carbonyl groups) around Cu^2+^, which facilitated the copper clustering and prevented the coordination saturation of Cu^2+^. This led to the higher activity of Fmoc-K/Cu^2+^ (*k*_cat_, 0.0232±0.006 s^-1^) than that of GMP/Cu^2+^ (~0 s^-1^), Boc-K/Cu^2+^ (~0 s^-1^) and Cbz-K/Cu^2+^ (~0 s^-1^), and cooperation between Fmoc-modified amino acids and other ligands (e.g., nucleotides). Second, there was only an appropriate number of ligands from the rigid amino acid backbones of Fmoc-K to coordinate Cu^2+^, leading to the potentially lower coordination number of Cu^2+^ in the Fmoc-K aggregates, than that in Fmoc-R or Fmoc-H, that also can use the side chain group (guanidinium or imidazole) as the ligand. Therefore, the complexes containing Fmoc-K had much higher activity than those containing Fmoc-R or Fmoc-H. Third, the moieties of the heterogeneous ligand components exhibited comparable Cu^2+^ binding capacity, which allowed the coordination of Cu^2+^ to both components, leading to a synergy between the copper clustering effect (e.g., Fmoc-K) and proton transfer effect (e.g., nucleotides). Thus, GMP elicited a more remarkable enhancement of Fmoc-K/Cu^2+^ activity, compared to other mononucleotides (e.g., CMP, AMP, UMP) which possess weaker Cu^2+^ coordination capacity, or GDP and GTP that can extract Cu^2+^ from the Fmoc-K. It is noteworthy that, in contrast to Fmoc-K, the activity of Fmoc-H/Cu^2+^ can only be enhanced by GMP, and not CMP or AMP (Supplementary Fig. 54), which can be attributed to the strong Fmoc-H/Cu^2+^ binding.

### Stability

**Fig. 6:**
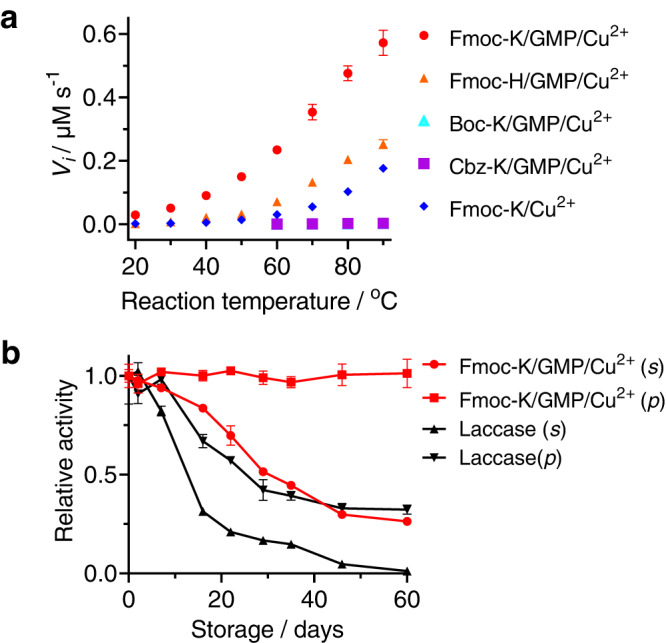
Catalytic stability of the Cu^2+^-containing complexes with 2,4-DCP as the reducing substrate. **a** Temperature-dependent catalytic velocities of the various complexes. [Cu^2+^] = 0.3 μM. **b** Storage stability of the solution-state (*s*) and powder-state (*p*) of the copper complex and laccase at room temperature. [Cu^2+^] = 5 μM, [laccase] = 5 μM, [Fmoc-K] = 5 mM, [Fmoc-H] = 5 mM, [Boc-K] = 5 mM, [Cbz-K] = 5 mM, [GMP] = 10 mM, [laccase] = 5 μM, [2,4-DCP] = 1 mM, [4-AP] = 1 mM. The data in **a** and **b** are presented as the mean ± s.d., with the error bars representing the s.d. and *n* = 3 independent experiments.

The noncovalent bonds that maintain enzyme folding usually break down when heated, resulting in protein denaturation and activity loss. Native laccase showed optimal activity between 50 °C and 60 °C and fully deactivated at 80 °C (Supplementary Fig. 55). In contrast, the GMP/Fmoc-K/Cu^2+^ complex showed thermophilic catalytic activity in the range of 20 °C to 90 °C, an almost 50-fold enhancement (Fig. 6a). At 80 °C, the oxidase activity of the complex was observed at as low as 20 nM Cu^2+^ (Supplementary Fig. 56). The increase in temperature also led to enhancement of the activity of other Cu^2+^ complexes (i.e., 126-fold for Fmoc-H/GMP/Cu^2+^, 90-fold for Fmoc-R/GMP/Cu^2+^ and 91-fold for Fmoc-K/guanosine/Cu^2+^), with the exceptions of GMP/Cu^2+^, Boc-K/GMP/Cu^2+^, Fmoc-K/GTP/Cu^2+^ and Cbz-K/GMP/Cu^2+^ (Supplementary Fig. 57 and Fig. 6a). Fluorescence spectrometry of the complexes revealed that the catalytic assemblies were only partially dissociated or the aggregation states changed upon heating (Supplementary Fig. 58). It is likely that elevating the temperature resulted in less compact stacking of the fluorenyl rings and molecular association, along with enhanced mobility of the bond vibration. Thus, the requisite arrangement of the coordination atoms around Cu^2+^ was facilitated. We also examined the Fmoc-K/GMP/Cu^2+^ complex over different incubation times at 80 °C (Supplementary Fig. 59). About 60% of the activity of the artificial complex was retained after 5 h. Full deactivation of laccase was observed when the aggregate was incubated at its optimal temperature (60 °C) for 30 min. On the other hand, the catalytic complex exhibited reversible activity when alternating between 25 °C and 80 °C (and a 31.7% activity retention at both temperatures after 200 cycles; Supplementary Fig. 60), while laccase almost completely lost its activity after 18 cycles. Similarly, we investigated the response of the activities to acidification by cycling the pH between pH 6.0 and pH 2.6 (Supplementary Fig. 61). No loss of activity was found after over ten cycles, but laccase was completely inactive after 5 cycles. We also compared the storage stabilities of Fmoc-K/GMP/Cu^2+^ and laccase maintained in aqueous solution or as lyophilized powder at room temperature. The solution-state copper complex showed a moderately higher retention of activity than laccase, while the powder-state complex did not show inactivation over as long as 60 days (Fig. 6b). We also investigated the tolerance of the Fmoc-K/GMP/Cu^2+^ to high concentrations of salt (i.e., NaNO_3_), and found the activity was even moderately increased when NaNO_3_ reached 1 M (Supplementary Fig. 62).

## Discussion

In summary, here we report a simple strategy for the de novo design and fabrication of a supramolecular catalyst containing copper-cluster active sites and mimicking the catalytic functions of a catechol oxidase. The activities surpassed previously reported artificial catalysts and were significantly accelerated by increasing the reaction temperature. The catalysts also showed markable tolerance to temperature or pH cycling and room-temperature storage, compared to native enzymes. The superior thermophilic activities may enable the potential use of such enzyme mimics in the decolorization of textile wastewater, which is a common, high-temperature effluent system, and/or in the conversion of biomass (e.g., lignin) that can be softened and swelled at elevated temperatures. This work may pave the way for reconstructing versatile metallocluster active sites and expanding the application of the biocatalysts. These catalysts, based on the self-assembly of small molecules (*M*_w_ < 410 Da), provide a putative model for primitive enzymes, whose thermophilic behaviors and environment-induced deactivation and activation may explain how these molecular assemblies survived under harsh, prebiotic conditions.

## Methods

### Materials

Fmoc-Lysine-OH·HCl(Fmoc-K), Fmoc-Histidine-OH(Fmoc-H), Fmoc-Arginine-OH(Fmoc-R), Boc-Lysine-OH(Boc-K), L-Lysine hydrochloride(K), Guanosine monophosphate(GMP), Adenosine monophosphate(AMP), Cytidine monophosphate(CMP), Uridine monophosphate(UMP), Guanosine, 2'-Deoxyguanosine 5'-monophosphate(dGMP), 4-Aminoantipyrine(4-AP), 3,3',5,5'-Tetramethylbenzidine(TMB), 2,6-Dimethoxyphenol(2,6-DMP), 3,5-Di-tert-butylcatechol(3,5-DTBC), Copper sulfate pentahydrate(CuSO_4_·5H_2_O), Iron chloride hexahydrate(FeCl_3_·6H_2_O), Cobalt chloride hexahydrate(CoCl_2_·6H_2_O), Manganese chloride tetrahydrate(MnCl_2_·4H_2_O), Magnesium chloride hexahydrate(MgCl_2_·6H_2_O), Aluminum chloride(AlCl_3_), 2,4,6-Trichlorophenol(2,4,6-TCP), Nickel chloride hexahydrate(NiCl_2_·6H_2_O), Zinc chloride(ZnCl_2_), 2.3,5,6-Tetrachlorophenol(2,3,5,6-TetraCP) and Boc-Histidine-OH(Boc-H) were purchased from Aladdin (China). Guanosine diphosphate(GDP), Guanosine triphosphate(GTP), Laccase and Tyrosinase were purchased from Yuanye (China). Cbz-Lysine-OH(Cbz-K), Pyrene, Ferrous chloride tetrahydrate(FeCl_2_·4H_2_O), Calcium chloride(CaCl_2_), Cadmium Chloride(CdCl_2_), Chromic chloride hexahydrate(CrCl_3_·6H_2_O), Scandium chloride hexahydrate(ScCl_3_·6H_2_O), 2,4-Dichlorophenol(2,4-DCP) and Vanadium chloride(VCl_4_) were purchased from Innochem (China). Water was deionized using a Milli-Q system(≥18.25 MΩ·cm^–1^). Fmoc-K, Fmoc-H, Fmoc-R, Boc-K, Cbz-K, Boc-H, Cbz-H, K and H (0.1 M) were dissolved in ultrapure water in the presence of 0.1 M hydrochloric acid. The solutions were kept for over 2 weeks priror to further use.

### Instruments

UV-Vis absorption spectra were recorded using a UV-2600 spectrometer equipped with a temperature-control accessory (Shimadzu). Fluorescent emission spectra were recorded using a G9800A fluorescence spectrophotometer with a temperature-control accessory (Agilent Technologies). SEM characterization was conducted using a Supra microscope at 5.0kv (Zeiss). TEM characterization was conducted on a Hitachi 7800 microscope in bright-field mode at 80kV. SAED were carried out on a JEM-F200 system with cooling stage and energy-dispersive X-ray spectrometer (JEOL). AFM characterization was conducted on a Nano Wizard 4 BioScience (JPK). CD spectra were recorded with a J-815 spectropolarimeter (Jasco) under the following conditions: optical path, 0.5mm, bandwidth, 10nm, scan speed, 50nm/min. EPR measurements were conducted on an ELEXSYS-II E500 EPR spectrometer with a low-temperature accessory (Bruker BioSpin). Three-pulse EPR measurements were conducted on an EPR100 X-band pulsed EPR spectrometer (CIQTEK) with a variable temperature controlled dry liquid helium-free cryogenic system (CIQTEK). Powder XRD patterns were collected on a Smartlab-9KW diffractometer equipped with a copper filter (Rigaku) under the following conditions: scan speed, 5 min^-1^, Cu Kα radiation, λ = 1.54 Å, temperature, 120 K.

XAFS were collected at 1W1B station in Beijing Synchrotron Radiation Facility (BSRF) with fluorescence mode. The acquired EXAFS data were processed using the ATHENA module implemented in the IFEFFIT software packages^74^. 1H NMR were recorded in DMSO-d6 on Bruker AVANCE III HD 400 MHz. The true concentrations of enzyme were conducted using an XII ICP-MS (ThermoFisher) after microwave digesting on a Mars 6 equipment (CEM).

### Sample preparation for characterization

(1) For obtaining SEM images, we applied the self-assembled sample solution onto a silicon wafer and allowed it to settle for 20 min. Any unbound sample was then removed by wicking it away. Prior to observation, a thin layer of platinum particles was applied to the surface to ensure sample conductivity by the gold spray instrument with 15 s. (2) To prepare the sample for TEM imaging, we deposited the self-assembled solution onto a hydroxylated copper sheet and allowed it to sit for 20 min. Excess solution was removed using filter paper, and the remaining solution was left to evaporate. (3) For AFM imaging, 5 μL of the solution was carefully placed onto freshly cleaved mica surfaces. This was followed by a gentle wash with double distilled water and subsequent drying under a nitrogen flow.

### Activity assay

The amino acid amphiphiles (Fmoc-K, Fmoc-H, Fmoc-R) were dissolved in ultrapure water to prepare 100 mM stock solutions, which were stored for over 2 weeks before use. Subsequently, the required concentrations of amino acid amphiphiles, nucleotide, and copper ions were added to ultrapure water. The mixture was then incubated at room temperature for 12 h. Using 2,4-DCP/4-AP or other substrates (with a molar extinction coefficient of 13.6 mM^−1^ cm^−1^ for the adduct of the oxidized 2,4-DCP with 4-AP)^75,76^, the reactions were conducted at ~pH 6.00. Time-dependent absorbance changes were recorded, and this data was used to determine the initial catalytic velocity (*V*_i_) and apparent kinetic parameters.

### Fluorescence assay

The amino acid amphiphiles (Fmoc-K, Fmoc-H, Fmoc-R) were dissolved in ultrapure water to prepare 100 mM stock solutions, which were stored for over 2 weeks. Subsequently, the required concentrations of amino acid amphiphiles, nucleotide, and copper were added to ultrapure water. The mixture was then incubated at room temperature for 12 h. The experimental conditions involved an excitation wavelength of 290 nm and an emission wavelength ranging from 300 nm to 350 nm, as well as an excitation wavelength of 320 nm and an emission wavelength ranging from 350 nm to 450 nm. The scan rate was set at 120 nm/min.

### Pyrene-involved experiment

To prepare the pyrene stock solution, 0.5 mg of pyrene was dissolved in 5 mL of methanol. For the fluorescence assay, the sample was mixed with the pyrene stock solution in a volume ratio of 100:1 immediately before analysis. The experimental conditions were set as follows: an excitation wavelength (λ_ex_) of 334 nm, an emission wavelength (λ_em_) ranging from 360 nm to 400 nm, and a scan rate of 120 nm/min. Two distinct fluorescent peaks, labeled as I_1_ and I_3_, were observed at 373 nm and 384 nm, respectively. The intensity ratio (I_1_/I_3_) between these two peaks can be utilized to investigate the polarity of the microenvironments within the self-assembled complexes.

### Deaeration treatment

The catalyst solution and substrate solution were placed in separate containers equipped with rubber stoppers. The Schlenk system was employed to evacuate air from the containers and replace it with argon gas, a procedure that was repeated three times. Subsequently, a continuous flow of argon was maintained for 30 min. For activity assays or UV-vis spectra measurements, a needle was utilized to rapidly transfer the substrates into the sample solution.

### Continuous-Wave Electron Paramagnetic Resonance (cw-EPR) spectroscopy

The samples were transferred to EPR tubes and frozen using a liquid nitrogen atmosphere for subsequent EPR experiments. cw-EPR spectra were collected using a Bruker ELEXSYS-II E500 CW-EPR spectrometer equipped with an Air Products cryostat and temperature controller modified for nitrogen gas flow sample cooling. The spectra were obtained under the following conditions: microwave frequency set at 9.41 GHz, microwave power at 10 mW, modulation amplitude at 2 Gauss, modulation frequency at 100 kHz, sweep time at 120 s, and temperature at 77 K. To ensure accuracy, a minimum of 5 scans were recorded and averaged for each spectrum. The simulations were conducted using the MATLAB R2021a toolbox Easyspin 5.2.35 by the function ‘Pepper’.

### Electron Spin Echo Envelope Modulation (ESEEM) spectroscopy

The samples were transferred to 4 mm outer diameter quartz EPR tubes and frozen using a liquid helium atmosphere (6K) for subsequent ESEEM experiments. Three-pulse ESEEM spectra were collected using a CIQTEK EPR100 spectrometer equipped with a liquid helium circulation system for helium gas flow sample cooling. The signal was recorded by measuring the stimulated electron spin echo intensity as a function of T using the pulse sequence: (π/2) - τ - (π/2) - T - (π/2) - τ - echo on the Cu-complex, where τ is the dephasing time and T is the waiting time. The duration of the (π/2) pulses for the Cu-complex is indicated in the figure captions. The interpulse delay τ was fixed at 200 ns. The time interval T was incremented from 100 ns with a step size of 16 ns, resulting in a total of 256 points. The microwave power was set to 200 mW, and at least 100 waveforms were accumulated for each sample. The envelope modulation was cosine Fourier transformed to generate ESEEM frequency spectra. Data processing and analysis were performed using EPR Data Processing (Version 4.1.6, CIQTEK), and simulations were conducted using the MATLAB R2021a toolbox Easyspin 5.2.35 by the function ‘saffron’.

### Estimation of hyperfine constant from NMR shift

When dealing with a paramagnetic complex, the NMR chemical shifts of the ligand nuclei experience a change ${\Delta }_{{{{{{\rm{CS}}}}}}}={\Delta }_{{{{{{\rm{HFI}}}}}}}+{\Delta }_{{{{{{\rm{Dia}}}}}}}+{\Delta }_{{{{{{\rm{PC}}}}}}}$. The three terms correspond to the hyperfine, diamagnetic and pesudocontact shifts. The first term, due to the hyperfine (magnetic) field generated by the unpaired electron(s), is typically 2–3 orders of magnitude larger than the latter two when the paramagnetic center is close to the nucleus concerned. Therefore, as a crude approximation ${\Delta }_{{{{{{\rm{CS}}}}}}}\approx {\Delta }_{{{{{{\rm{HFI}}}}}}}$. It’s worth noting that NMR relaxation is typically much slower than EPR, which means that the field that the nuclei “feel” from the unpaired electron(s) is an average value over time. From the hyperfine interaction Hamiltonian

$${\hat{H}}_{{HFI}}=\hat{S}\cdot {{{{{\boldsymbol{A}}}}}}\cdot \hat{I}$$

Averaging the electron spin (denoting the average by angle brackets), it becomes

$${\hat{H}}_{{HFI}}=\left\langle \hat{S}\right\rangle \cdot {{{{{\boldsymbol{A}}}}}}\cdot \hat{I}$$

Because the molecules in a solution are tumbling quickly, the apparent chemical shift corresponds to an angle-averaged value. In a strong external magnetic field in the z direction such as that in an NMR instrument, the spin averages to zero in the x and y directions, and thus the above simplifies to $\left\langle {S}_{z}\right\rangle {A}_{{iso}}{I}_{z}$ . Therefore, the absolute value of the hyperfine shift for an $I=\frac{1}{2}\,({I}_{z}=\pm \frac{1}{2})$ nucleus, such as ^1^H, is

$${\Delta }_{{{{{{\rm{CS}}}}}}}={\hat{H}}_{{HFI}}\,\left({I}_{z}=\frac{1}{2}\right)-{\hat{H}}_{{HFI}}\,\left({I}_{z}=-\frac{1}{2}\right)=\left\langle {S}_{z}\right\rangle {A}_{{iso}}\times \left[\frac{1}{2}-\left(-\frac{1}{2}\right)\right]=\left\langle {S}_{z}\right\rangle {A}_{{iso}}$$

or

$${A}_{{iso}}={\Delta }_{{{{{{\rm{CS}}}}}}}/\left\langle {S}_{z}\right\rangle$$

For a 400 MHz NMR, the magnetic field is 9.4 T. The EPR transition frequency for a *g* = 2.1 species in such a magnet is $2.1\times 9.4\times 1{0}^{4}{{{{{\rm{gauss}}}}}}\div714.48\frac{{{{{{\rm{gauss}}}}}}}{{{{{{\rm{GHz}}}}}}}=276\,{{{{{\rm{GHz}}}}}}.$ (714.48 gauss/GHz is Planck’s constant divided by Bohr magneton). Per Boltzmann distribution, the ratio of populations of ${S}_{z}\,=\,1/2$ to ${S}_{z}\,=\,-1/2$ electrons, at 298 K, is 0.9566. Therefore, the weighted average of ${S}_{z}$ is

$$\left\langle {S}_{z}\right\rangle=\frac{\frac{1}{2}\times 0.9566+\left(-\frac{1}{2}\right)\times 1}{0.9566+1}=-0.0111$$

Under the conditions where the NMR spectra were taken, and assuming labile Cu^2+^ binding, each guanosine site has a Cu^2+^ occupancy of only 0.01. Therefore, the average hyperfine field felt by the guanosine ligand should be scaled down accordingly, to $\left\langle {S}_{z}\right\rangle=1.11\,\times {10}^{-4}$. As shown in Supplementary Fig. 19c, the NMR peak of the proton moved by about 0.05 ppm, or ${\Delta }_{{{{{{\rm{CS}}}}}}}=20\,{{{{{\rm{Hz}}}}}}$ in a 400 MHz magnet upon adding Cu^2+^. Therefore, one can estimate that

$${A}_{{iso}}=\frac{{\Delta }_{{{{{{\rm{CS}}}}}}}}{\left\langle {S}_{z}\right\rangle }=20{{{{{\rm{\ Hz}}}}}}\div1.11\times {10}^{-4}=0.18{{{{{\rm{\ MHz}}}}}}$$

### Simulation

In order to gain a better understanding of the active site, a comprehensive set of quantum chemical (QM) calculations were performed. These calculations aimed to investigate the structures and interactions of peptide-assembled nanostructures, GMP, Cu^2+^, and O2. Specifically, the crystal structures of Fmoc-K and Fmoc-H, as well as the co-assembly of GMP and Cu^2+^, were analyzed using first-principles calculations. The Vienna ab initio simulation package 5.4.4 and the plane-wave pseudopotential method were employed for all the calculations^77,78^. The Perdew–Burke–Ernzerhof functional^79^ with the generalized gradient approximation was used to account for electronic exchange and correlation effects. The full-potential projector augmented wave method was employed to describe the behavior of the core electrons^80,81^. A plane-wave expansion with an energy cut-off of 400 eV was implemented, ensuring that the force on the relaxed atoms remained below 0.03 eVÅ^−1^. The Monkhorst–Pack scheme was utilized to generate a k-point grid for sampling the Brillouin zone, with a grid size of (8 × 5 × 1) specifically applied to Fmoc-K and Fmoc-H. The peptide conformations obtained from the first-principles calculations were employed in the QM calculations. Additionally, Gaussian 09 software was utilized to optimize the Cu-dimer and triple clusters at the B3LYP/6-31G(d,p) level of theory^82–84^. CrystalDiffract offers robust simulation capabilities for both x-ray and neutron powder diffraction. In this study, the constant-wavelength X-ray simulation type was utilized to generate the XRD patterns. This simulation type was chosen from the available options, which include constant-wavelength X-r.ays, neutrons, energy-dispersive X-rays, and time-of-flight neutrons.

### Statistical analysis

The statistical analysis methods for quantitative data are described in the results sections or figure captions. All values are presented as mean ± standard deviation (s.d.) with the sample size indicated. All statistical tests were two-sided, and the GraphPad Prism 8 software was utilized for the statistical analyses.

### Reporting summary

Further information on research design is available in the Nature Portfolio Reporting Summary linked to this article.

## Supplementary information

Supplementary information

Peer Review File

Reporting Summary

Description of Additional Supplementary files

Supplementary Data 1

Supplementary Data 2

## Data Availability

All data are available within the main text or the Supplementary information. PDB: 1GYC, PDB: 1AOZ.
