# Supplementary material for: A supramolecular metalloenzyme possessing robust oxidase-mimetic catalytic function
Source: Nat Commun. 2023 Jul 7;14:4040. doi: 10.1038/s41467-023-39779-6 (PMC10328989; doi:10.1038/s41467-023-39779-6)
Supplement: Supplementary file 1 — Supplementary information [file 41467_2023_39779_MOESM1_ESM.docx]

Supplementary Information

A Supramolecular Metalloenzyme Possessing Robust Oxidase-Mimetic Catalytic Function

Shichao Xu,^1^ Haifeng Wu,^1^ Siyuan Liu,^1^ Peidong Du,^1^ Hui Wang, ^2*^ Haijun Yang,^3^ Wenjie Xu,^4^ Shuangming Chen,^4^ Li Song,^4^ Jikun Li,^5^ Xinghua Shi,^2^ Zhen-Gang Wang^1*^

^1^State Key Laboratory of Organic-Inorganic Composites, Key Lab of Biomedical Materials of Natural Macromolecules (Beijing University of Chemical Technology, Ministry of Education), Beijing Laboratory of Biomedical Materials, College of Materials Science and Engineering, Beijing University of Chemical Technology, Beijing 100029, China.

^2^Laboratory of Theoretical and Computational Nanoscience, CAS Center for Excellence in Nanoscience, National Center for Nanoscience and Technology, Beijing 100190, China.

^3^Department of Chemistry, Tsinghua University, Beijing 10084, China.

^4^National Synchrotron Radiation Laboratory, CAS Center for Excellence in Nanoscience, University of Science and Technology of China, Hefei,230029 China.

^5^Institute of Chemistry, Chinese Academy of Sciences (ICCAS), Beijing, 100190, China.

* Address correspondence to: wangh@nanoctr.cn, wangzg@mail.buct.edu.cn

**
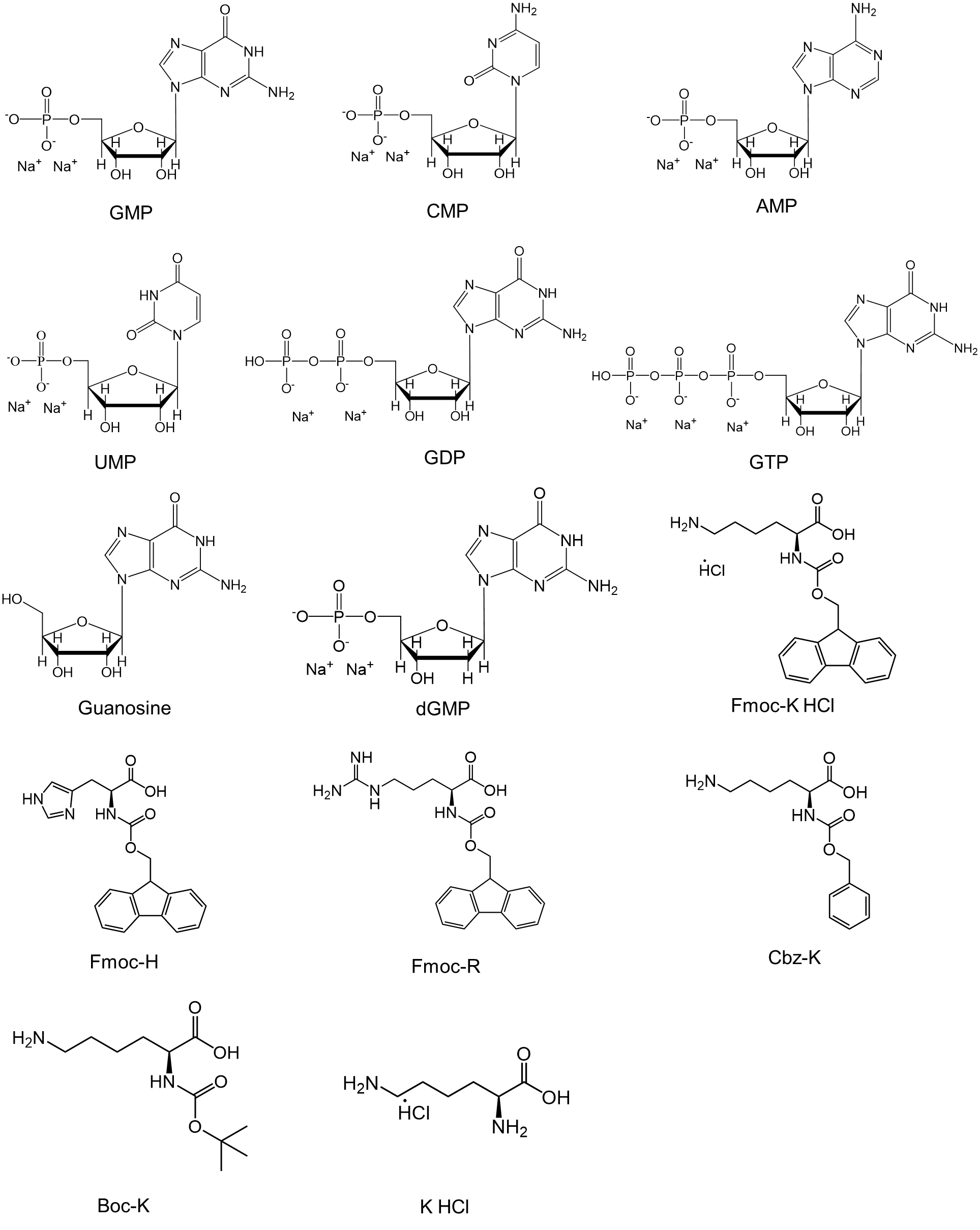
Supplementary Figure 1.** Molecular structures of the various nucleotides and amino acids used in this work.


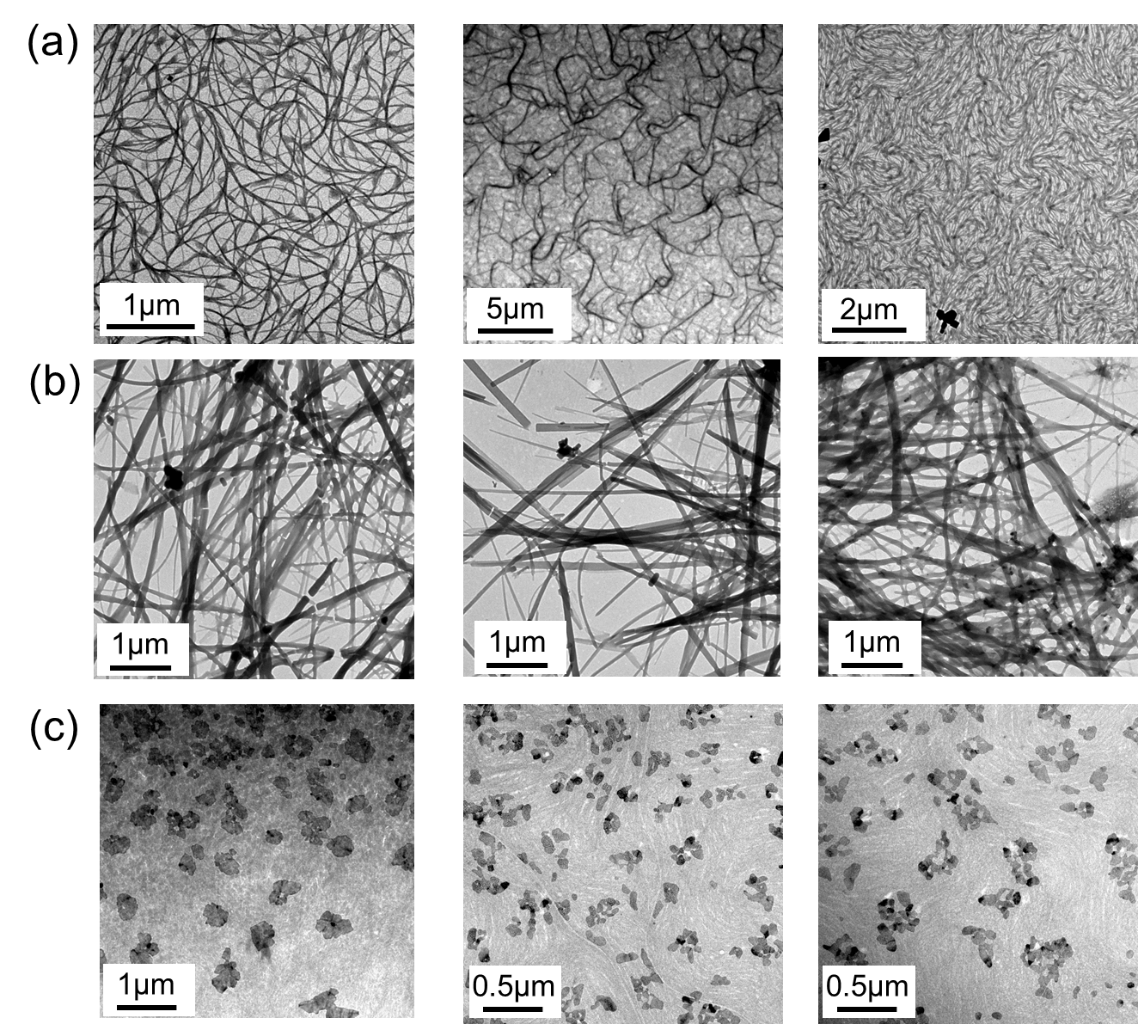
**Supplementary Figure 2.** TEM images of (A) GMP/Cu^2+^, (B) Fmoc-K/Cu^2+^ and (C) Fmoc-K/GMP/Cu^2+^. [Cu^2+^] = 5 μM, [Fmoc-K] = 5 mM, [GMP] = 10 mM.


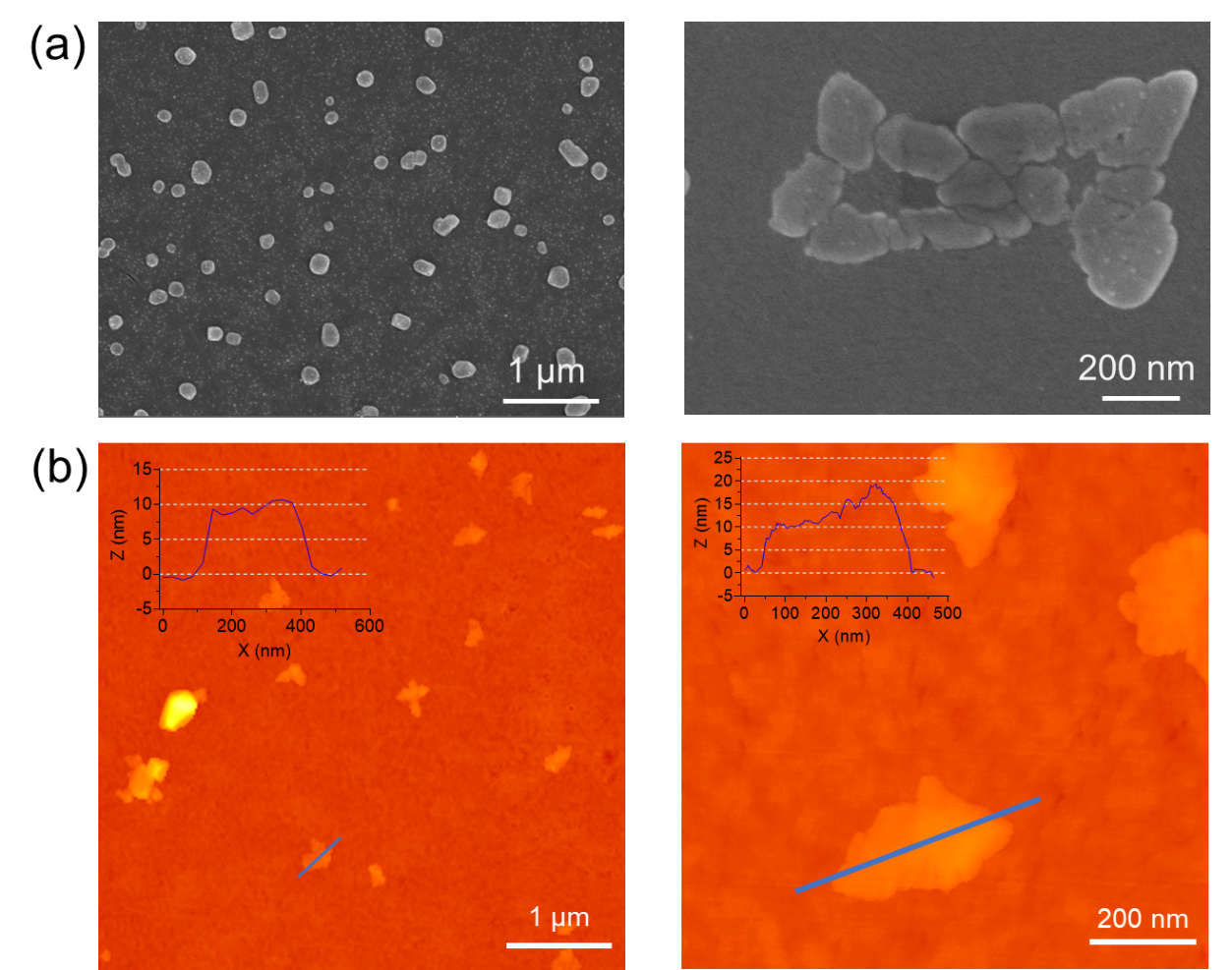
**Supplementary Figure 3.** SEM (a) and AFM images (b) of Fmoc-K/GMP/Cu^2+^. [Cu^2+^] = 5 μM, [Fmoc-K] = 5 mM, [GMP] = 10 mM.

**
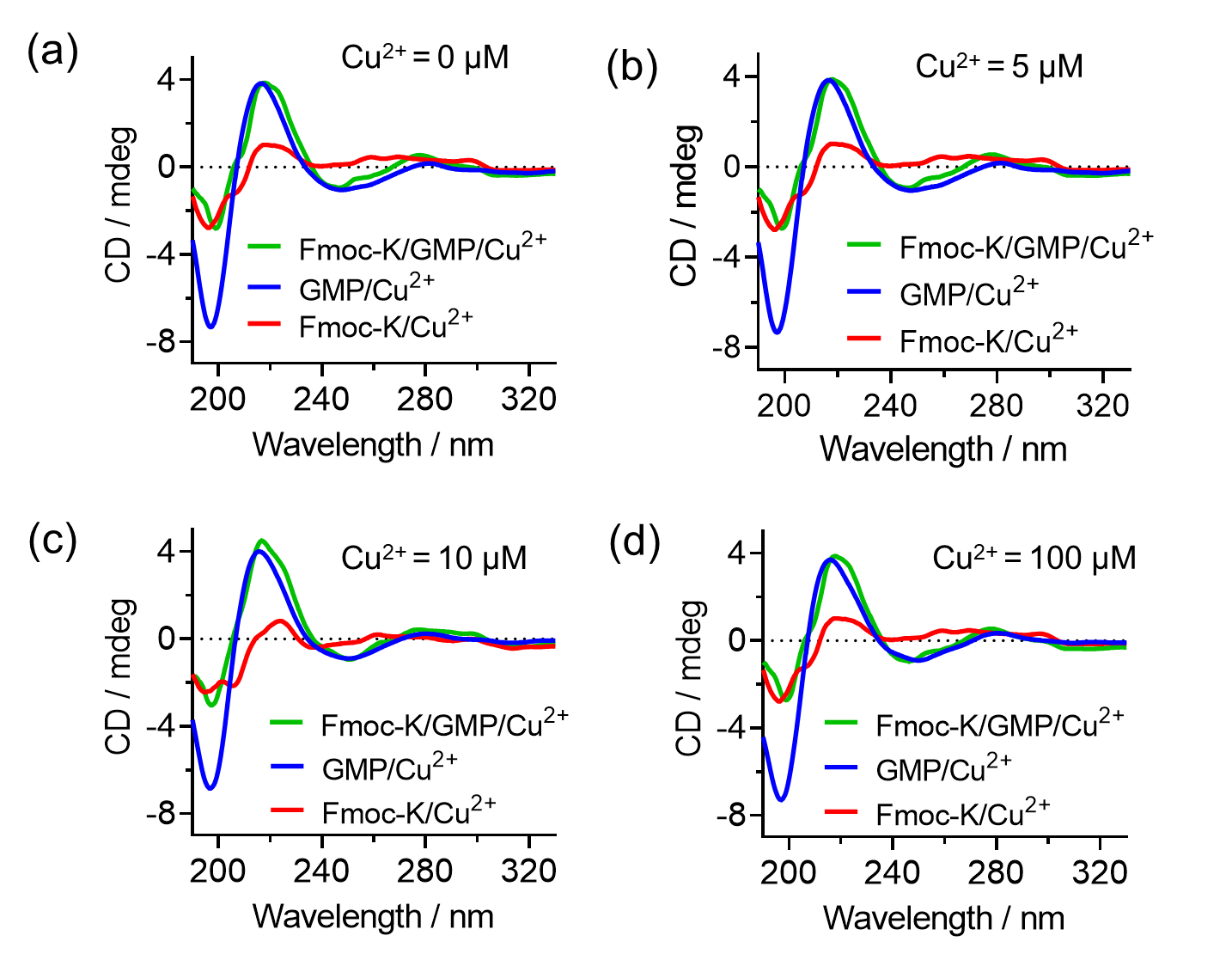
Supplementary Figure 4.** CD spectra of Fmoc-K/GMP/Cu^2+^ complex at different Cu^2+^ concentrations. [Fmoc-K] = 5 mM, [GMP] = 10 mM.

**
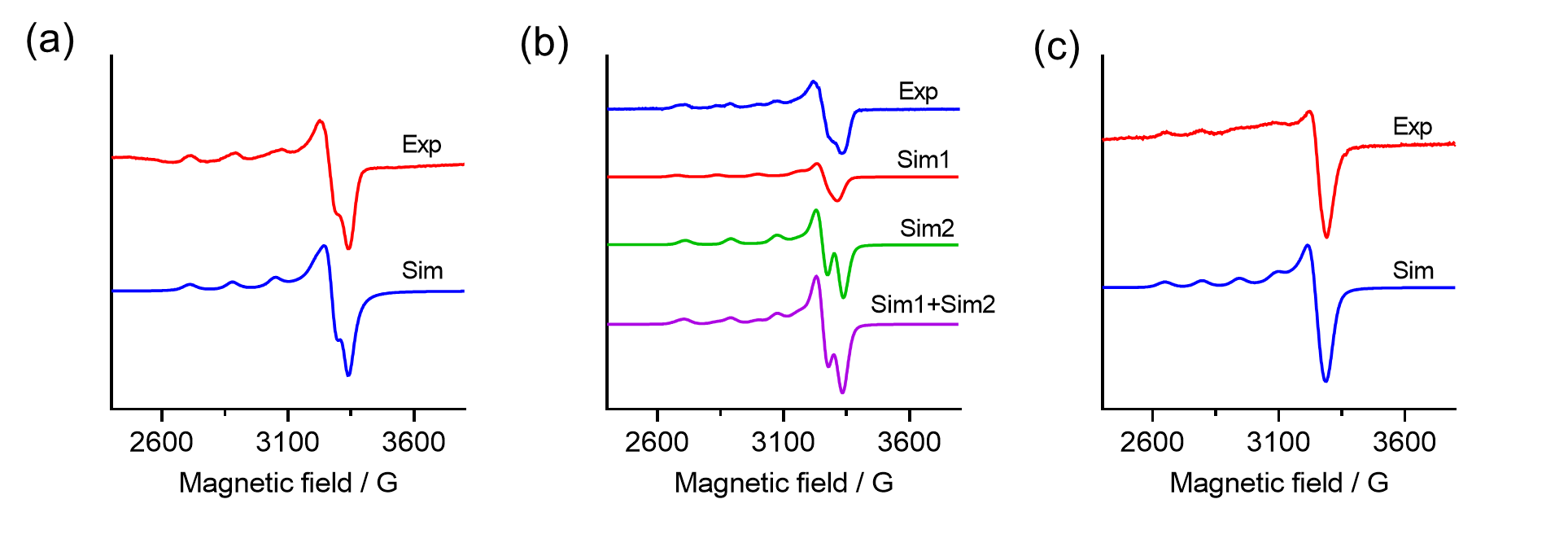
Supplementary Figure 5.** Experimental and simulated EPR spectra of (a) Fmoc-K/GMP/Cu^2+^, (b) Fmoc-K /Cu^2+^ and (c) GMP/Cu^2+^. [Cu^2+^] = 100 μM, [Fmoc-K] = 5 mM, [GMP] = 10 mM.

**
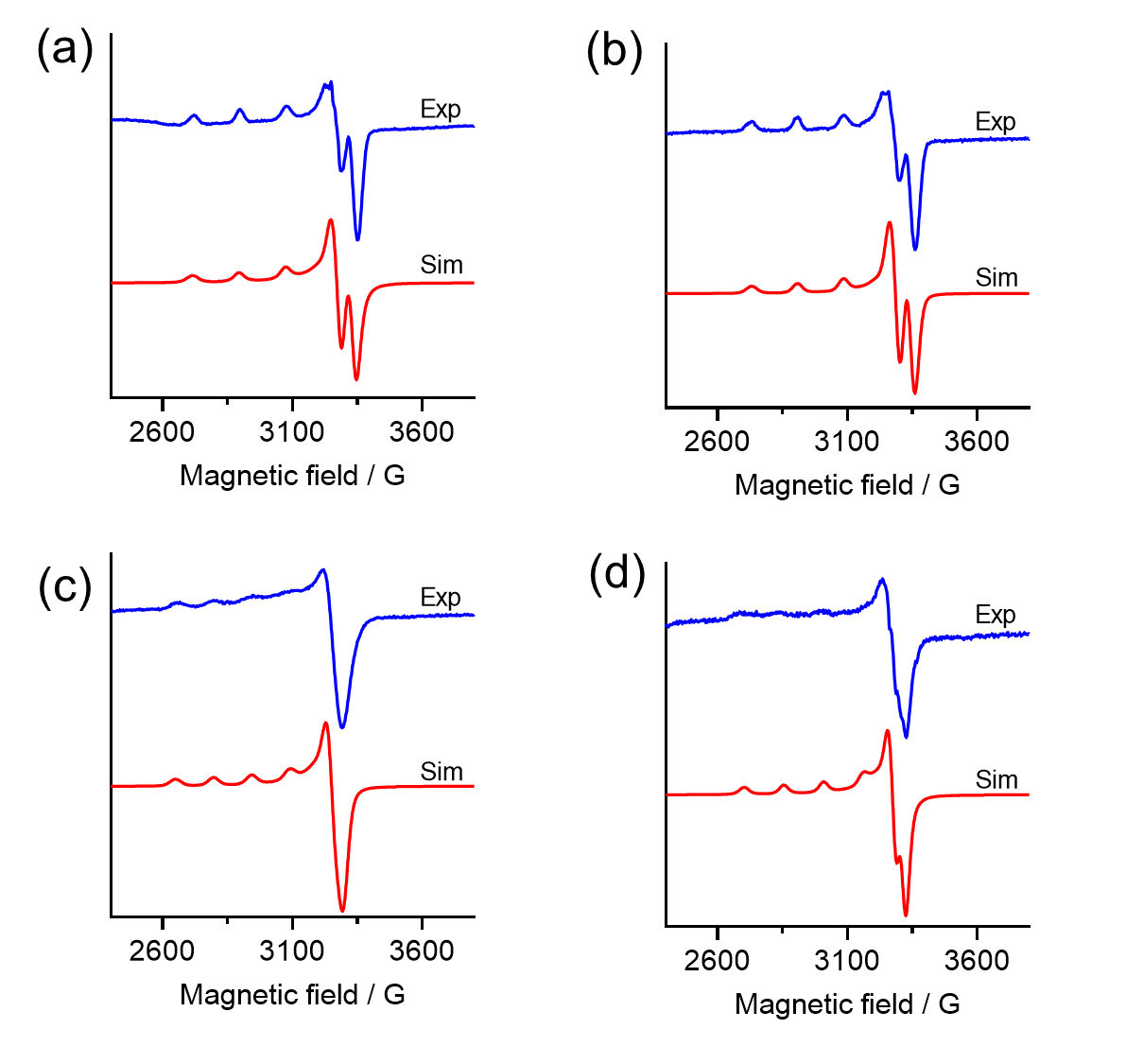
Supplementary Figure 6.** Experimental and simulated EPR spectra of (a) Boc-K/GMP/Cu^2+^, (b) Boc-K/Cu^2+^, (c) Cbz-K/GMP/Cu^2+^ and (d) Cbz-K/Cu^2+^. [Cu^2+^] = 100 μM, [Boc-K] = 5 mM, [Cbz-K] = 5 mM, [GMP] = 10 mM.

**
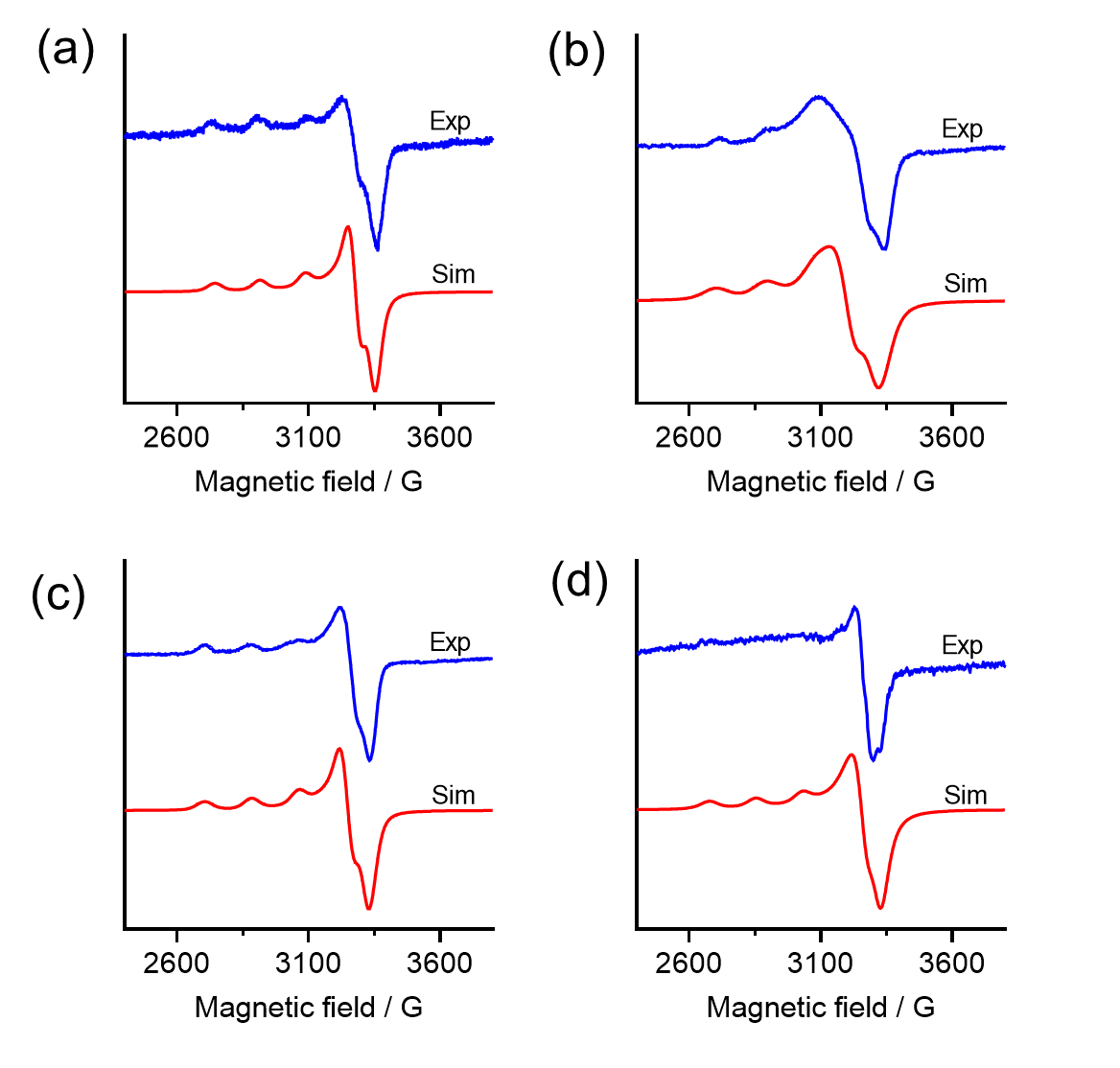
Supplementary Figure 7.** Experimental and simulated EPR spectra of (a) Fmoc-H/GMP/Cu^2+^, (b) Fmoc-H/Cu^2+^, (c) Fmoc-R/GMP/Cu^2+^ and (d) Fmoc-R/Cu^2+^. [Cu^2+^] = 100 μM, [Fmoc-H] = 1 mM, [Fmoc-R] = 1 mM, [GMP] = 2 mM.

**Supplementary Figure 8.** Fluorescence spectra of Fmoc-K/GMP/Cu^2+^, Fmoc-H/GMP/Cu^2+^, and Fmoc-R/GMP/Cu^2+^ complexes. λ_ex_=320 nm. [Fmoc-K] = 1 mM, [Fmoc-H] = 1 mM, [Fmoc-R] = 1 mM, [GMP] = 2 mM. [Cu^2+^] = 5 μM.

**
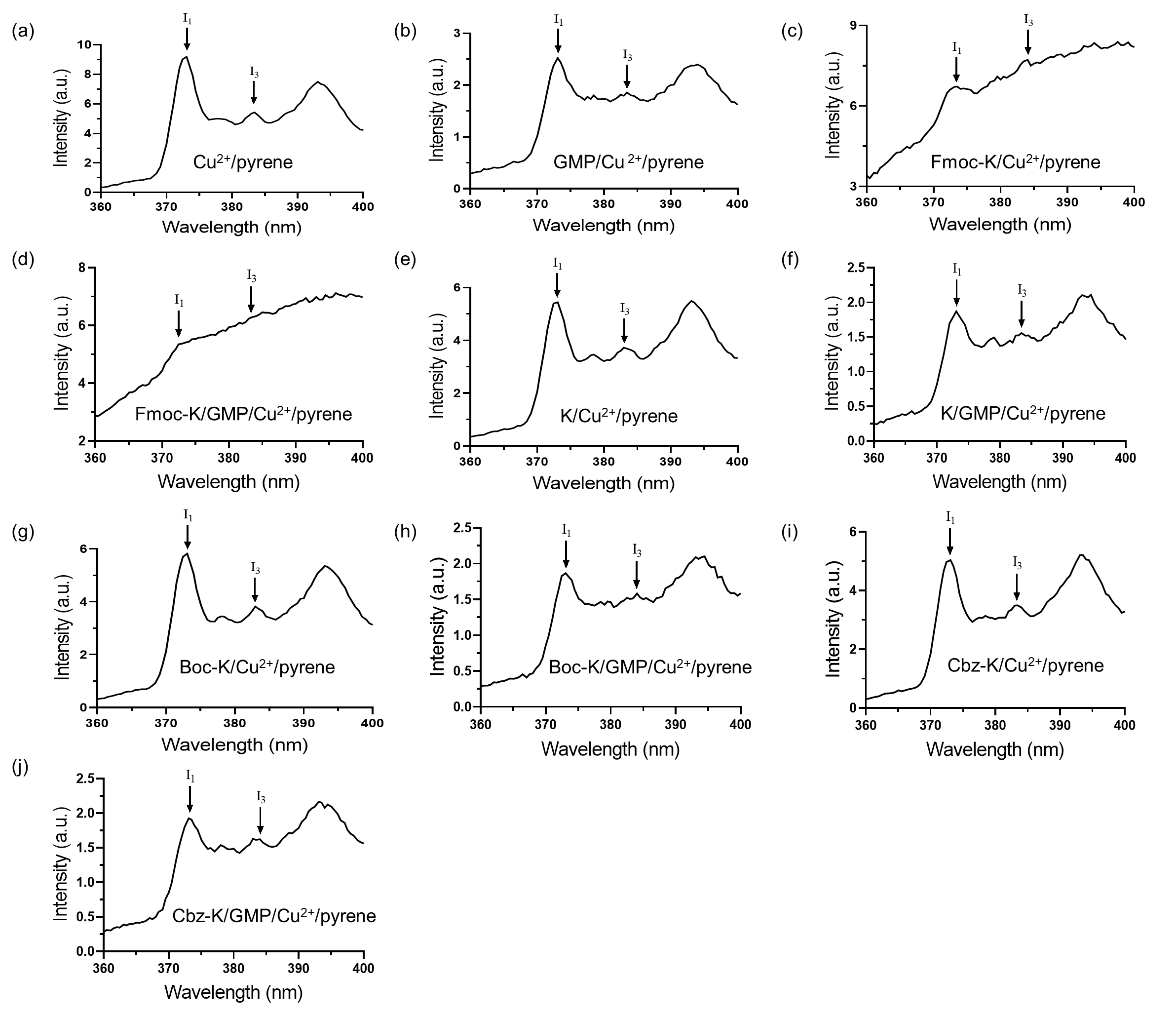
Supplementary Figure 9.** Fluorescence spectra of the mixtures of pyrene and Cu^2+^-contained complexes. λ_ex_=334 nm. [Pyrene] = 5 μM, [Cu^2+^] = 5 μM. [GMP] = 10 mM, [Fmoc-K] = 5 mM, [K] = 5 mM, [Boc-K] = 5 mM, [Cbz-K] = 5 mM.

**Supplementary Figure 10.** Cu K-edge XANES data and Fourier-transformed EXAFS spectra (inset) of (a) Boc-K/GMP/Cu^2+^, (b) Cbz-K/GMP/Cu^2+^, (c) Fmoc-R/GMP/Cu^2+^ and (d) Fmoc-H/GMP/Cu^2+^. [Cu^2+^] = 200 μM, [Boc-K] = 5 mM, [Cbz-K] = 5 mM, [Fmoc-R] = 1 mM, [Fmoc-H] = 5 mM, [GMP] = 10 mM.


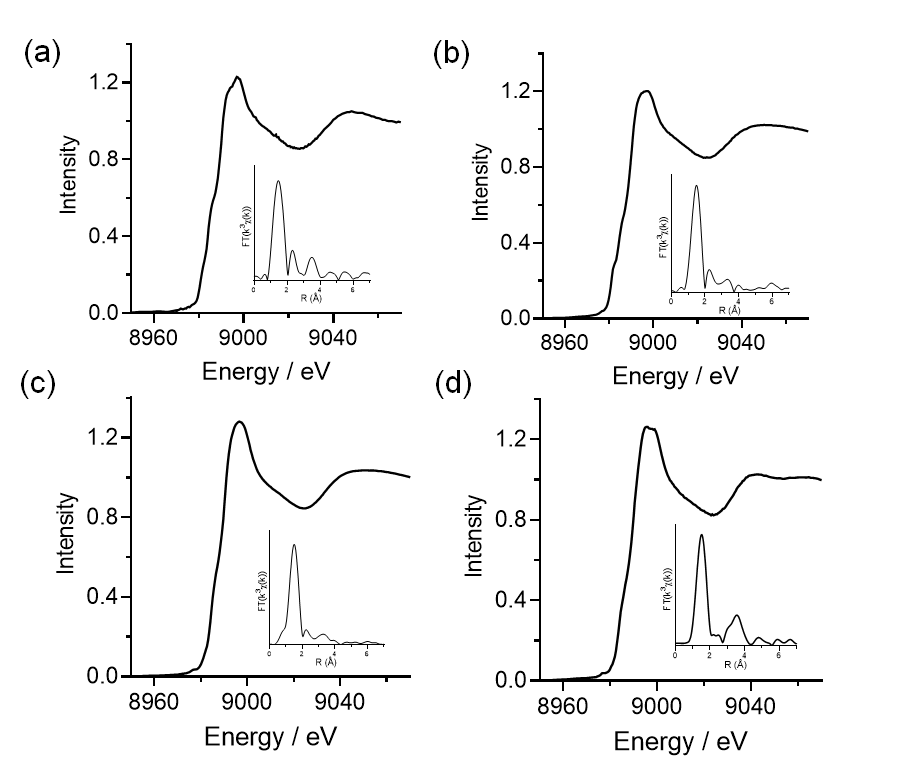


**
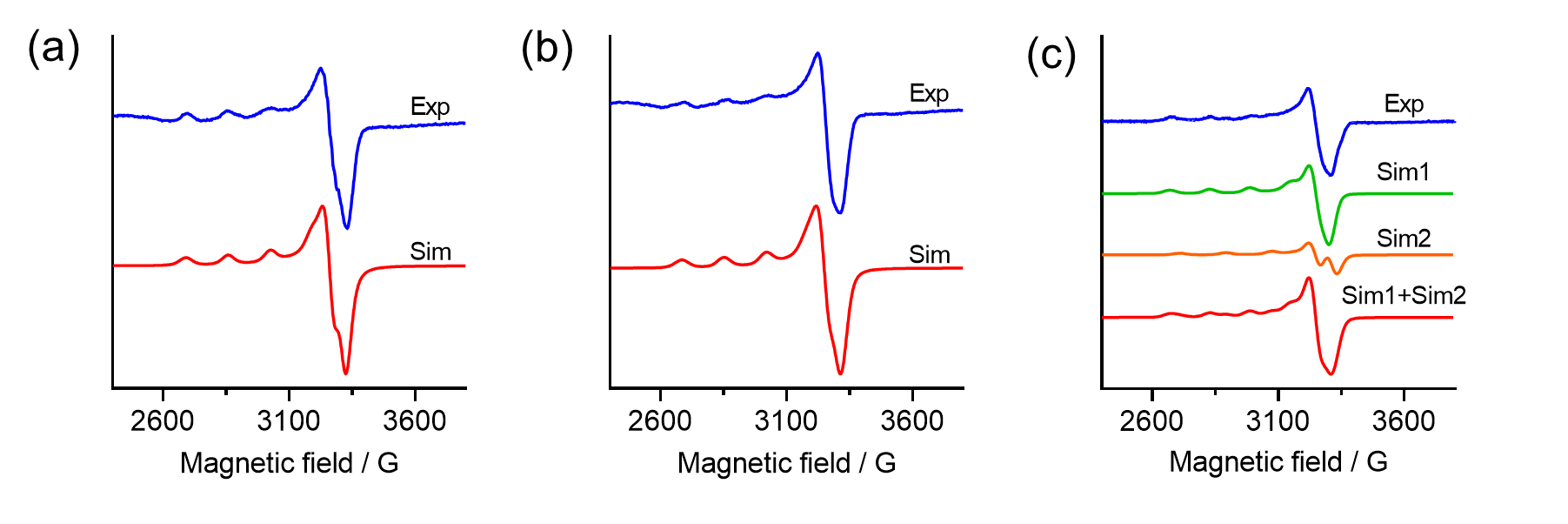
Supplementary Figure 11.** Experimental and simulated EPR spectra of (a) Fmoc-K/CMP/Cu^2+^, (b) Fmoc-K/AMP/Cu^2+^ and (c) Fmoc-K/UMP/Cu^2+^. [Cu^2+^] = 100 μM, [Fmoc-K] = 5 mM, [CMP] = 10 mM, [AMP] = 10 mM, [UMP] = 10 mM.

**
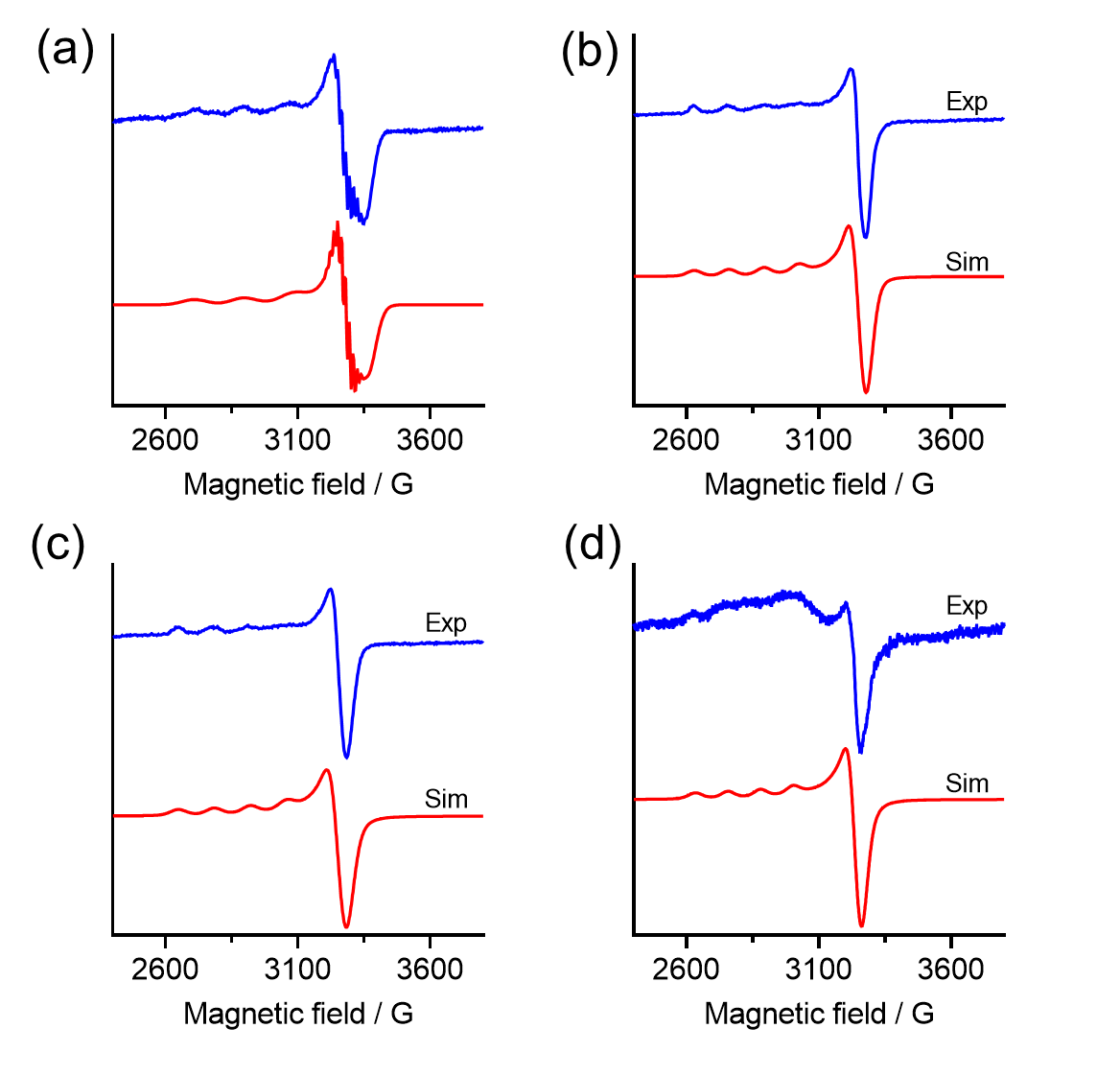
Supplementary Figure 12.** Experimental and simulated EPR spectra of (a) CMP/Cu^2+^, (b) UMP/Cu^2+^, (c) AMP/Cu^2+^and (d) CuSO_4_. [Cu^2+^] = 100 μM, [CMP] = 10 mM, [AMP] = 10 mM, [UMP] = 10 mM.

**Supplementary Figure 13.** Experimental and simulated EPR spectra of (a) Fmoc-K/GTP/Cu^2+^ and (b) Fmoc-K/GDP/Cu^2+^. [Cu^2+^] = 100 μM, [Fmoc-K] = 5 mM, [GTP] = 10 mM, [GDP] = 10 mM.


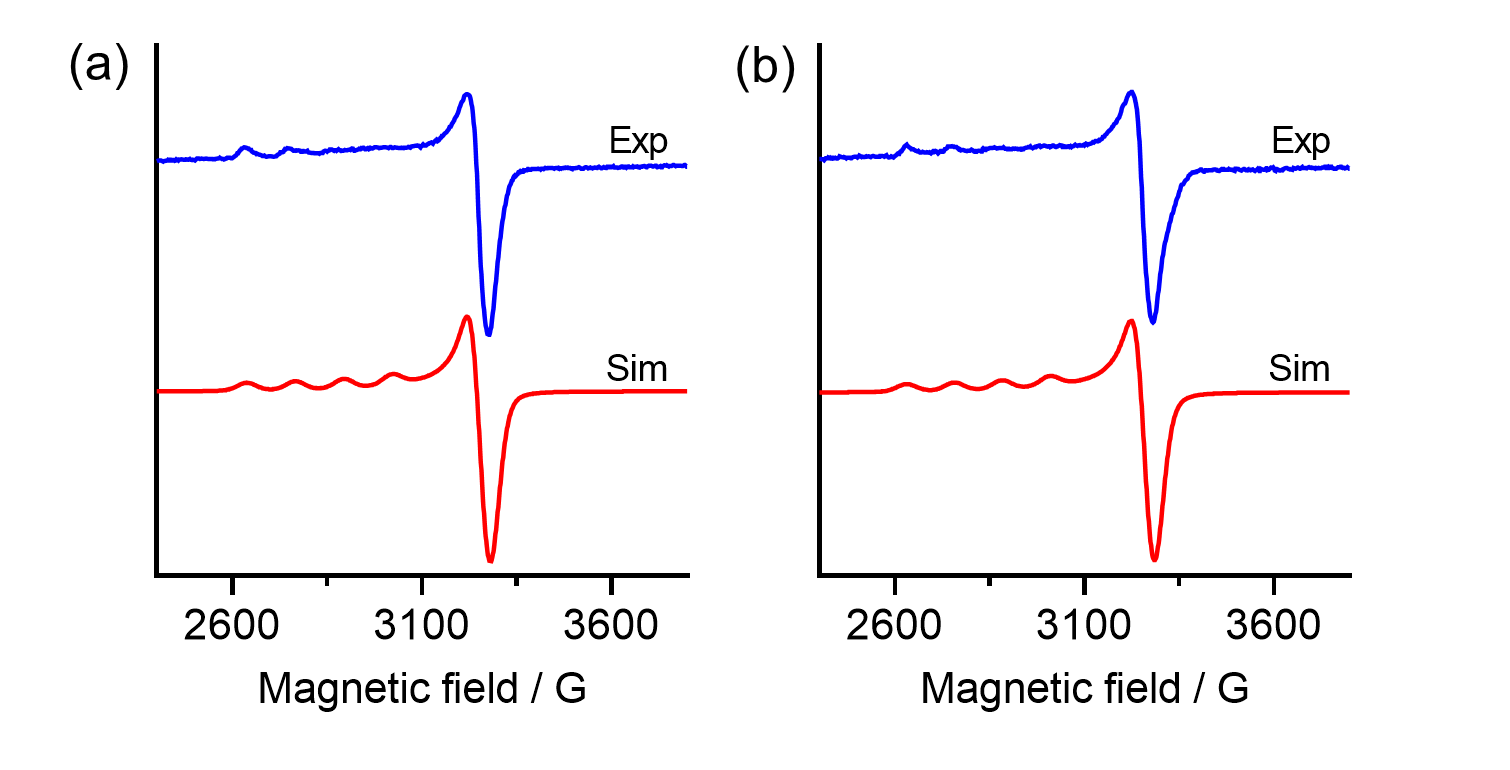


**Supplementary Figure 14.** Experimental and simulated EPR spectrum of Fmoc-K/guanosine/Cu^2+^. [Cu^2+^] = 100 μM, [Fmoc-K] = 1 mM, [guanosine] = 2 mM.

**Supplementary Figure 15.** K-edge X-ray absorption spectra and Fourier transforms of the k^3^-weighted EXAFS (inset) modulations for Fmoc-K/UMP/Cu^2+^. [Cu^2+^] = 200 μM, [Fmoc-K] = 5 mM, [UMP] = 10 mM.

**
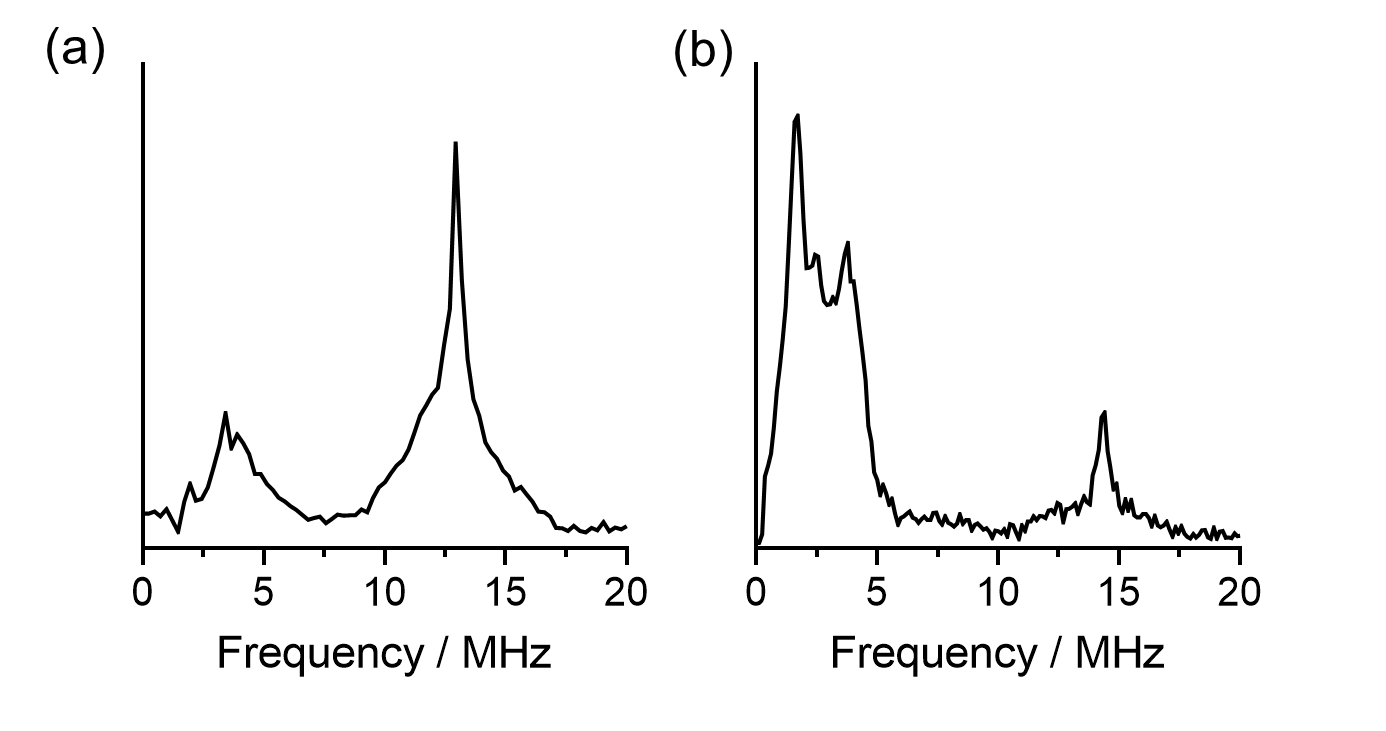
Supplementary Figure 16.** The three-pulse ESEEM spectra of (a) Fmoc-K/Cu^2+^ and (b) GMP/Cu^2+^. [Cu^2+^] = 250 μM, [Fmoc-K] = 5 mM, [GMP] = 10 mM.

**
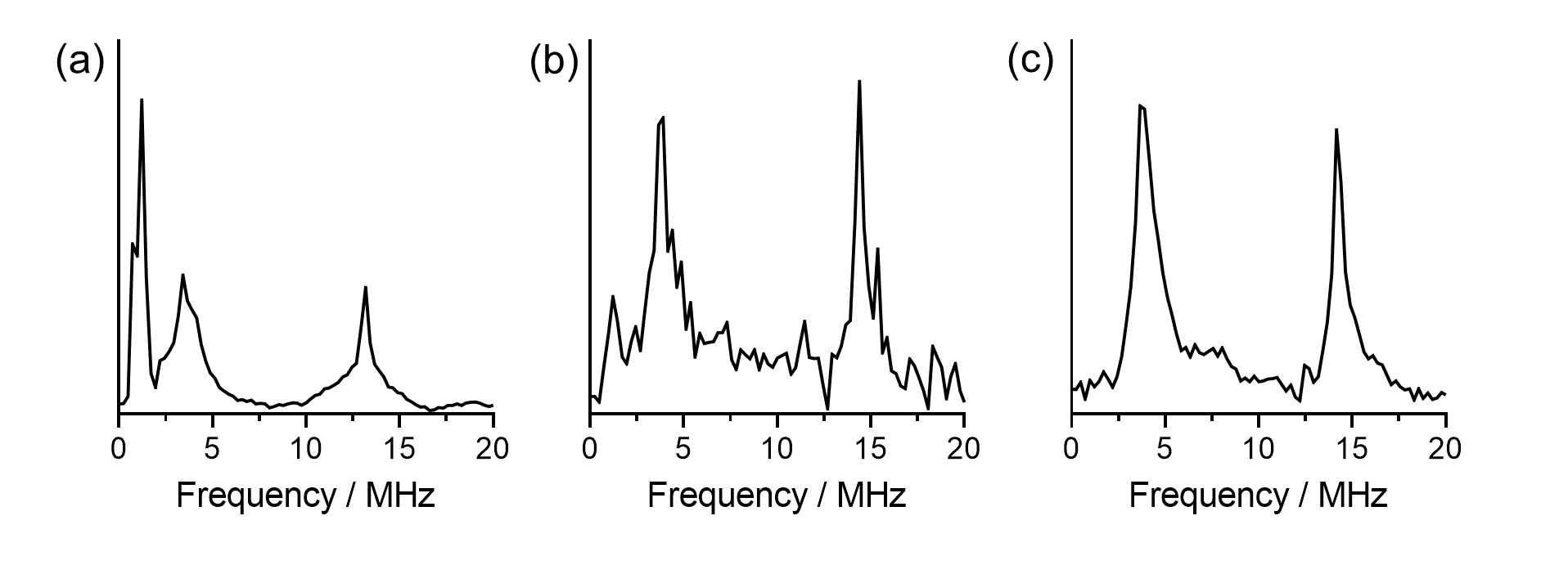
Supplementary Figure 17.** The three-pulse ESEEM spectra of (a) Fmoc-H/Cu^2+^, (b) Fmoc-R/Cu^2+^ and (c) Cbz-K/Cu^2+^. [Cu^2+^] = 250 μM, [Fmoc-H] = 5 mM, [Fmoc-R] = 5 mM, [Cbz-K] = 5 mM.

**
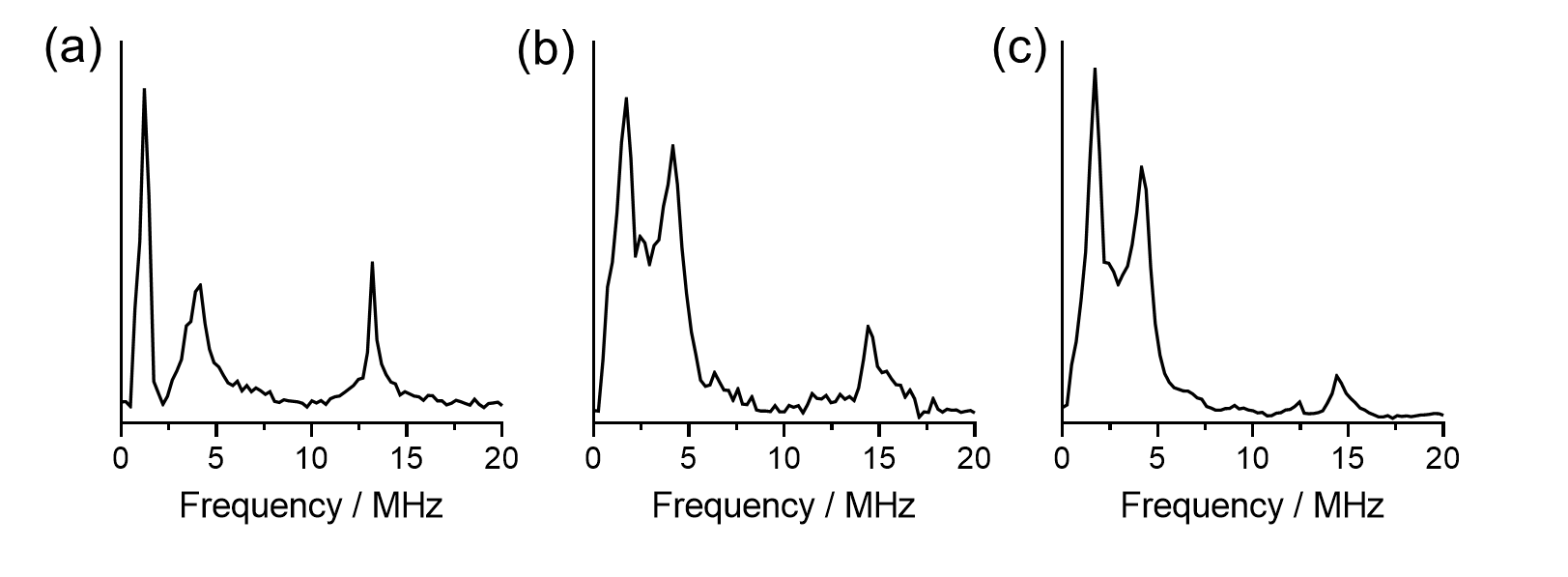
Supplementary Figure 18.** The three-pulse ESEEM spectra of (a) Fmoc-H/GMP/Cu^2+^, (b) Fmoc-R/GMP/Cu^2+^, (c) Cbz-K/GMP/Cu^2+^. [Cu^2+^] = 250 μM, [Fmoc-H] = 1 mM, [Fmoc-R] = 5 mM, [Cbz-K] = 5 mM, [GMP] = 10 mM.

**Supplementary Figure 19.** ^1^H-NMR of (a) Guanosine, Guanosine/Cu^2+^. [Guanosine] = 10 mM, [Cu^2+^] = 50 μM. (b) Fmoc-K, Fmoc-K/Cu^2+^. [Fmoc-K] = 10 mM, [Cu^2+^] = 50 μM. (c) Fmoc-K/Guanosine, Fmoc-K/Guanosine/Cu^2+^. [Fmoc-K] = 5 mM, [Guanosine] = 5 mM, [Cu^2+^] = 50 μM.


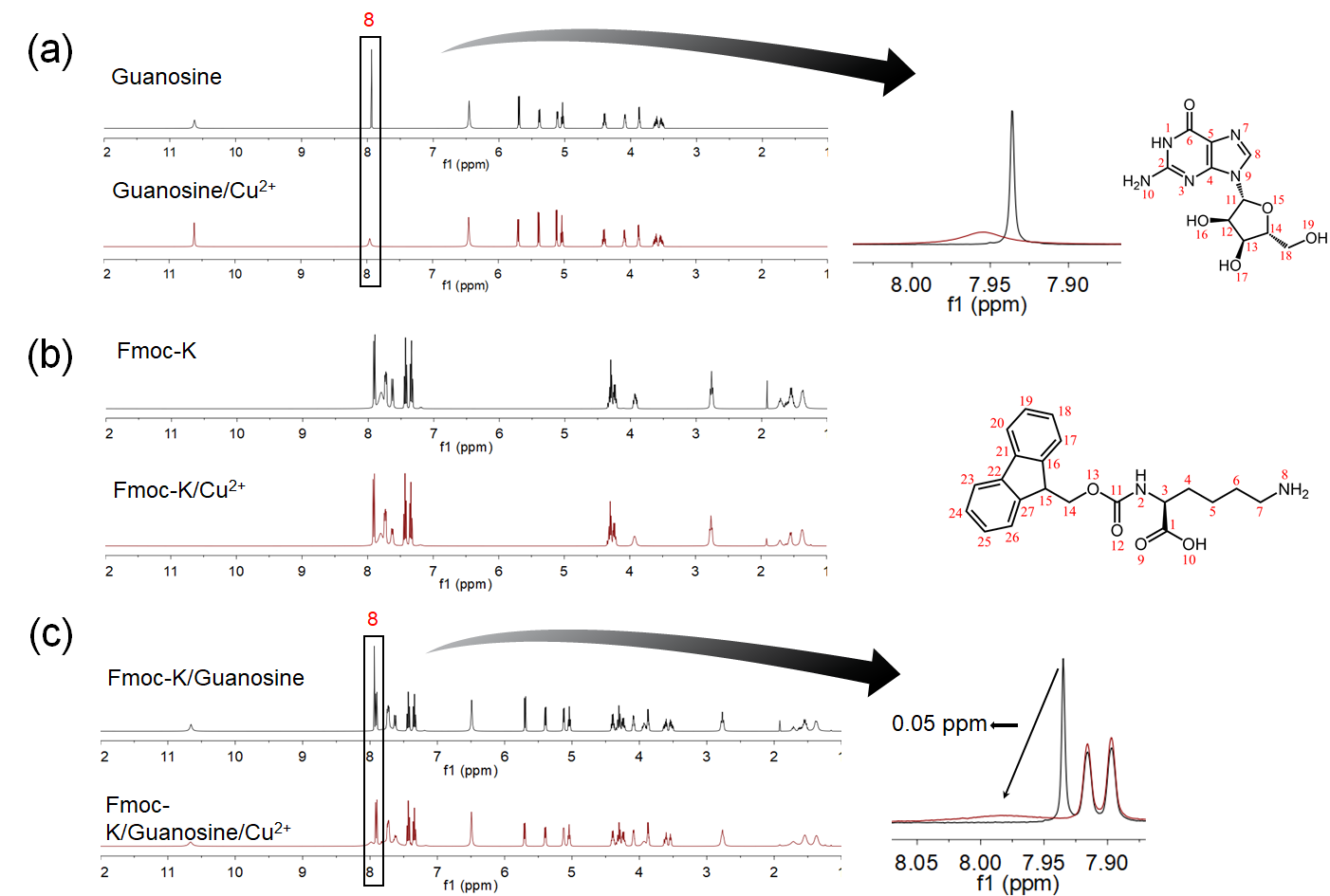


**
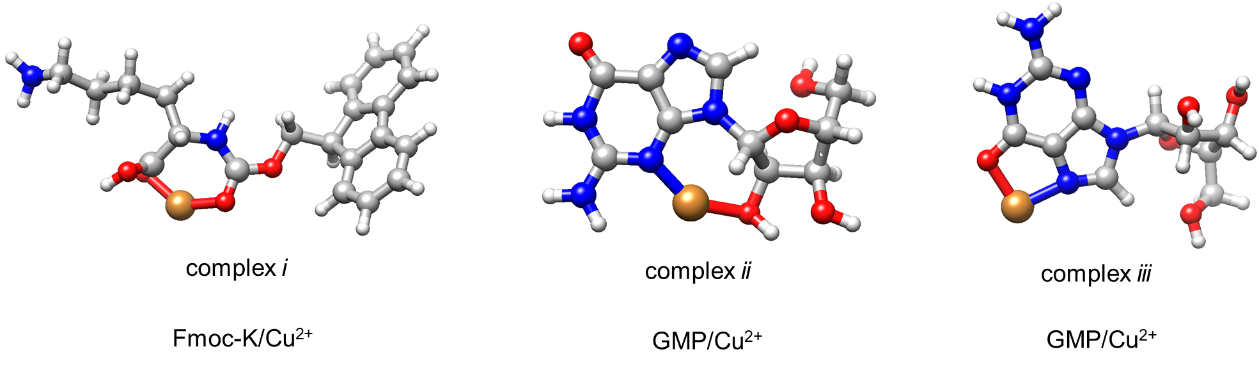
Supplementary Figure 20.** Theoretical models of Fmoc-K/Cu^2+^ and GMP/Cu^2+^ complex. N, O, C, H, Cu atoms are indicated in blue, red, dark grey, light grey and orange.

**Supplementary Figure 21.** Experimental and simulated EPR spectrum of Fmoc-K/dGMP/Cu^2+^. [Cu^2+^] = 100 μM, [Fmoc-K] = 5 mM, [dGMP] = 10 mM.

**Supplementary Figure 22.** The k^3^-weighted EXAFS modulations for Fmoc-K/dGMP/Cu^2+^. [Cu^2+^] = 200 μM, [Fmoc-K] = 5 mM, [dGMP] = 10 mM.

**
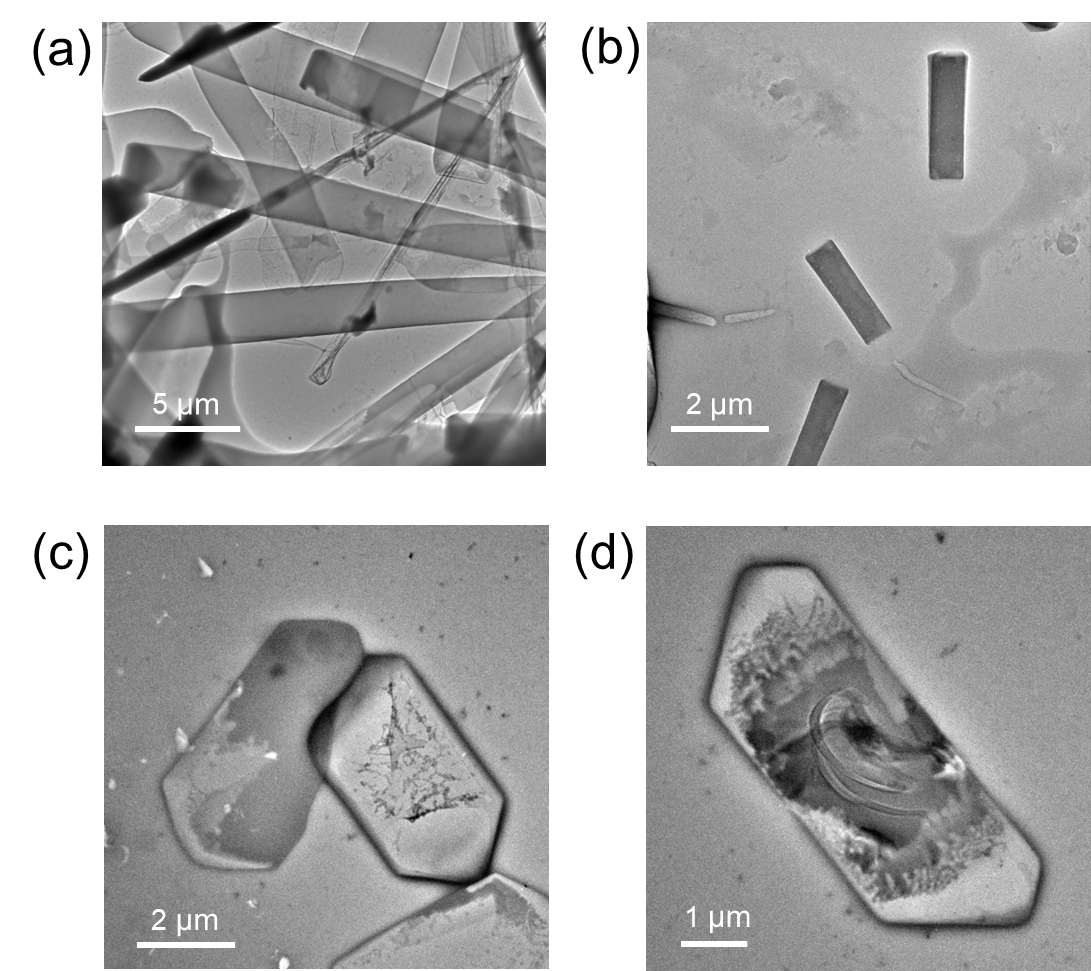
Supplementary Figure 23.** TEM images of Fmoc-K crystal.

**
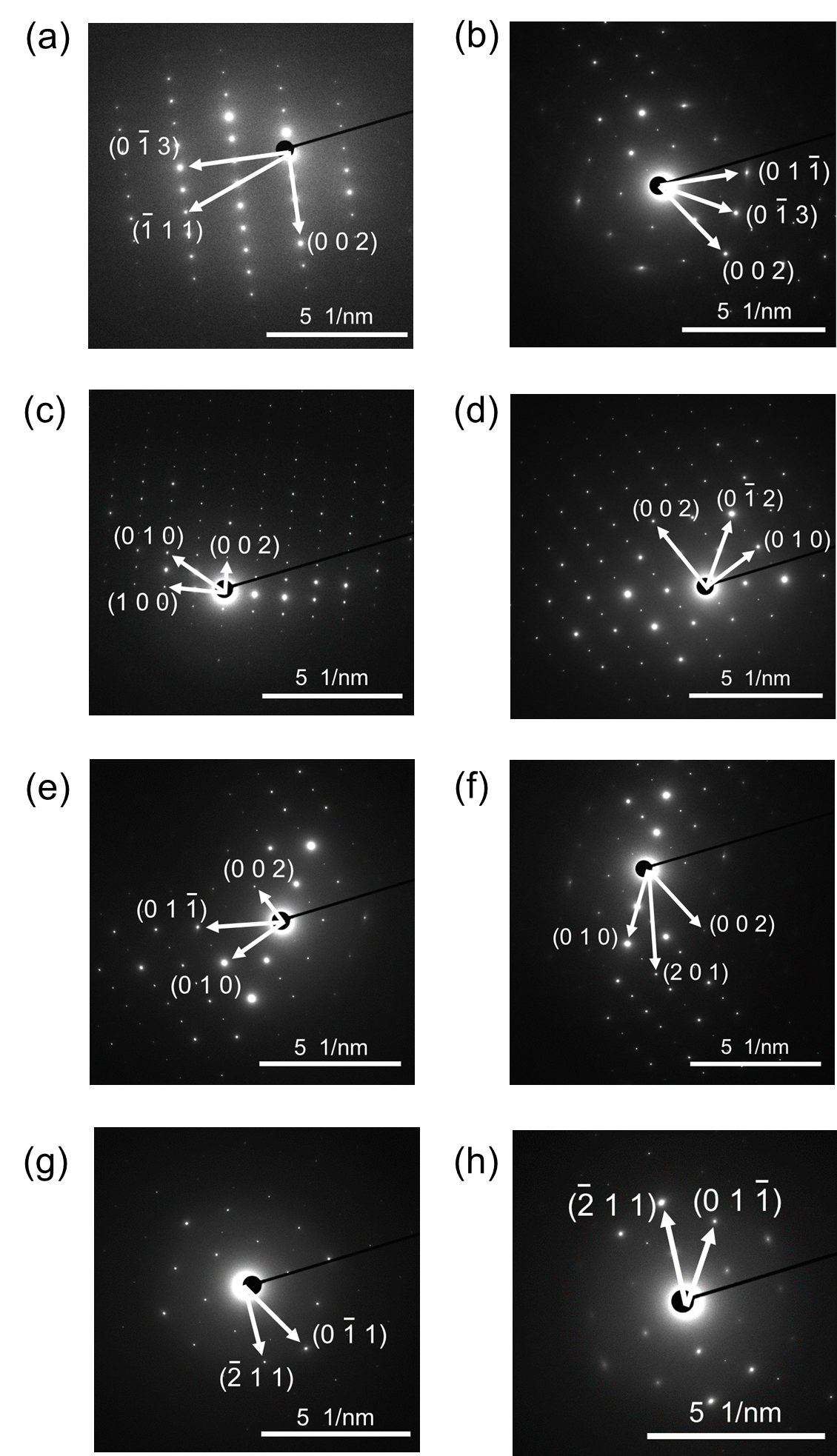
Supplementary Figure 24.** SAED patterns of Fmoc-K crystals. It is noteworthy that the facets marked were estimated according to the simulation.

**Supplementary Figure 25.** The XRD pattern simulated from the crystal of Fmoc-K.

**Supplementary Figure 26.** Low temperature (120K) powder XRD patterns of the Fmoc-K crystals.

**
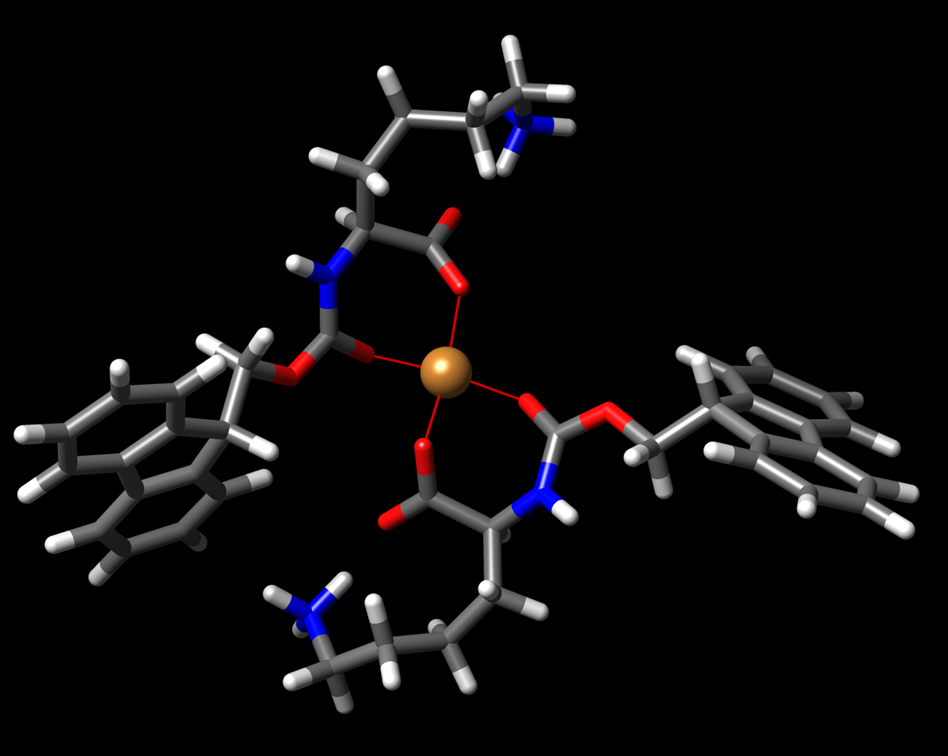
Supplementary Figure 27.** The model of Cu^2+^ coordinated to Fmoc-K molecules along facet (0 0 1). N, O, C, H, Cu atoms are indicated in blue, red, dim grey, light grey and orange.

**
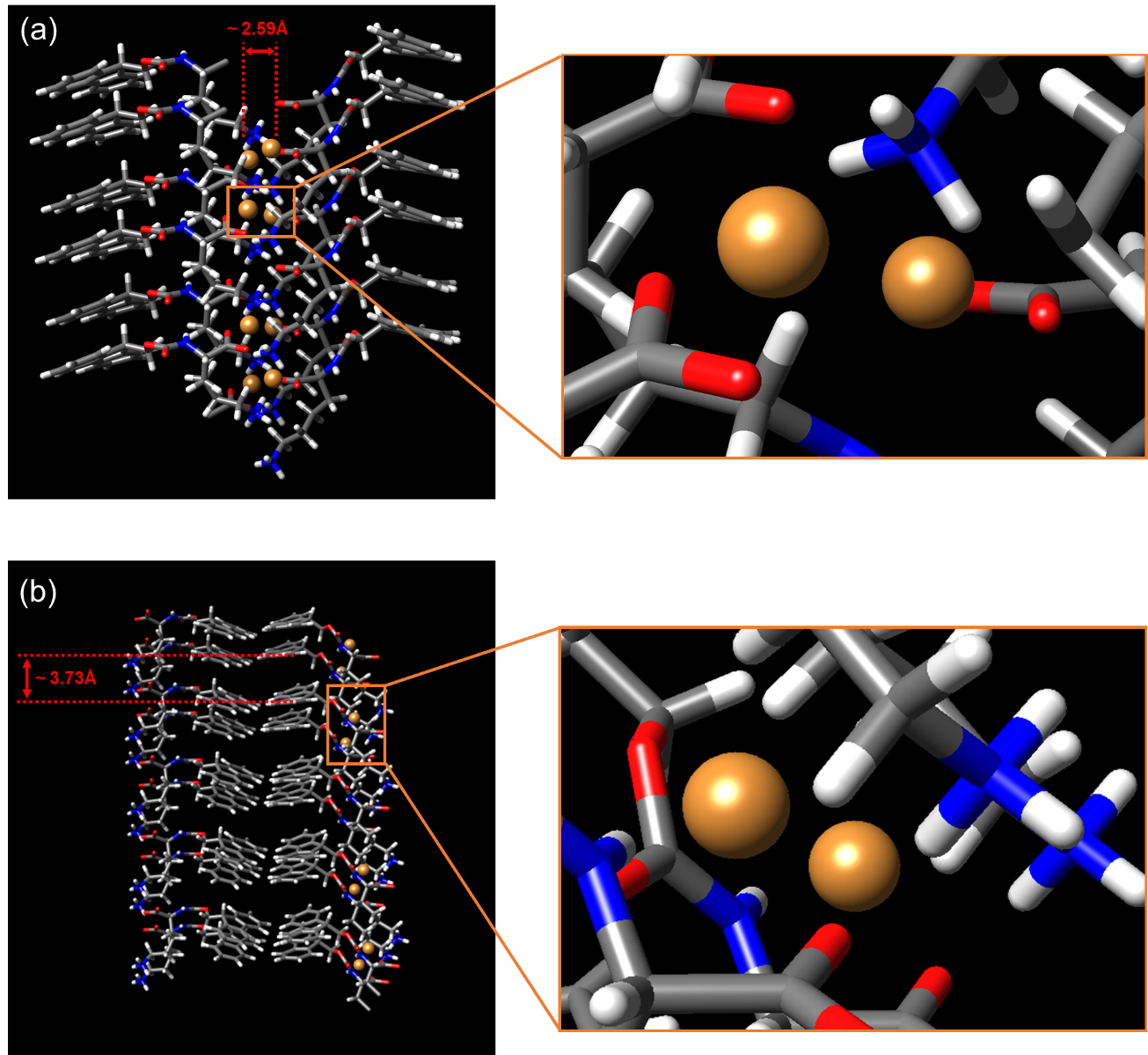
Supplementary Figure 28.** The model of copper sites in Fmoc-K along facet (a) (0 0 1) and (b) (1 0 0). N, O, C, H, Cu atoms are indicated in blue, red, dim grey, light grey and orange.

**
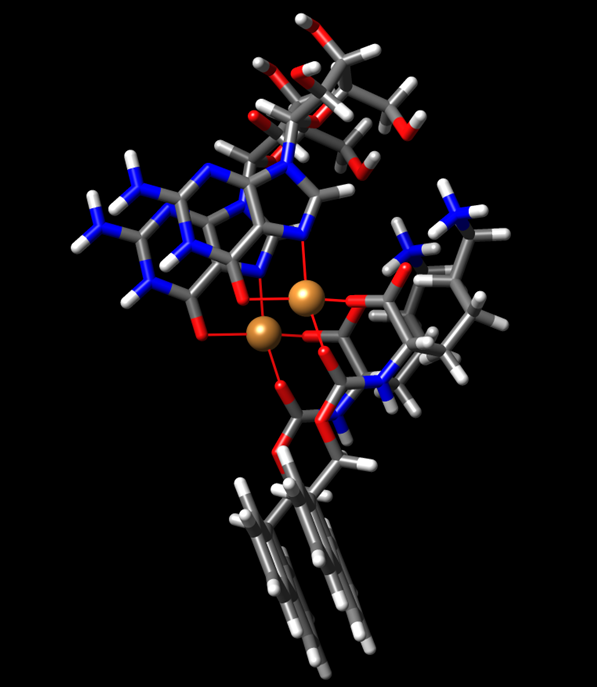
Supplementary Figure 29.** Theoretical coordination model of Cu^2+^ to Fmoc-K and GMP. N, O, C, H, Cu atoms are indicated in blue, red, dark grey, dim grey and orange.

**Supplementary Figure 30.** Fluorescence spectra of Fmoc-K/GMP and Fmoc-K/GMP/Cu^2+^. [Fmoc-K] = 5 mM, [GMP] = 10 mM, [Cu^2+^] = 5 μM.

**
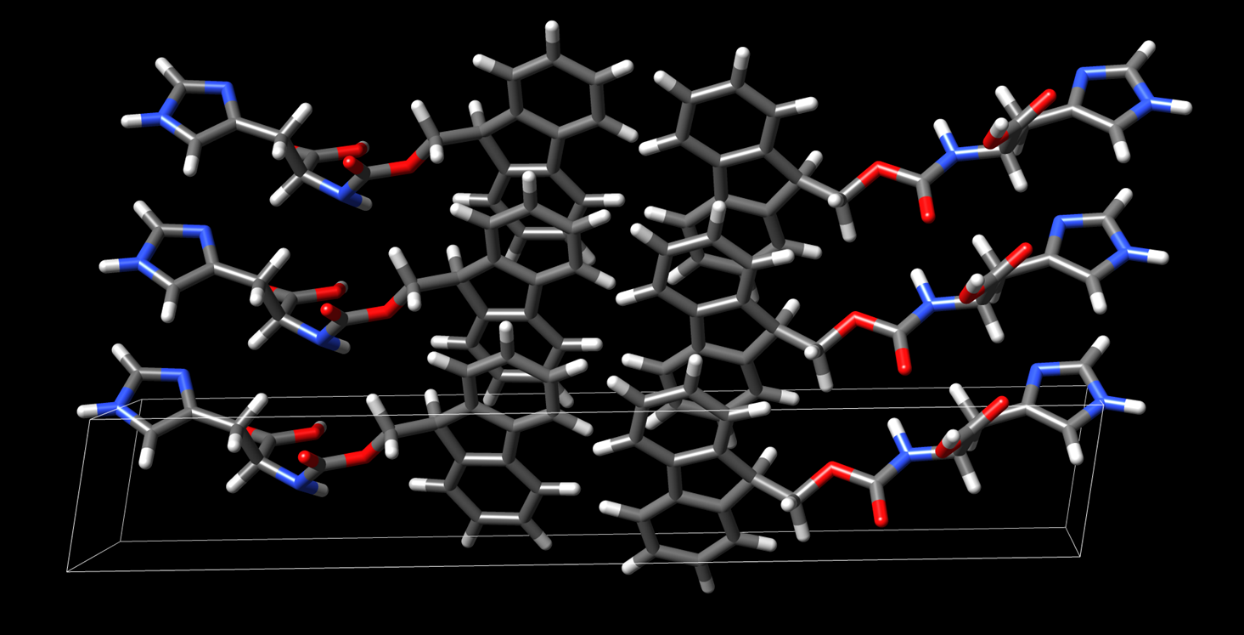
Supplementary Figure 31.** Theoretical model of Fmoc-H crystal. N, O, C, H, Cu atoms are indicated in blue, red, dim grey, dim grey and orange.

**
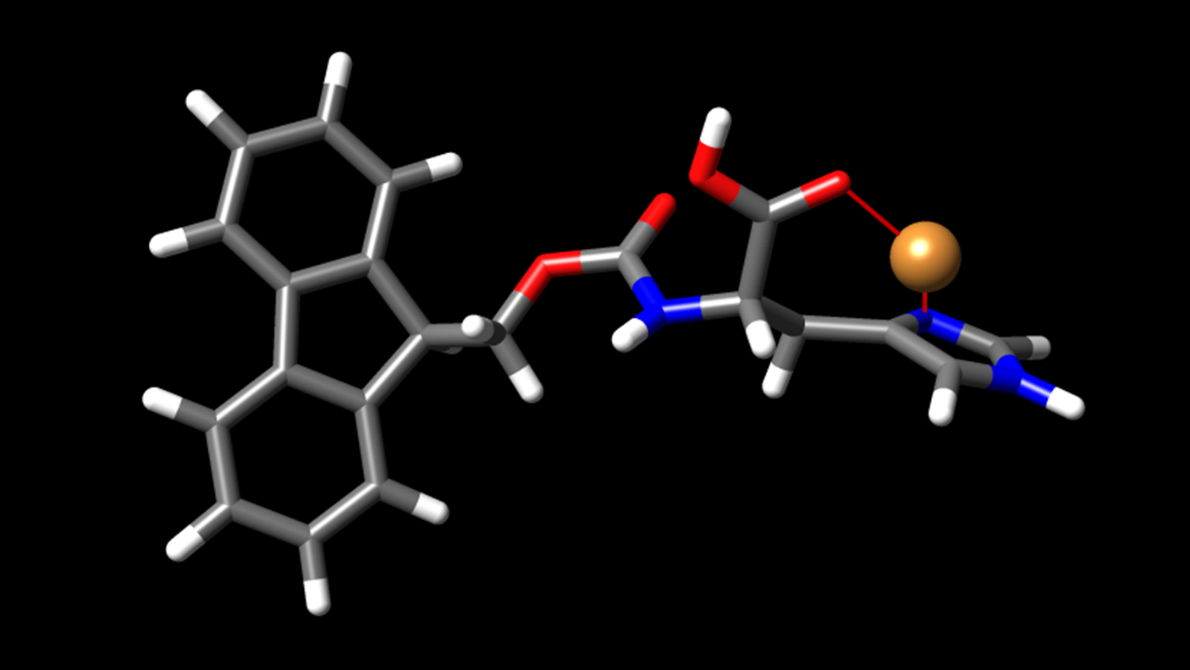
Supplementary Figure 32.** Theoretical models of Fmoc-H/Cu^2+^ complex. N, O, C, H, Cu atoms are indicated in blue, red, dim grey, light grey and orange.

**
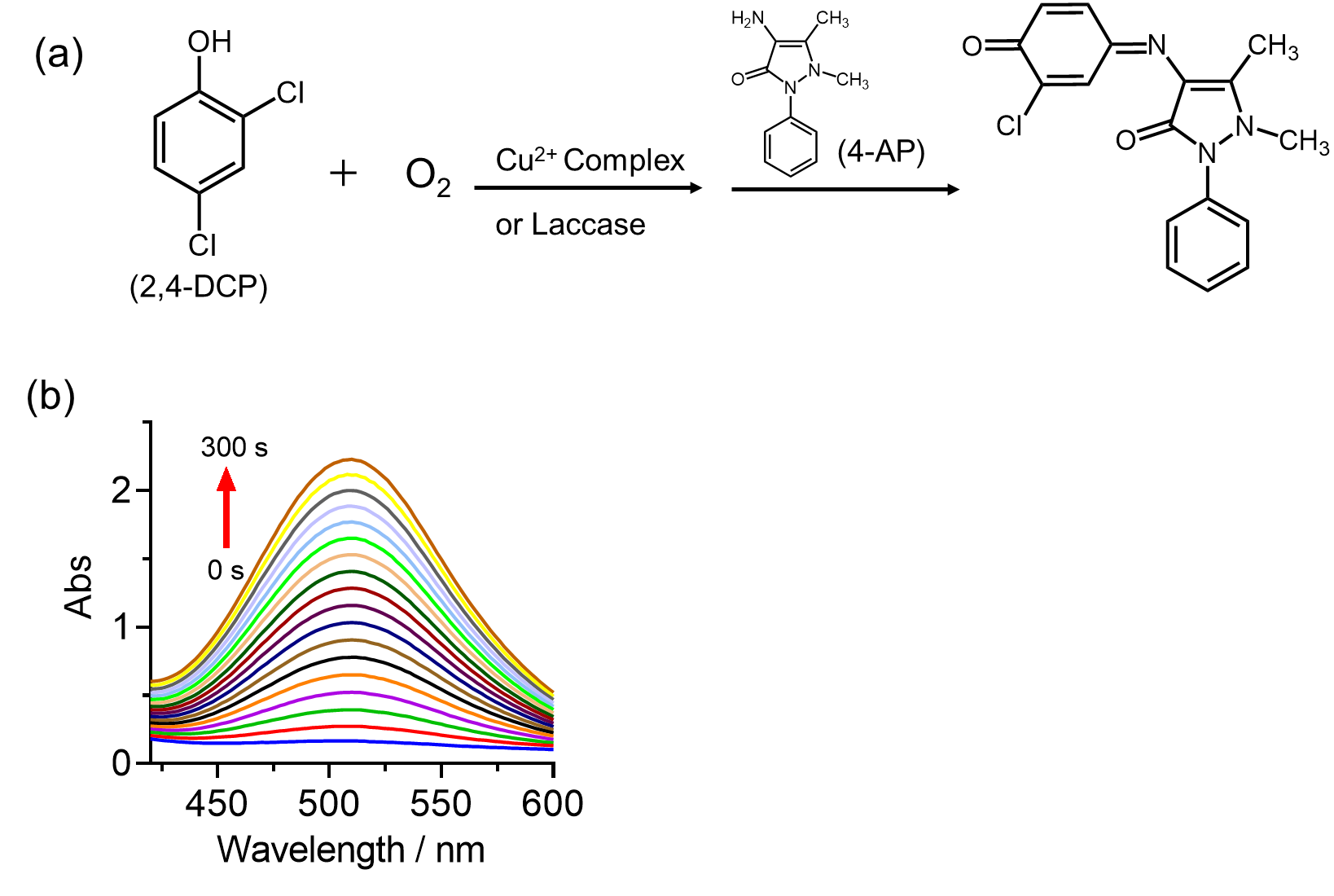
Supplementary Figure 33.** (a) Schematic catalyzed oxidation of 2,4-DCP by the oxidase or oxidase-mimetic catalyst, and colorimetric reaction between the oxidized 2,4-DCP and 4-AP to yield red adduct with maximum absorbance at 510 nm. (b) Time-resolved UV-vis spectral changes of the mixtures of 2,4-DCP, 4-AP and Fmoc-K/GMP/Cu^2+^ in the atmosphere. [Fmoc-K] = 5 mM, [GMP] = 10 mM, [Cu^2+^] = 5 μM. [2,4-DCP] = 1 mM, [4-AP] = 1 mM.

**
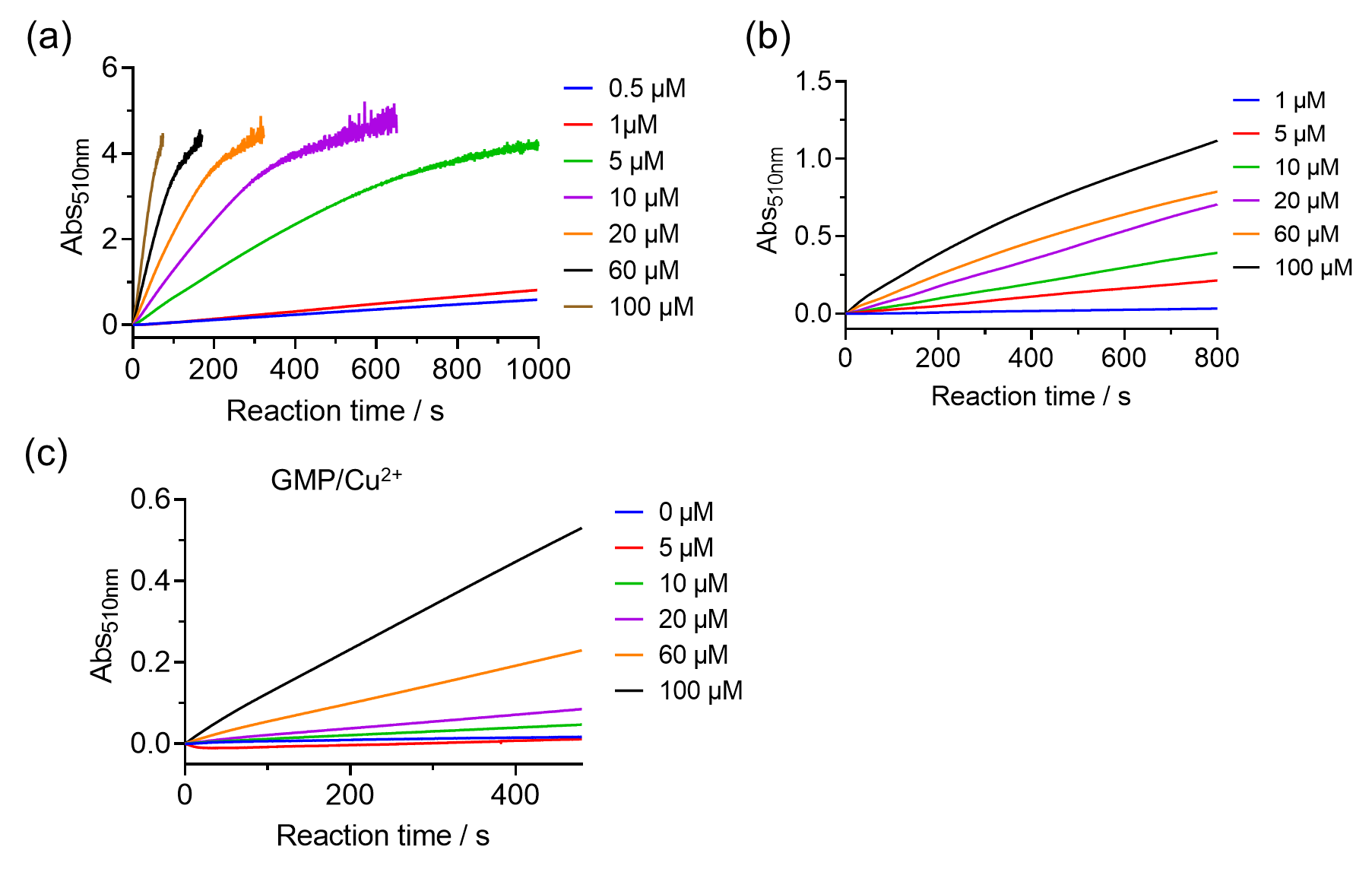
Supplementary Figure 34.** Time-dependent absorbance changes at 510 nm for the catalyzed oxidations of 2,4-DCP in the presence of 4-AP at various Cu^2+^ concentrations by (a) Fmoc-K/GMP/Cu^2+^, (b) Fmoc-K/Cu^2+^, and (c) GMP/Cu^2+^. [Fmoc-K] = 5 mM, [GMP] = 10 mM, [2,4-DCP] = 1 mM, [4-AP] = 1 mM.

**
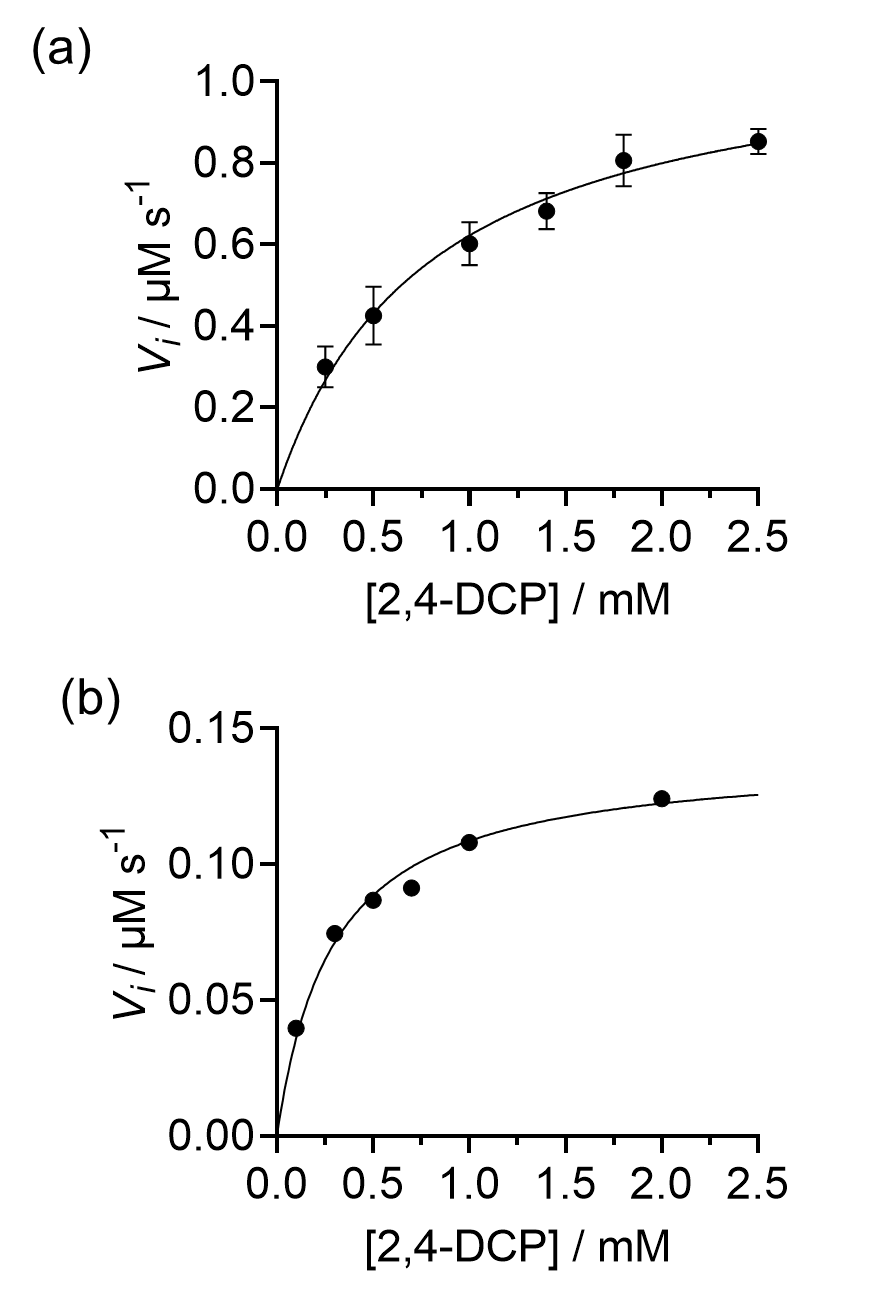
Supplementary Figure 35.** The depencence of the initial catalytic velocities of (a) Fmoc-K/GMP/Cu^2+^ and (b) laccase on the concentrations of 2,4-DCP as the reducing substrate. [Fmoc-K] = 5 mM, [GMP]= 10 mM, [Cu^2+^] = 5 μM. [Laccase] =5 μM. The data in (a) and (b) are presented as the mean ± s.d., with the error bars representing the s.d. and n = 3 independent experiments.

**
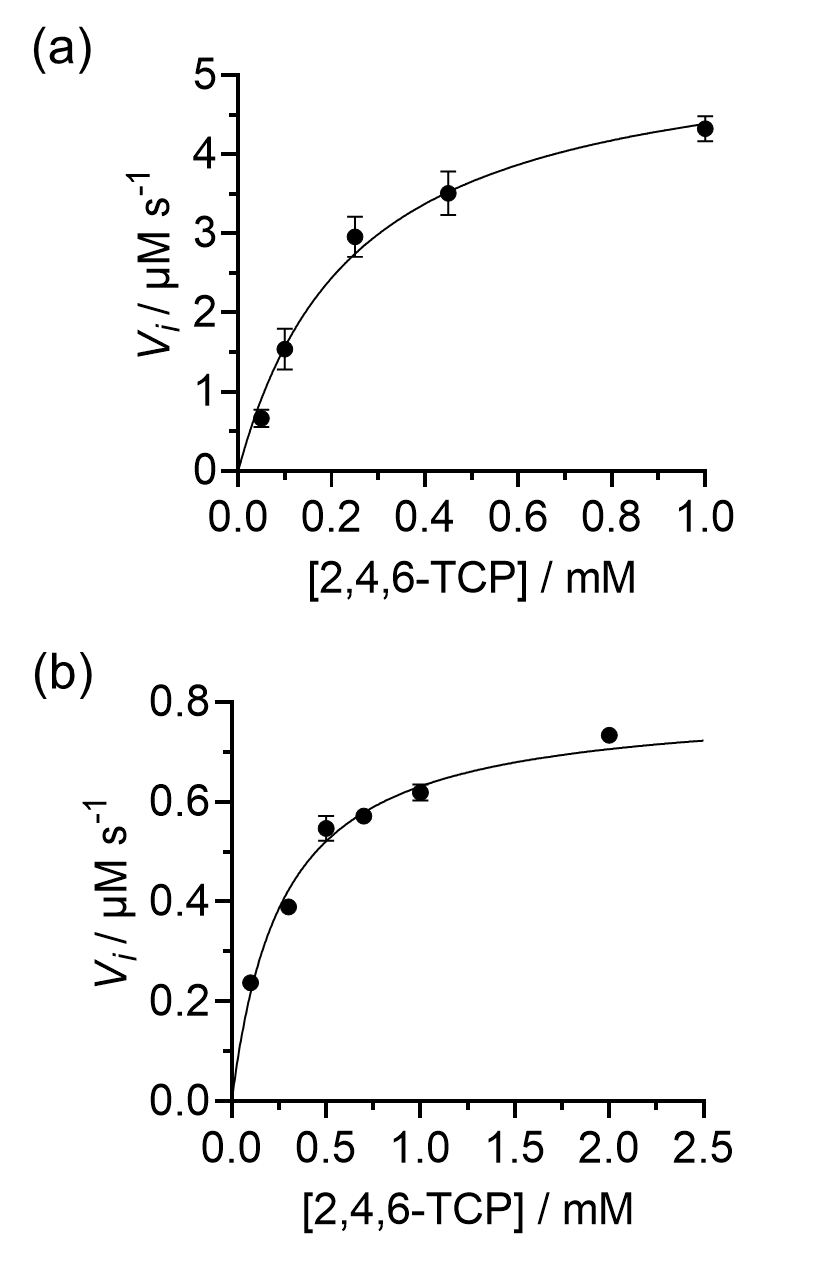
Supplementary Figure 36.** The initial catalytic velocities of (a) Fmoc-K/GMP/Cu^2+^ and (b) laccase at different concentrations of 2,4,6-TCP as the reducing substrate. [Fmoc-K] = 5 mM, [GMP]= 10 mM, [Cu^2+^] = 5 μM. [Laccase] =5 μM. The data in (a) and (b) are presented as the mean ± s.d., with the error bars representing the s.d. and n = 3 independent experiments.

**
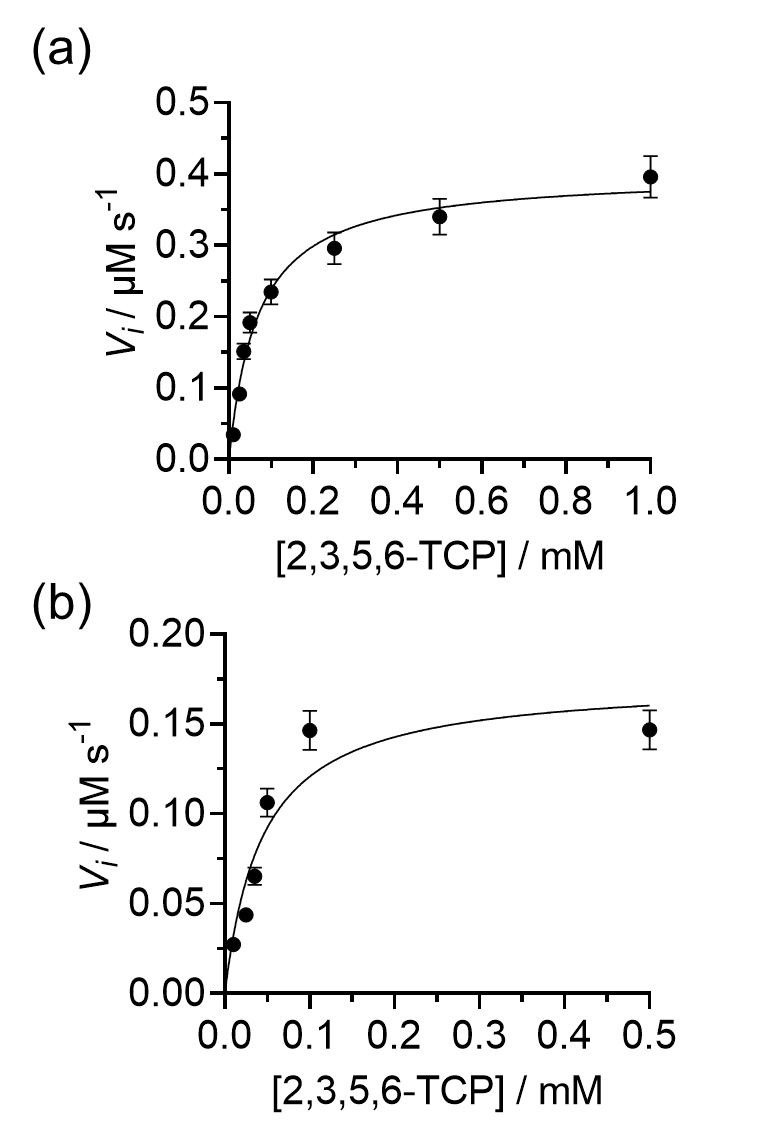
Supplementary Figure 37.** The initial catalytic velocities of (a) Fmoc-K/GMP/Cu^2+^ and (b) laccase at different concentrations of 2,3,5,6-TCP as the reducing substrate. [Fmoc-K] = 5 mM, [GMP]= 10 mM, [Cu^2+^] = 5 μM. [Laccase] =5 μM. The data in (a) and (b) are presented as the mean ± s.d., with the error bars representing the s.d. and n = 3 independent experiments.

**
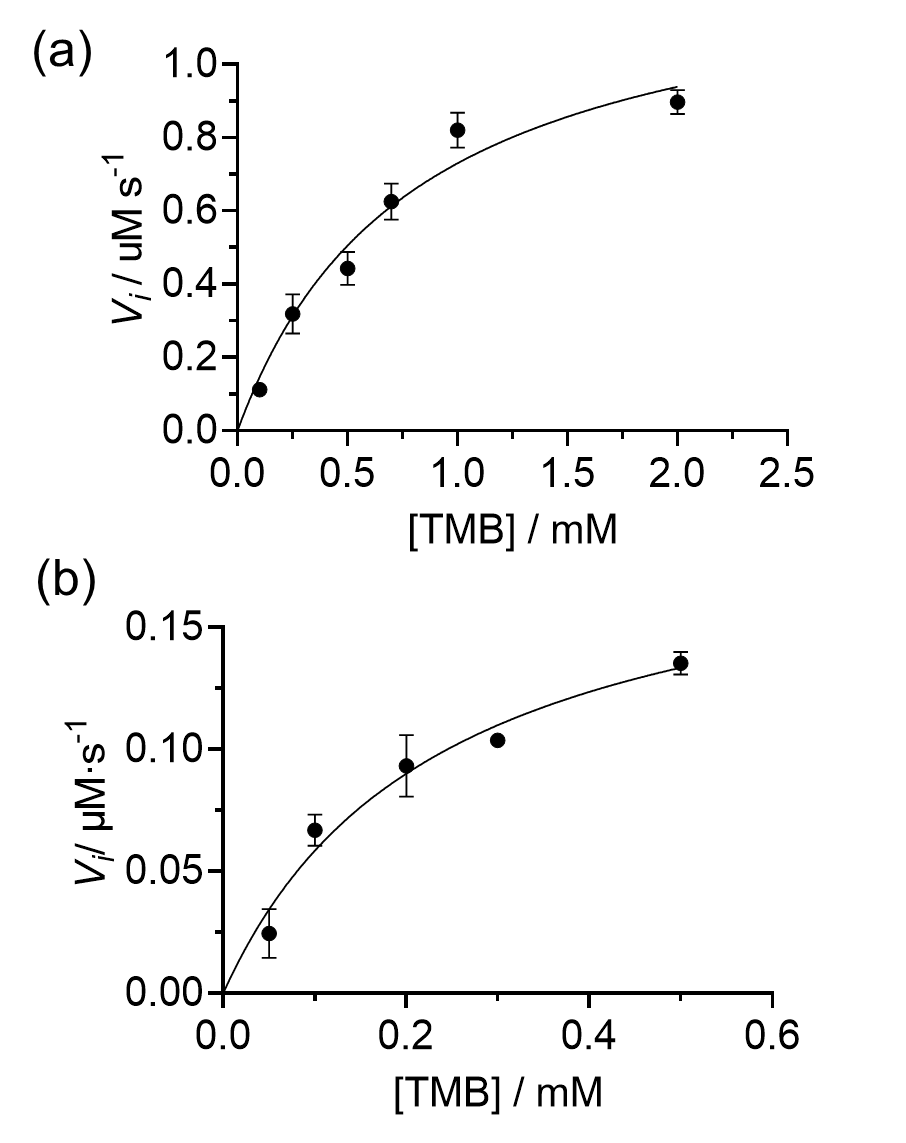
Supplementary Figure 38.** The initial catalytic velocities of (a) Fmoc-K/GMP/Cu^2+^ and (b) laccase at different concentrations of TMB as the reducing substrate. [Fmoc-K] = 5 mM, [GMP]= 10 mM, [Cu^2+^] = 1 μM. [Laccase] =3 μM. The data in (a) and (b) are presented as the mean ± s.d., with the error bars representing the s.d. and n = 3 independent experiments.

**
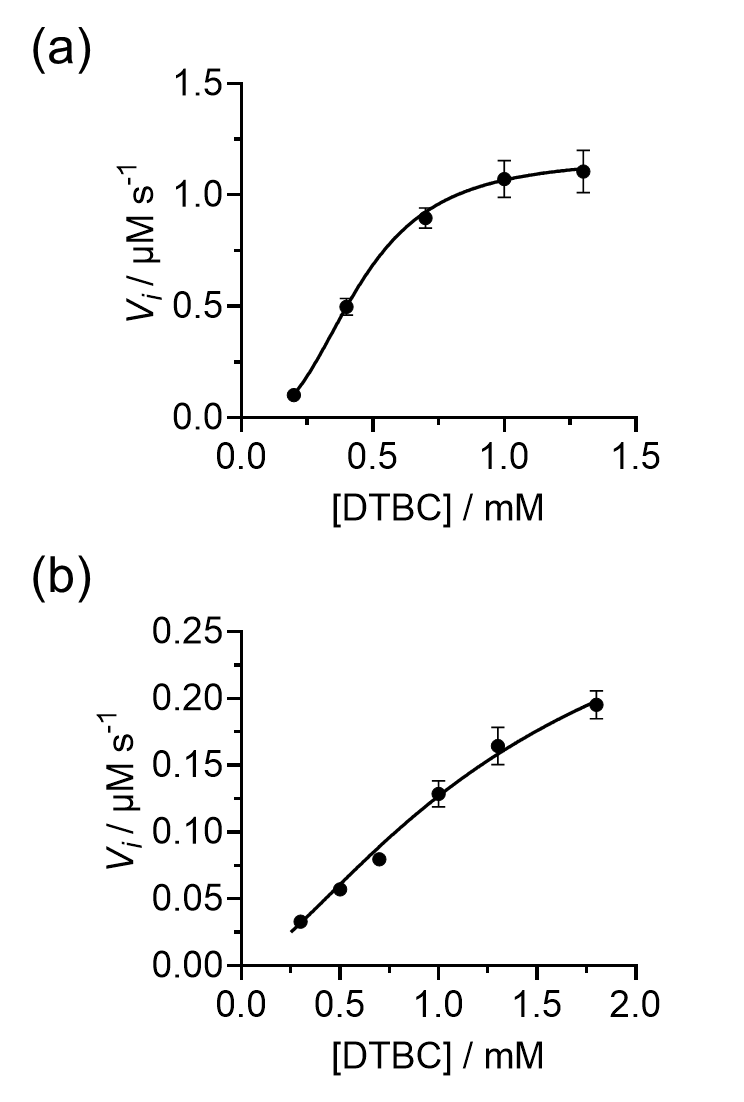
Supplementary Figure 39.** The initial catalytic velocities of (a) Fmoc-K/GMP/Cu^2+^ and (b) laccase at different concentrations of 3,5-DTBC as the reducing substrate. [Fmoc-K] = 5 mM, [GMP]= 10 mM, [Cu^2+^] = 1 μM, [Laccase] =8 μM. The data in (a) and (b) are presented as the mean ± s.d., with the error bars representing the s.d. and n = 3 independent experiments.

**
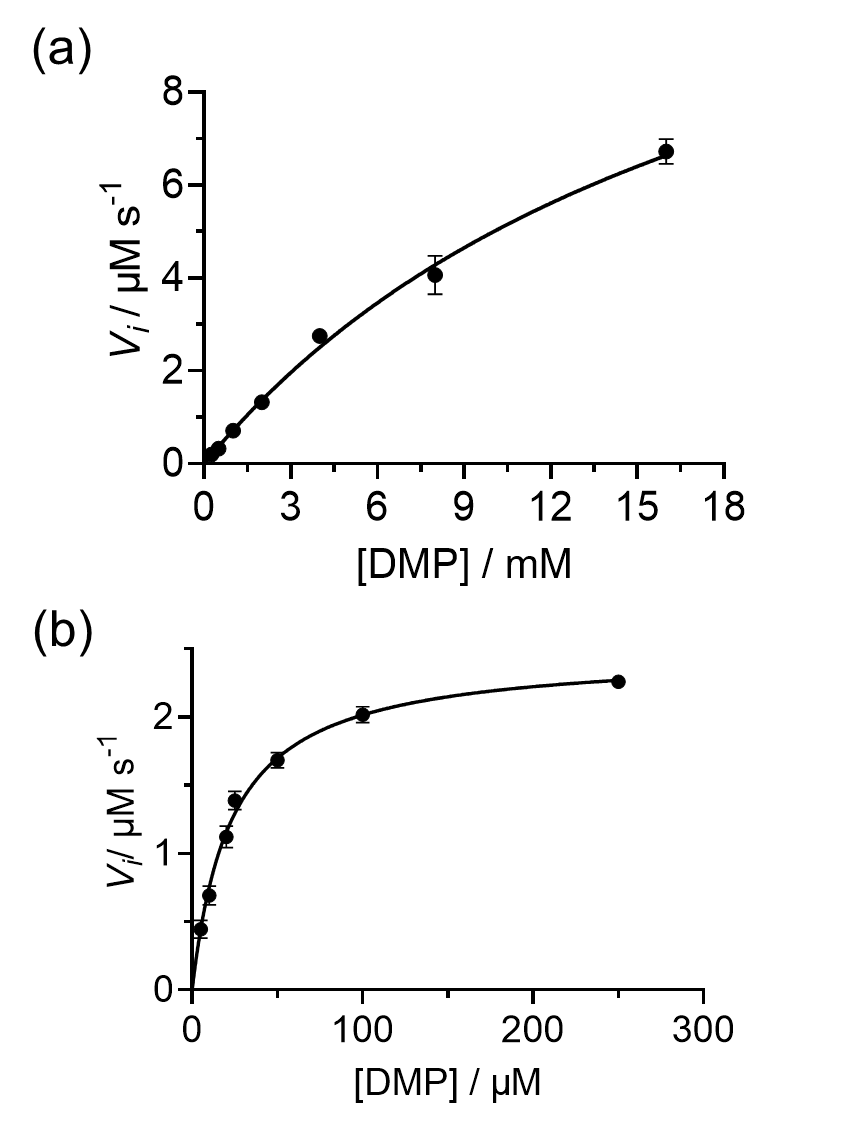
Supplementary Figure 40.** The initial catalytic velocities of (a) Fmoc-K/GMP/Cu^2+^ and (b) laccase at different concentrations of DMP as the reducing substrate. [Fmoc-K] = 5 mM, [GMP]= 10 mM, [Cu^2+^] = 5 μM, [Laccase] =5 μM. The data in (a) and (b) are presented as the mean ± s.d., with the error bars representing the s.d. and n = 3 independent experiments.

**Supplementary Figure 41.** (a) Time-dependent absorbance changes at 510 nm for the catalyzed oxidation of 2,4-DCP in the presence of 4-AP by Fmoc-K/GMP/Cu^2+^ and tyrosinase. [Fmoc-K] = 5 mM, [GMP]= 10 mM, [Cu^2+^] = 5 μM, [Tyrosinase] = 2.5 μM. (b) The initial catalytic velocities of Fmoc-K/GMP/Cu^2+^ and tyrosinase, per μM Cu^2+^. The concentration of Cu^2+^ in tyrosinase sample was determined by ICP-MS. The data in (b) are presented as the mean ± s.d., with the error bars representing the s.d. and n = 3 independent experiments.


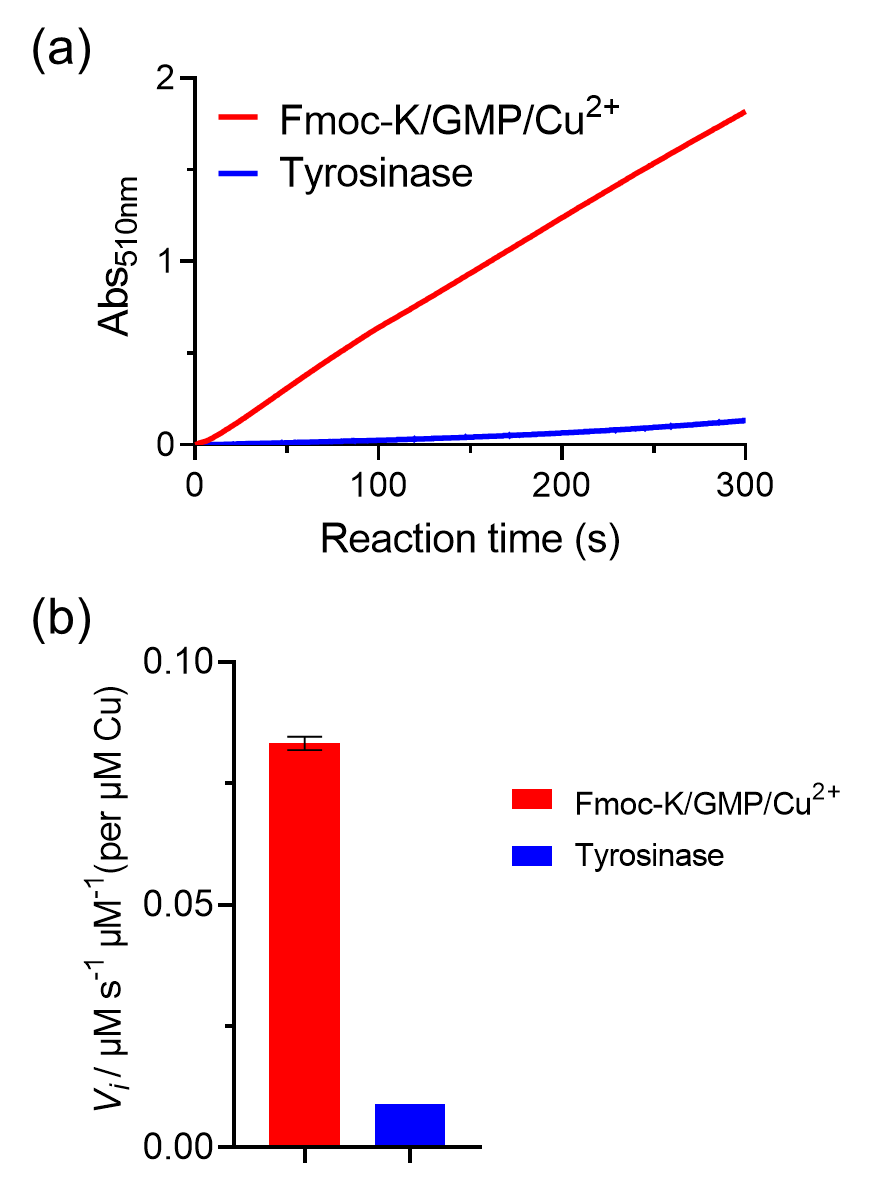


**
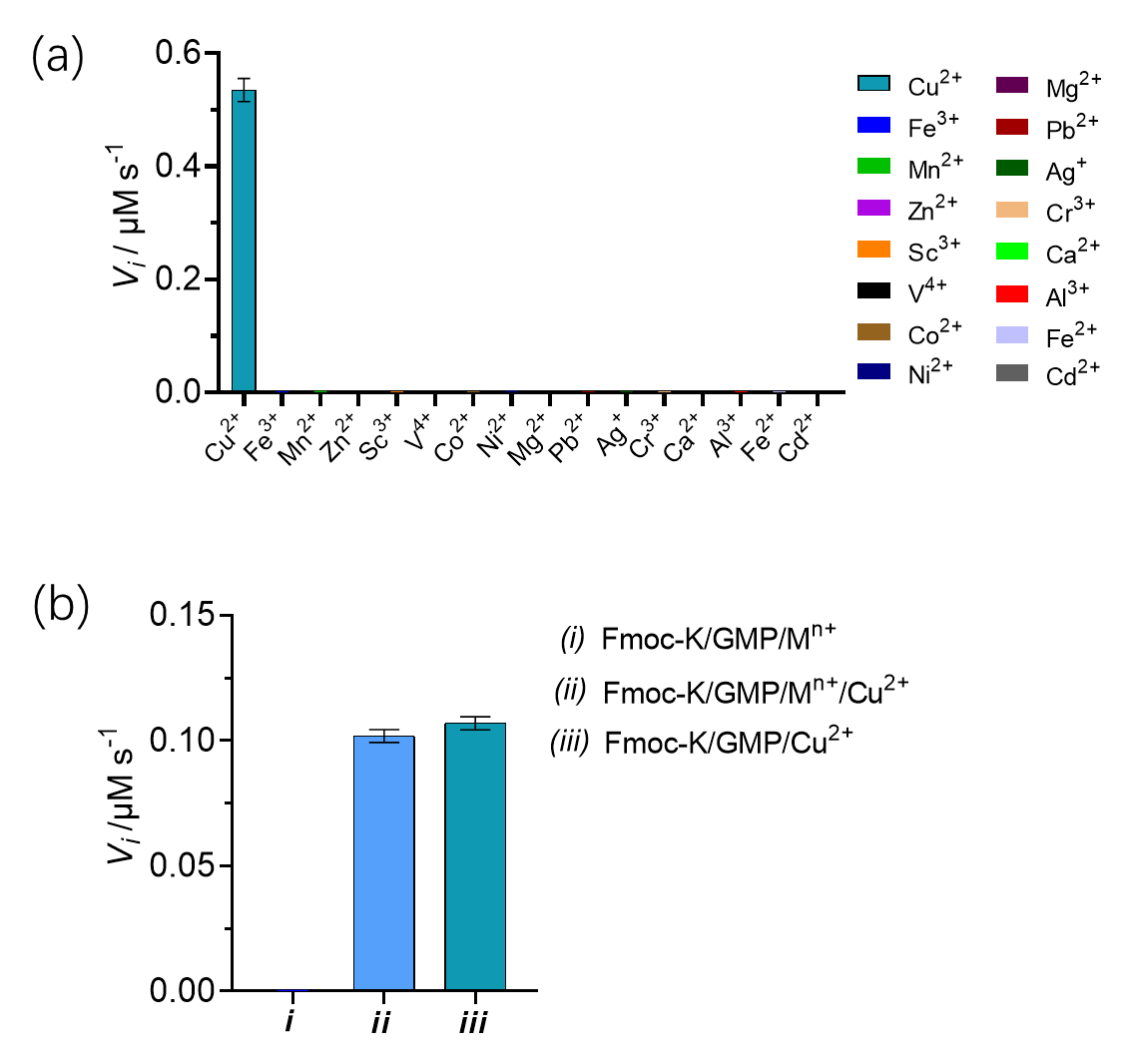
Supplementary Figure 42.** (a) The initial catalytic velocities of the Fmoc-K/GMP-based complexes containing different metal ions. [Fmoc-K] = 5 mM, [GMP] = 10 mM, [Metal ions] = 5 μM. (b) The initial catalytic velocities of the Fmoc-K/GMP-based complexes, M^n+^ stands for the mixture of Zn^2+^, Fe^3+^, Mn^2+^, Sc^3+^, V^4+^, Co^2+^, Ni^2+^, Mg^2+^, Ag^+^, Pb^2+^, Cr^3+^, Ca^2+^, Al^3+^ and Cd^2+^. Each [M^n+^] = 1 μM. [Cu^2+^] = 1 μM. The data are presented as the mean ± s.d., with the error bars representing the s.d. and n = 3 independent experiments.

**Supplementary Figure 43.** Dependence of the initial catalytic velocities of Fmoc-H/GMP/Cu^2+^ on the concentrations of 2,4-DCP. [Fmoc-H] = 5 mM, [GMP]= 10 mM, [Cu^2+^] = 5 μM. The data are presented as the mean ± s.d., with the error bars representing the s.d. and n = 3 independent experiments.

**Supplementary Figure 44.** The initial catalytic velocities of Fmoc-K/GMP/Cu^2+^, Fmoc-R/GMP/Cu^2+^ and Fmoc-H/GMP/Cu^2+^ at different concentrations of Cu^2+^. [Fmoc-K] = 1 mM, [Fmoc-R] = 1 mM, [Fmoc-H] = 1 mM, [GMP]= 2 mM. The data are presented as the mean ± s.d., with the error bars representing the s.d. and n = 3 independent experiments.

**
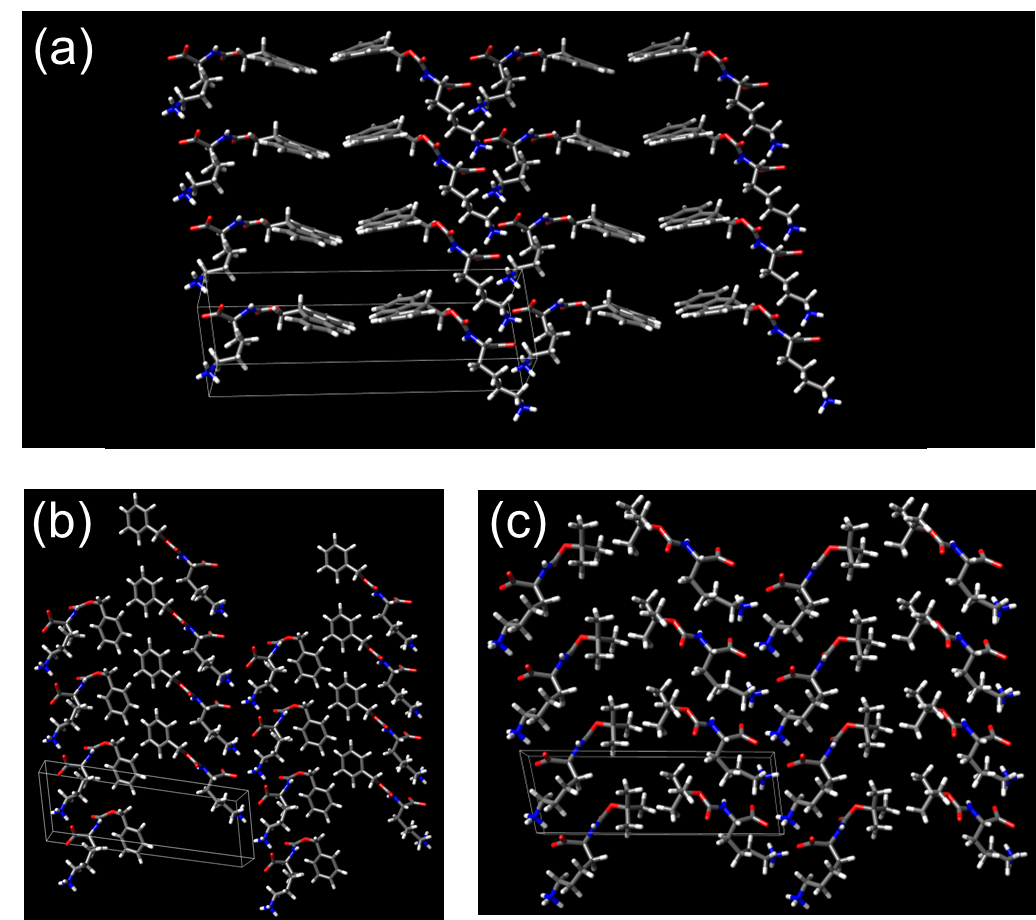
Supplementary Figure 45.** Theoretical models of (a) Fmoc-K, (b) Cbz-K and (c) Boc-K. N, O, C, H, Cu atoms are indicated in blue, red, dim grey, light grey and orange.

**
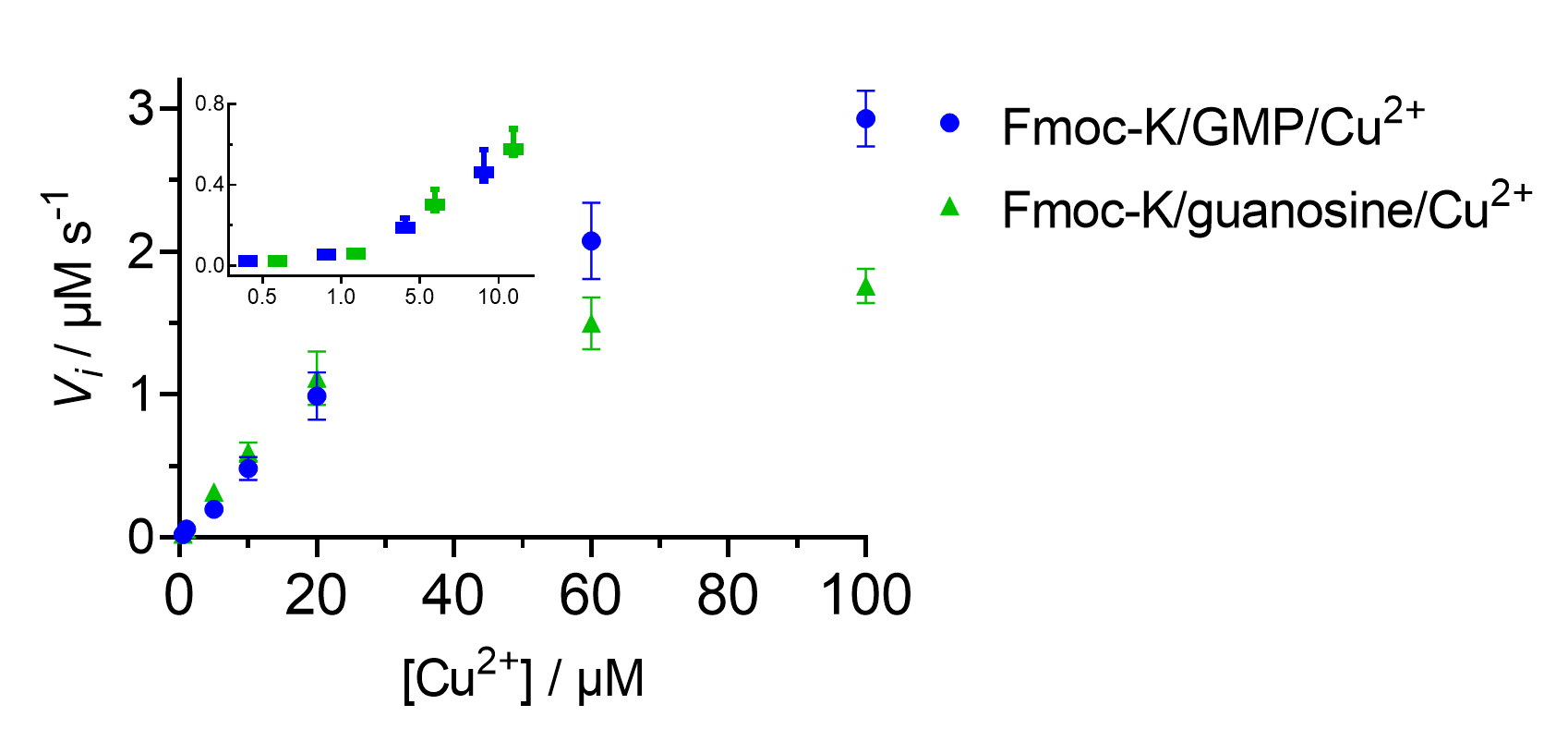
Supplementary Figure 46.** The initial catalytic velocities of Fmoc-K/GMP/Cu^2+^ and Fmoc-K/guanosine/Cu^2+^ at different concentrations of Cu^2+^. [Fmoc-K] = 1 mM, [GMP]= 2 mM, [guanosine] = 2 mM. The data are presented as the mean ± s.d., with the error bars representing the s.d. and n = 3 independent experiments.

**
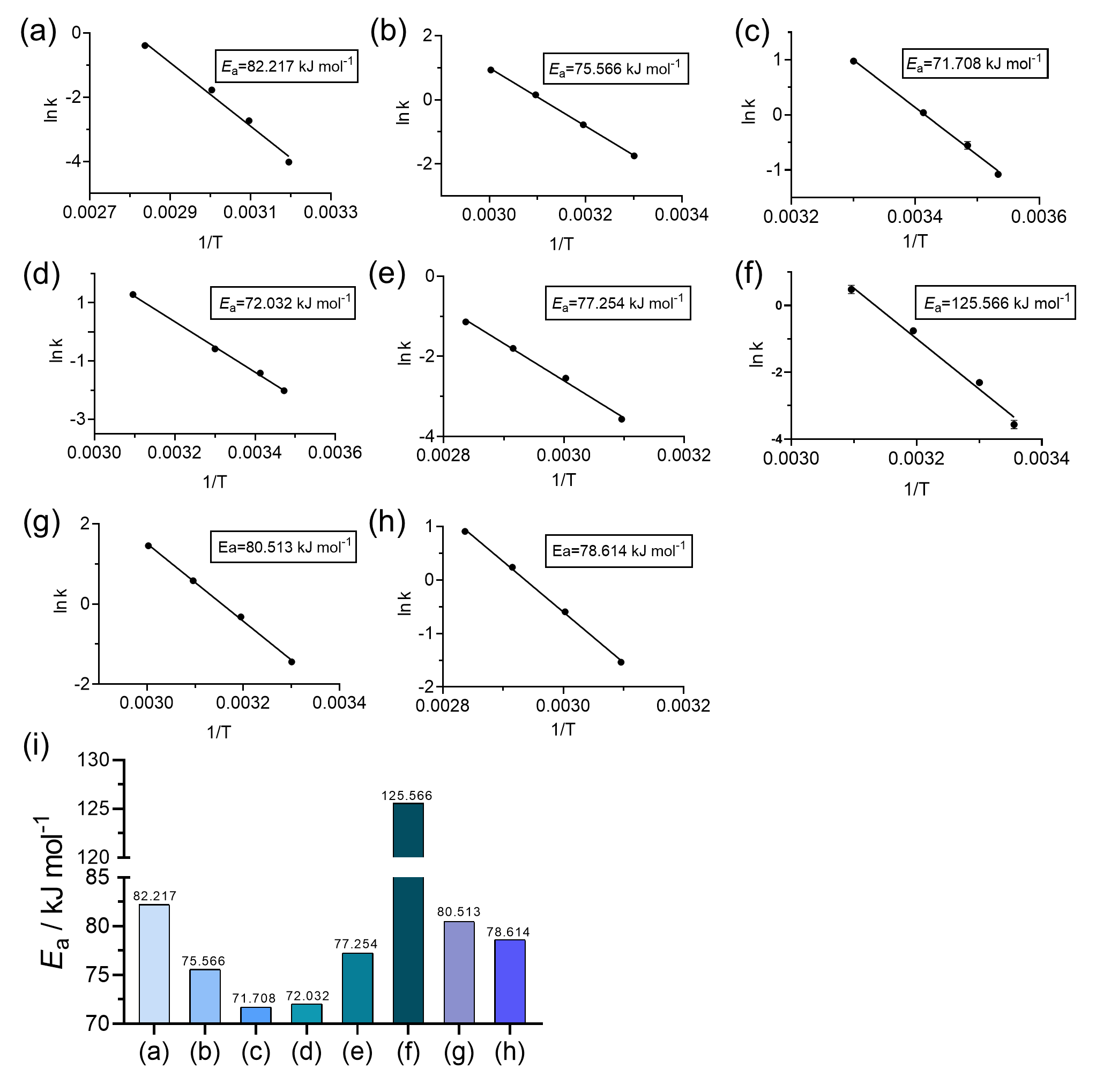
Supplementary Figure 47.** Arrhenius plots for (a) GMP/Cu^2+^, (b) Fmoc-K/Cu^2+^, (c) Fmoc-K/GMP/Cu^2+^, (d) Fmoc-H/GMP/Cu^2+^, (e) Cbz-K/GMP/Cu^2+^, (f) Fmoc-H/Cu^2+^, (g) Fmoc-K/UMP/Cu^2+^, (h) Fmoc-K/GTP/Cu^2+^. (i) Activation energies (*E*_a_) of different complexes. [Fmoc-K] = 5 mM, [Fmoc-H] = 5 mM, [Cbz-K] = 5 mM, [GMP]= 10 mM, [UMP]= 10 mM, [GTP]= 10 mM, [Cu^2+^] = 5 μM.

**
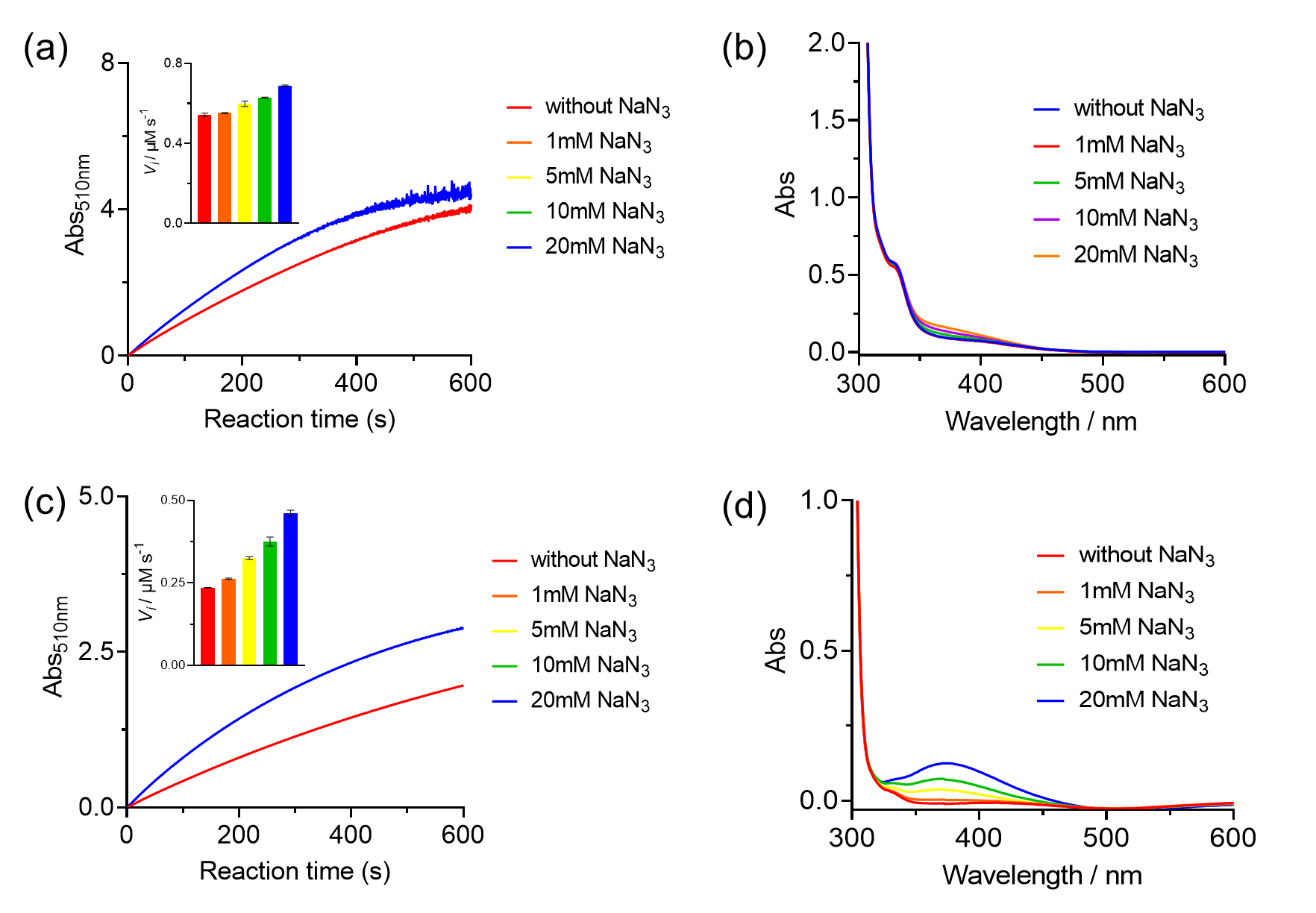
Supplementary Figure 48.** (a) Time-dependent absorbance changes at 510 nm and the initial catalytic velocities (inset) for Fmoc-K/GMP/Cu^2+^ after adding different concentrations of NaN_3_. [Fmoc-K] = 5 mM, [GMP] = 10 mM, [Cu^2+^] = 5μM. (b) UV-vis spectra of Fmoc-K/GMP/Cu^2+^ after adding different concentrations of NaN_3_. [Fmoc-K] = 5 mM, [GMP] = 10 mM, [Cu^2+^] = 100μM. (c) Time-dependent absorbance changes at 510 nm and the initial catalytic velocities (inset) for Fmoc-K/GMP/Cu^2+^ after adding different concentrations of NaN_3_. [Fmoc-K] = 1 mM, [GMP] = 2 mM, [Cu^2+^] = 5 μM. (d) UV-vis spectra of Fmoc-K/GMP/Cu^2+^ after adding different concentrations of NaN_3_. [Fmoc-K] = 1 mM, [GMP] = 2 mM, [Cu^2+^] = 100 μM. The data in (a) and (c) are presented as the mean ± s.d., with the error bars representing the s.d. and n = 3 independent experiments.

**Supplementary Figure 49.** Time-dependent absorbance changes at 510 nm for Fmoc-K/Cu^2+^, Fmoc-K/GMP/Cu^2+^, and Fmoc-K/NaN_3_/Cu^2+^. [Fmoc-K] = 1 mM, [GMP] = 2 mM, [NaN_3_] = 2 mM, [Cu^2+^] = 5 μM.

**
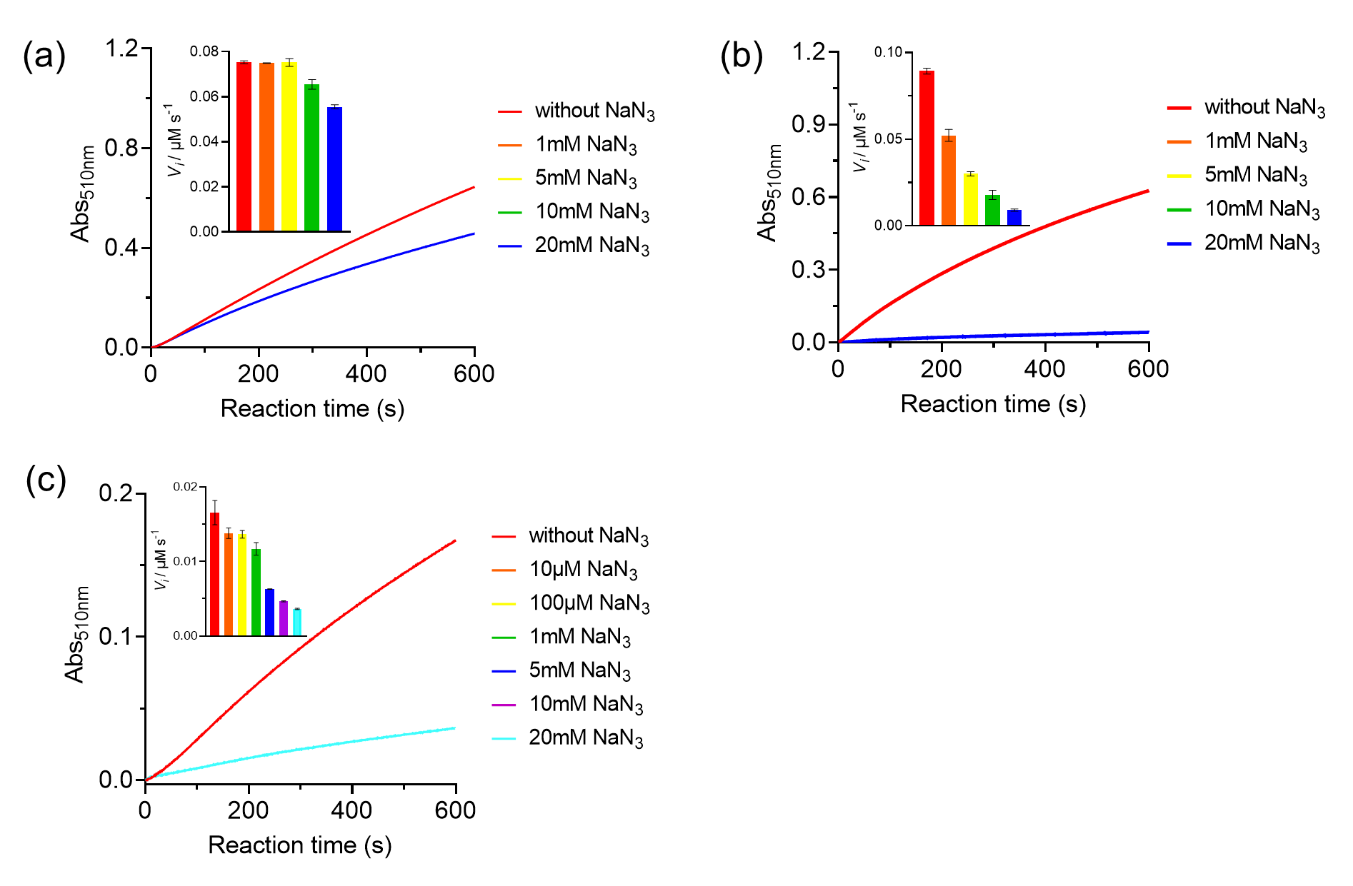
Supplementary Figure 50.** Time-dependent absorbance changes at 510 nm and the initial catalytic velocities (inset) for Fmoc-H/GMP/Cu^2+^ after adding different concentrations of NaN_3_. (a) Fmoc-H/GMP/Cu^2+^, [Fmoc-H] = 5 mM, [GMP] = 10 mM, [Cu^2+^] = 5 μM. (b) Fmoc-H/GMP/Cu^2+^, [Fmoc-H] = 1 mM, [GMP] = 2 mM, [Cu^2+^] = 5 μM. (c) Fmoc-H/Cu^2+^, [Fmoc-H] = 1 mM, [Cu^2+^] = 5 μM. The data in (a), (b) and (c) are presented as the mean ± s.d., with the error bars representing the s.d. and n = 3 independent experiments.

**
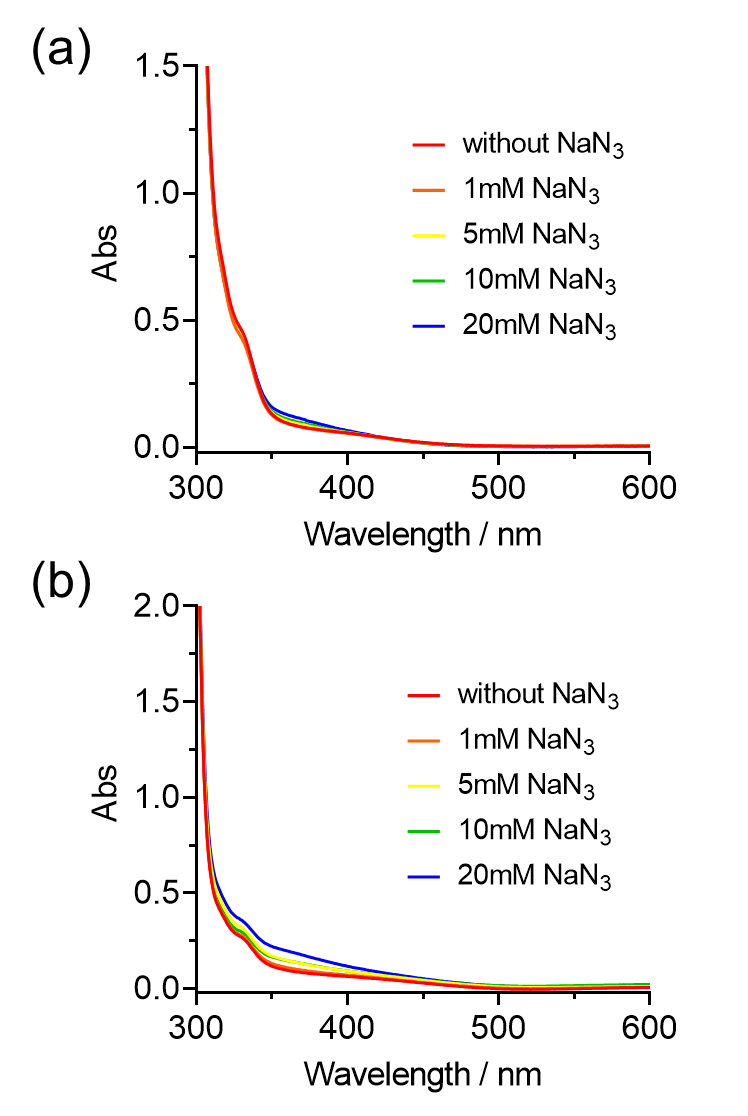
Supplementary Figure 51.** UV-vis spectra of Fmoc-H/GMP/Cu^2+^ after adding different concentrations of NaN_3_. (a) Fmoc-H/GMP/Cu^2+^, [Fmoc-H] = 5 mM, [GMP] = 10 mM, [Cu^2+^] = 100 μM. (b) Fmoc-H/GMP/Cu^2+^, [Fmoc-H] = 1 mM, [GMP] = 2 mM, [Cu^2+^] = 100 μM.

**
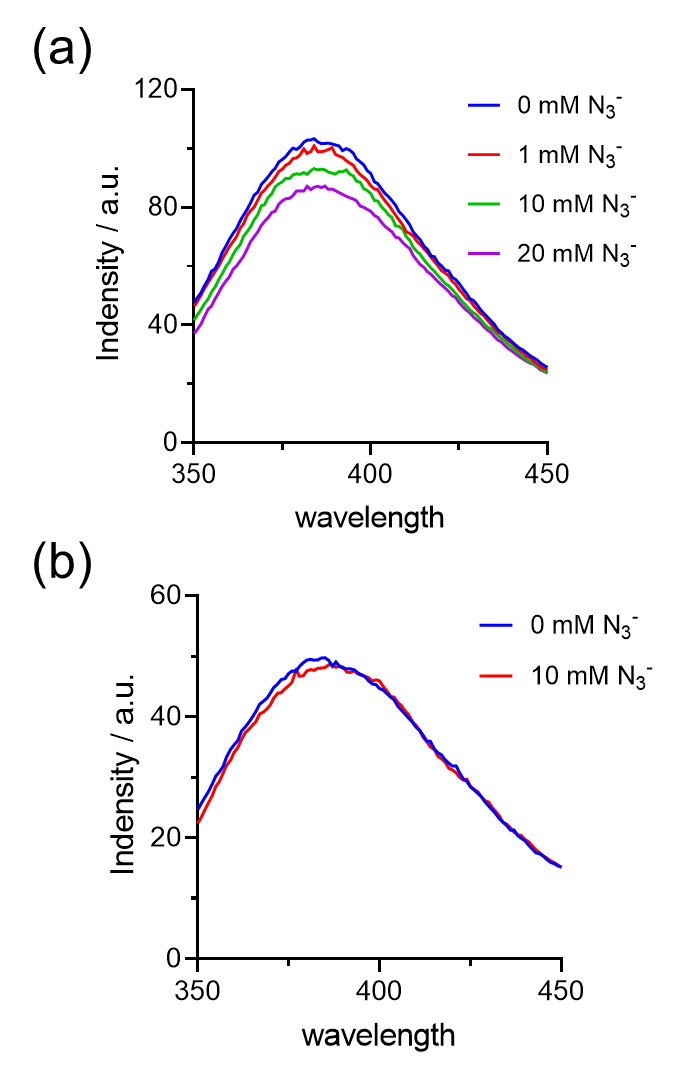
Supplementary Figure 52.** Fluorescence spectra of (a) Fmoc-H and (b) Fmoc-K with N_3_^-^.

**
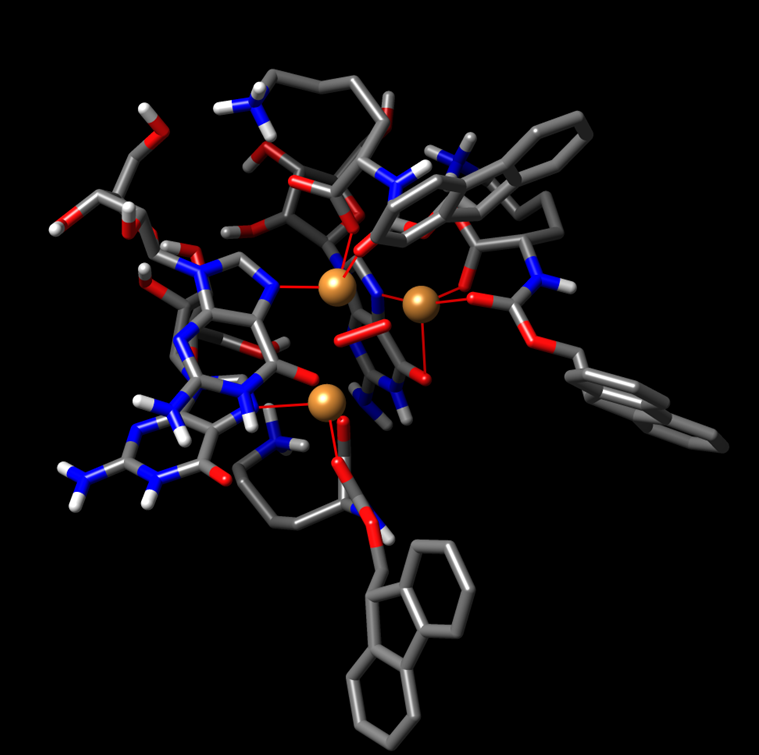
Supplementary Figure 53.** Density functional theory model of the T2-T3Cu/O_2_. N, O, C, H, Cu atoms are indicated in blue, red, dim grey, light grey and orange.

**Supplementary Figure 54.** The initial catalytic velocities of the Fmoc-H/Cu^2+^ complexes containing various nucleotides at different Cu^2+^ concentrations. [Fmoc-H] = 1mM, [GMP] = 2 mM, [CMP] = 2 mM, [AMP] = 2 mM, [UMP] = 2 mM, [Cu^2+^] = 5 μM. The data are presented as the mean ± s.d., with the error bars representing the s.d. and n = 3 independent experiments.

**
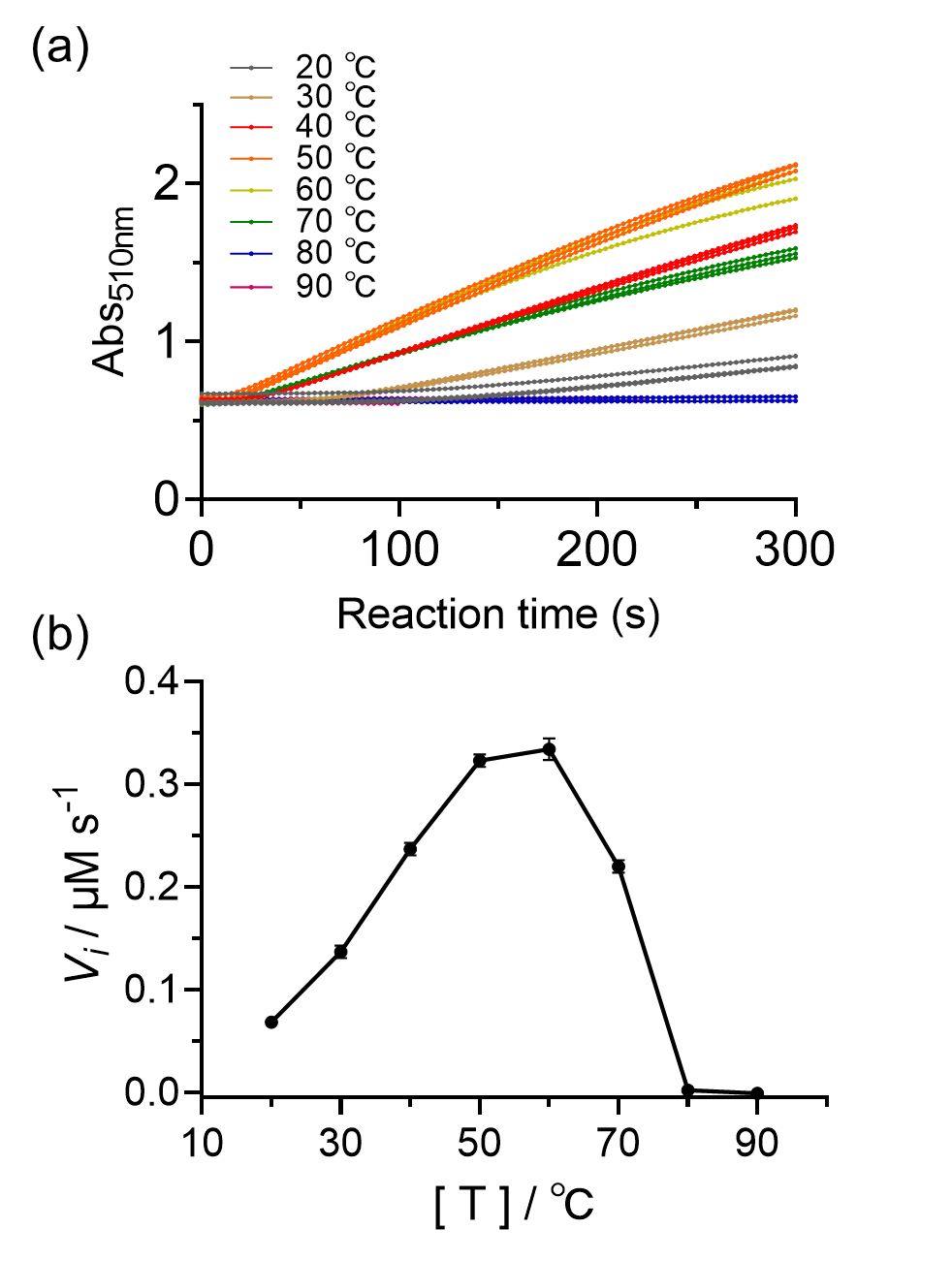
Supplementary Figure 55.** (a) Time-dependent absorbance changes at 510 nm for laccase-catalyzed oxidations of 2,4-DCP at different temperature. (b) Dependence of the initial catalytic velocities of laccase on the reaction temperature. [Laccase] = 0.75 μM. The data in (b) are presented as the mean ± s.d., with the error bars representing the s.d. and n = 3 independent experiments.

**Supplementary Figure 56.** Dependence of the initial catalytic velocities of Fmoc-K/GMP/Cu^2+^ on the Cu^2+^ concentrations at 80^o^C. [Fmoc-K] = 5 mM, [GMP] = 10 mM. The data are presented as the mean ± s.d., with the error bars representing the s.d. and n = 3 independent experiments.

**
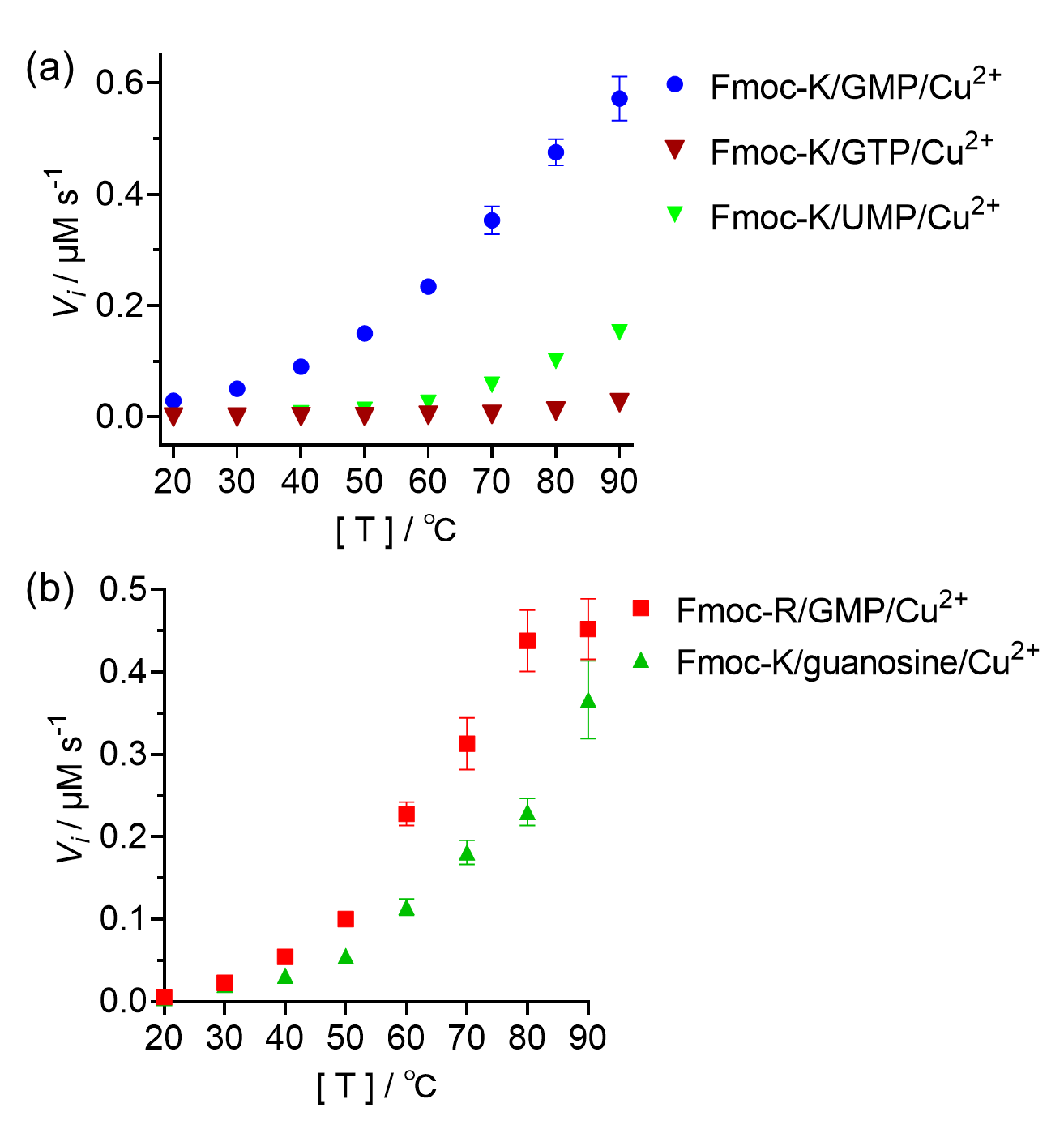
Supplementary Figure 57.** Dependence of the initial catalytic velocities of different Cu^2+^-contained complexes on the reaction temperature. (a) [Fmoc-K] = 5 mM, [GMP] = 10 mM, [GTP] = 10 mM, [UMP] = 10 mM, [Cu^2+^] = 0.3 μM. (b) [Fmoc-R] = 5 mM, [Fmoc-K] = 5 mM, [GMP] = 2 mM, [guanosine] = 2 mM, [Cu^2+^] = 0.3 μM. The data in (a) and (b) are presented as the mean ± s.d., with the error bars representing the s.d. and n = 3 independent experiments.

**
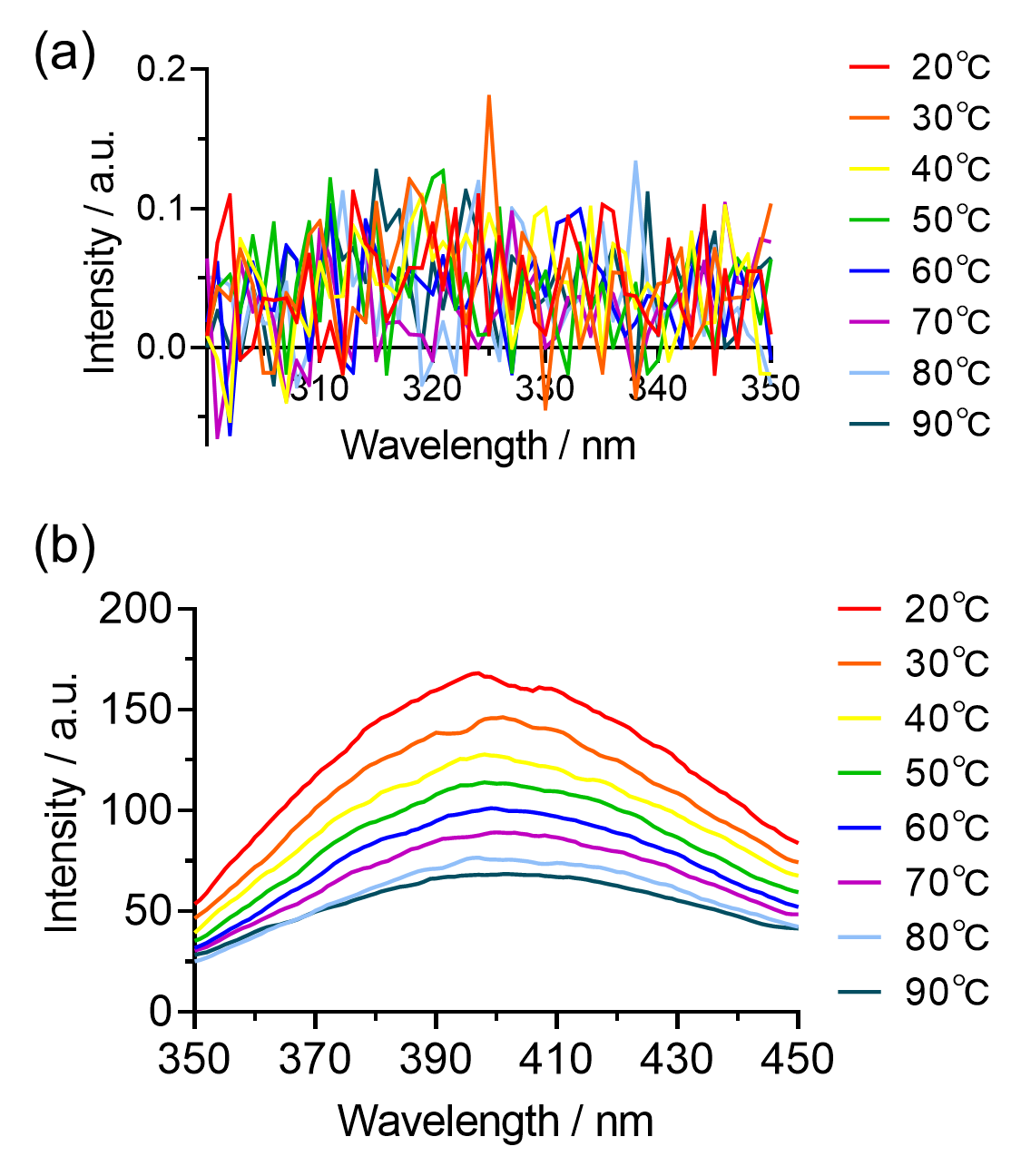
Supplementary Figure 58.** Fluorescence spectra of the Fmoc-K/GMP/Cu^2+^ complex when it was excited at (a) 290 nm and (b) 320 nm. [Fmoc-K] = 5 mM, [GMP] = 10 mM, [Cu^2+^] = 5 μM.

**Supplementary Figure 59.** The initial catalytic velocities of Fmoc-K/GMP/Cu^2+^ after incubation at 80^o^C over different time. [Fmoc-K] = 5 mM, [GMP] = 10 mM, [Cu^2+^] = 0.3 μM. The data are presented as the mean ± s.d., with the error bars representing the s.d. and n = 3 independent experiments.

**Supplementary Figure 60.** The initial catalytic velocities of Fmoc-K/GMP/Cu^2+^ and laccase when they were cycled between 25^o^C and 80^o^C. No catalyzed oxidation of 2,4-DCP by laccase was observed at 80^o^C, so the values at 80^o^C were not indicated. In the inset, the y-axis is enlarged. [Fmoc-K] = 5 mM, [GMP] = 10 mM, [Cu^2+^] = 0.3 μM, [Laccase] = 5 μM. The data are presented as the mean ± s.d., with the error bars representing the s.d. and n = 3 independent experiments.


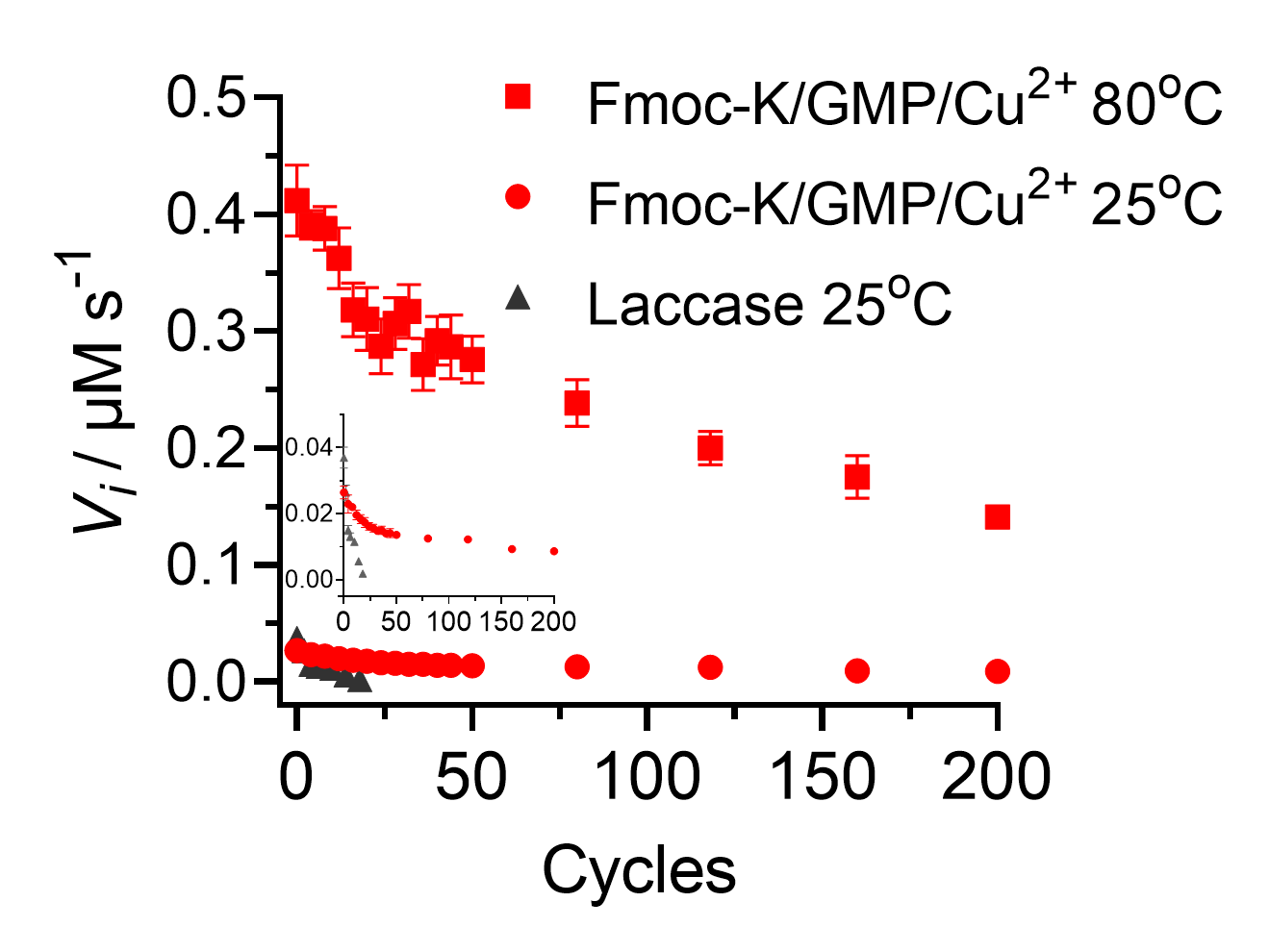


**Supplementary Figure 61.** Effect of cycled acidification and neutralization treatments on the catalysis. [Cu^2+^] = 5 μM, [Fmoc-K] = 5mM, [GMP] = 10 mM, [laccase] = 5 μM. The data are presented as the mean ± s.d., with the error bars representing the s.d. and n = 3 independent experiments.

**Supplementary Figure 62.** Dependence of the initial catalytic velocities of high concentrations of salt. [Cu^2+^] = 5 μM, [Fmoc-K] = 1mM, [GMP] = 2 mM, [2,4-DCP] = 1 mM, [4-AP] = 1mM. The data are presented as the mean ± s.d., with the error bars representing the s.d. and n = 3 independent experiments.

**Supplementary Table 1.** The EPR parameters (g_┴_, g//, A//, Δg_┴_ and Δg//) of Cu^2+^ complex.


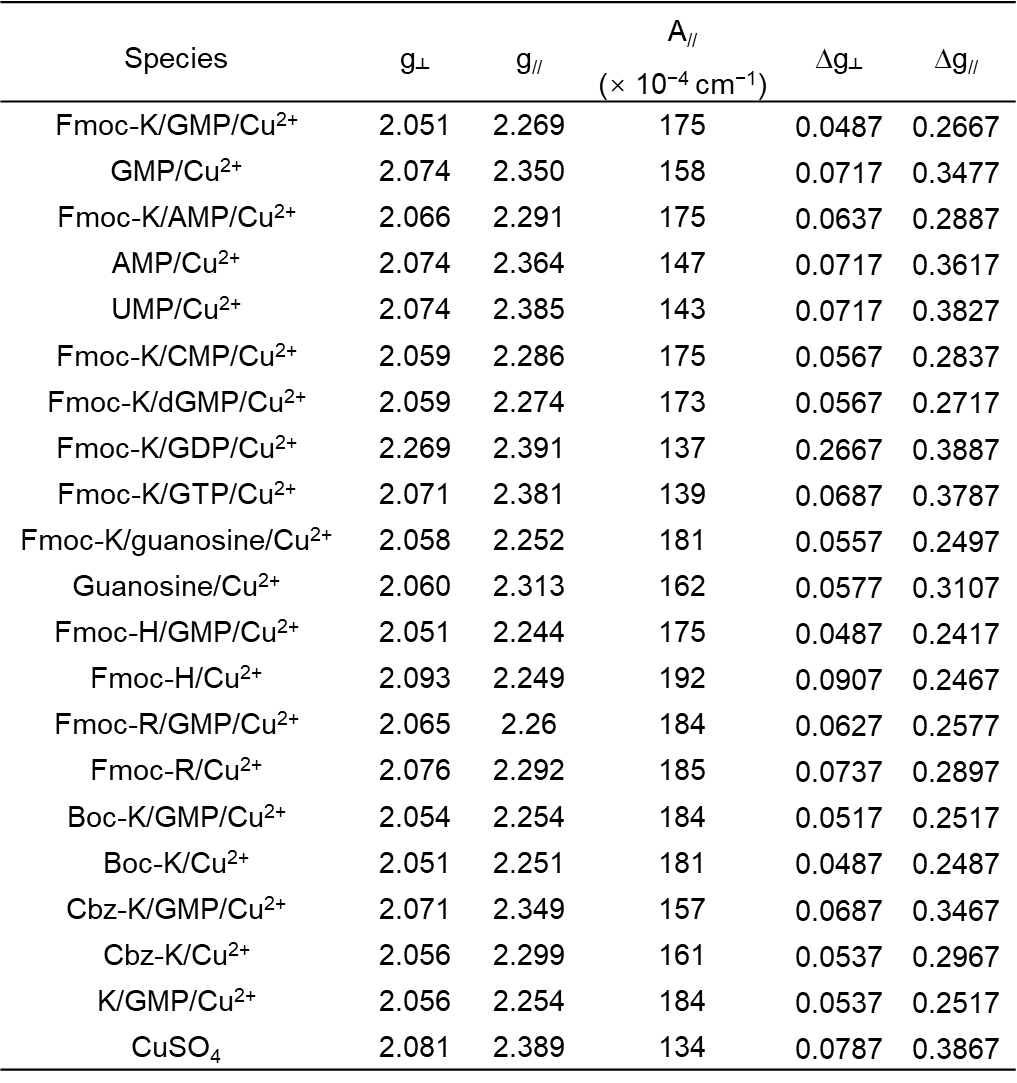


**
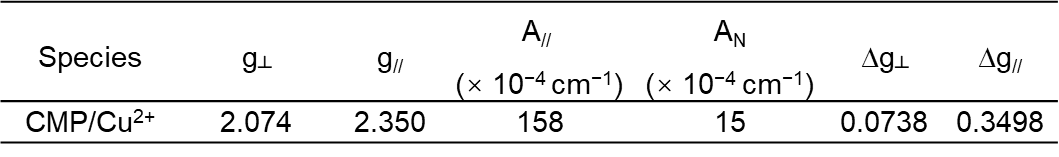
Supplementary Table 2.** The EPR parameters (g_┴_, g//, A//, A_N_, Δg_┴_ and Δg//) of Cu^2+^ complex.

**
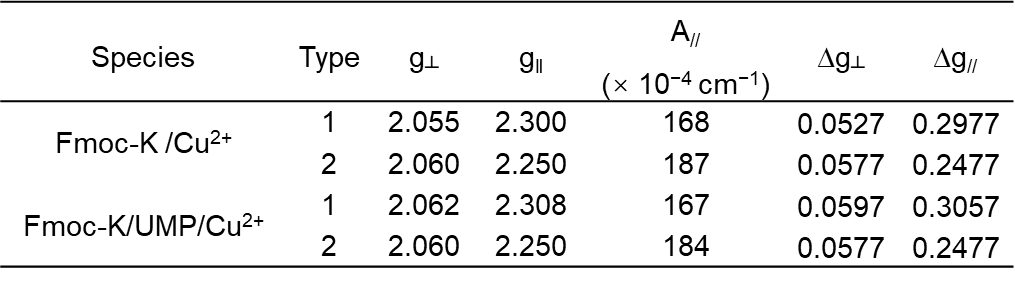
Supplementary Table 3.** The EPR parameters (g_┴_, g//, A//, Δg_┴_ and Δg//) of Cu^2+^ complex.

**
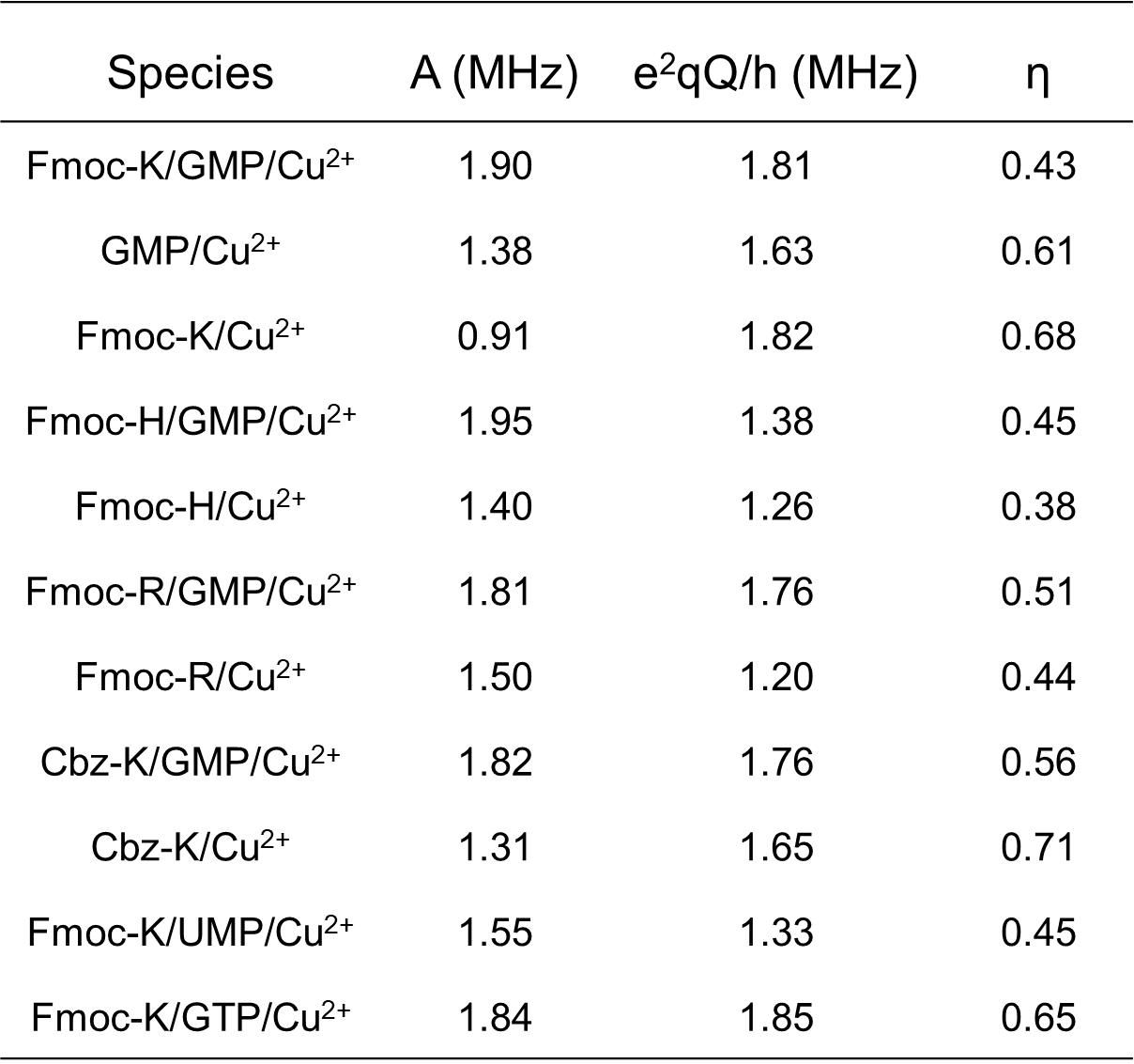
Supplementary Table 4.** The three-pluse ESEEM parameters (A, *k* and η) of Cu^2+^ complex.

**Supplementary Table 5.** Comparison of the apparent kinetic parameters of Fmoc-K/GMP/Cu^2+^ with the previously reported oxidase-mimetic catalysts. [Fmoc-K = 5 mM], [GMP] = 10 mM, [Cu^2+^] = 5 μM.


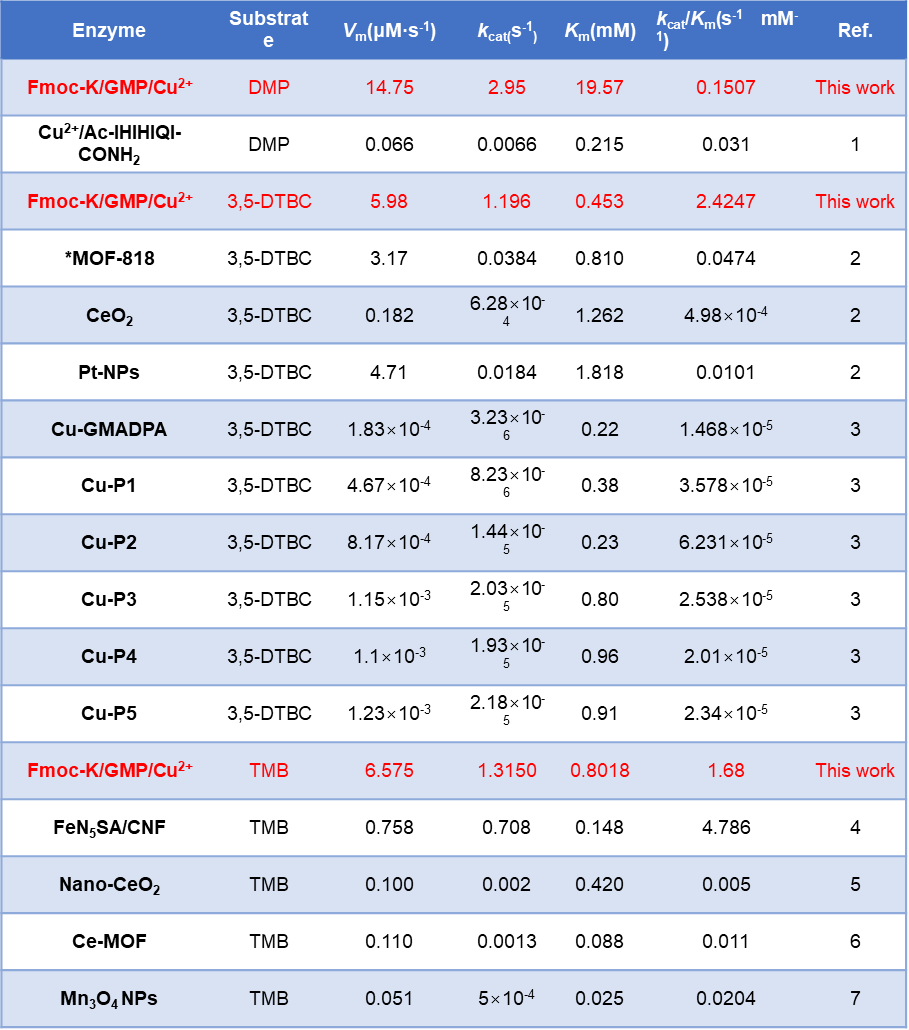


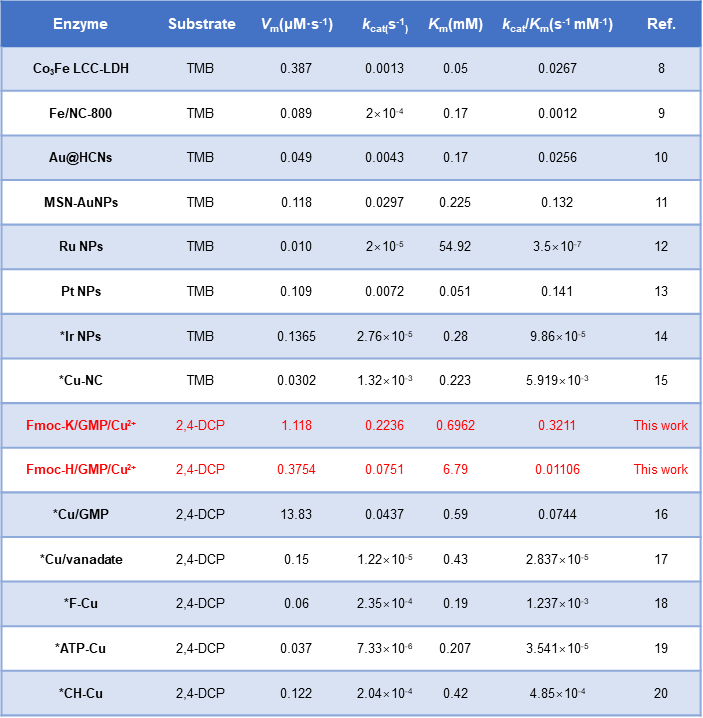


**Supplementary Reference**

1. Makhlynets, O. V., Gosavi, P. M. & Korendovych, I. V. Short self-assembling peptides are able to bind to copper and activate oxygen. *Angew. Chem. Int. Ed.* **55**, 9017-9020 (2016).

2. Li, M., Chen, J., Wu, W., Fang, Y. & Dong, S. Oxidase-like MOF-818 nanozyme with high specificity for catalysis of catechol oxidation. *J. Am. Chem. Soc.* **142**, 15569-15574 (2020).

3. Thanneeru, S. et al. Synthetic polymers to promote cooperative Cu activity for O_2_ activation: poly vs mono. *J. Am. Chem. Soc.* **141**, 4252-4256 (2019).

4. Huang, L., Chen, J., Gan, L., Wang, J. & Dong, S. Single-atom nanozymes. *Sci. Adv.* **5**, eaav5490 (2019).

5. Cheng, H., Lin, S., Muhammad, F., Lin, Y.-W. & Wei, H. Rationally modulate the oxidase-like activity of nanoceria for self-regulated bioassays. *ACS Sens.* **1**, 1336-1343 (2016).

6. Dalapati, R., Sakthivel, B., Ghosalya, M. K., Dhakshinamoorthy, A. & Biswas, S. A cerium-based metal–organic framework having inherent oxidase-like activity applicable for colorimetric sensing of biothiols and aerobic oxidation of thiols. *CrystEngComm* **19**, 5915-5925 (2017).

7. Zhang, X. & Huang, Y. Evaluation of the antioxidant activity of phenols and tannic acid determination with Mn_3_O_4_ nano-octahedrons as an oxidase mimic. *Anal. Methods* **7**, 8640-8646 (2015).

8. Zhao, J. et al. A hierarchical Co-Fe LDH rope-like nanostructure: facile preparation from hexagonal lyotropic liquid crystals and intrinsic oxidase-like catalytic activity. *J. Mater. Chem. B* **1**, 1263-1269 (2013).

9. Chen, Q., Liang, C., Zhang, X. & Huang, Y. High oxidase-mimic activity of Fe nanoparticles embedded in an N-rich porous carbon and their application for sensing of dopamine. *Talanta* **182**, 476-483 (2018).

10. Fan, L. et al. Tumor Catalytic-photothermal therapy with yolk-shell gold@carbon nanozymes. *ACS Appl. Mater. Interfaces* **10**, 4502-4511 (2018).

11. Tao, Y., Ju, E., Ren, J. & Qu, X. Bifunctionalized mesoporous silica-supported gold nanoparticles: intrinsic oxidase and peroxidase catalytic activities for antibacterial applications. *Adv. Mater.* **27**, 1097-1104 (2015).

12. Cao, G.-J., Jiang, X., Zhang, H., Croley, T. R. & Yin, J.-J. Mimicking horseradish peroxidase and oxidase using ruthenium nanomaterials. *RSC Adv.* **7**, 52210-52217 (2017).

13. Guo, L., Mao, L., Huang, K. & Liu, H. Pt–Se nanostructures with oxidase-like activity and their application in a selective colorimetric assay for mercury(II). *J. Mater. Sci.* **52**, 10738-10750 (2017).

14. Cui, M., Zhao, Y., Wang, C. & Song, Q. The oxidase-like activity of iridium nanoparticles, and their application to colorimetric determination of dissolved oxygen. *Microchim. Acta* **184**, 3113-3119 (2017).

15. He, F. et al. Oxidase-Inspired Selective 2e/4e Reduction of Oxygen on Electron-Deficient Cu. *ACS Appl. Mater. Interfaces* **12**, 4833-4842 (2020).

16. Liang, H. et al. Multicopper laccase mimicking nanozymes with nucleotides as ligands. *ACS Appl. Mater. Interfaces* **9**, 1352-1360 (2017).

17. Jain, S., Sharma, B., Thakur, N., Mishra, S. & Sarma, T. K. Copper pyrovanadate nanoribbons as efficient multienzyme Mimicking Nanozyme for Biosensing Applications. *ACS Appl. Nano Mater.* **3**, 7917-7929 (2020).

18. Makam, P. et al. Single amino acid bionanozyme for environmental remediation. *Nat. Commun.* **13**, 1505 (2022).

19. Huang, H. et al. Fluorometric and colorimetric analysis of alkaline phosphatase activity based on a nucleotide coordinated copper ion mimicking polyphenol oxidase. *J. Mater. Chem. B* **7**, 6508-6514 (2019).

20. Wang, J. et al. Construction of a bioinspired laccase-mimicking nanozyme for the degradation and detection of phenolic pollutants. *Appl. Catal. B* **254**, 452-462 (2019).
