# Supplementary material for: A supramolecular metalloenzyme possessing robust oxidase-mimetic catalytic function
Source: Nat Commun. 2023 Jul 7;14:4040. doi: 10.1038/s41467-023-39779-6 (PMC10328989; doi:10.1038/s41467-023-39779-6)
Supplement: Supplementary file 3 — Reporting Summary [file 41467_2023_39779_MOESM3_ESM.pdf]

nature portfolio

Corresponding author(s): Zhen-Gang Wang

Last updated by author(s): Jun 6, 2023

Reporting Summary

Nature Portfolio wishes to improve the reproducibility of the work that we publish. This form provides structure for consistency and transparency in reporting. For further information on Nature Portfolio policies, see our Editorial Policies and the Editorial Policy Checklist.

Statistics

For all statistical analyses, confirm that the following items are present in the figure legend, table legend, main text, or Methods section.

n/a	Confirmed	
☐	☒	The exact sample size (*n*) for each experimental group/condition, given as a discrete number and unit of measurement
☐	☒	A statement on whether measurements were taken from distinct samples or whether the same sample was measured repeatedly
☒	☐	The statistical test(s) used AND whether they are one- or two-sided<br>*Only common tests should be described solely by name; describe more complex techniques in the Methods section.*
☐	☒	A description of all covariates tested
☐	☒	A description of any assumptions or corrections, such as tests of normality and adjustment for multiple comparisons
☐	☒	A full description of the statistical parameters including central tendency (e.g. means) or other basic estimates (e.g. regression coefficient) AND variation (e.g. standard deviation) or associated estimates of uncertainty (e.g. confidence intervals)
☒	☐	For null hypothesis testing, the test statistic (e.g. *F*, *t*, *r*) with confidence intervals, effect sizes, degrees of freedom and *P* value noted<br>*Give P values as exact values whenever suitable.*
☐	☒	For Bayesian analysis, information on the choice of priors and Markov chain Monte Carlo settings
☐	☒	For hierarchical and complex designs, identification of the appropriate level for tests and full reporting of outcomes
☒	☐	Estimates of effect sizes (e.g. Cohen's *d*, Pearson's *r*), indicating how they were calculated

Our web collection on statistics for biologists contains articles on many of the points above.

Software and code

Policy information about availability of computer code

Data collection	AFM was performed in QI mode (Nanowizard 4, JPK, Bruker) and was processed using JPK Data Processor software. Ultraviolet-visible absorption spectra were recorded using a UV-VIS spectrophotometer (UV-2600, Shimadzu) and UVProbe 2.70 software. Fluorescence spectra were recorded using a Cary Eclipse Fluorescence spectrofluorometer (G9800A, Agilent Technologies) and Cary winFLR software (version: 1.2). SEM images were obtained using a scanning electron microscope (MAIA3, TESCAN) and a MAIA3TC software setup. TEM images were obtained with an electron electron microscope (HT7800, HITACHI) and a XR81-B-M1-BT-FX software setup. EPR experiments were performed on an ELEXSYS-II E500 CW-EPR spectrometer (Bruker BioSpin) and processed by the Xepr software package. ESEEM measurements were conducted on an EPR-100 spectrometer (CIQTEK, China) with a low-temperature accessory. The circular dichlorism spectra were recored with a CD spectropolarimeter (J-815, JASCO) and processed with SpectraManager 2 software. Powder XRD patterns were collected on a Smartlab-9KW diffractometer equipped with a copper filter (Rigaku). SAED were carried out on a JEM-F200 system with cooling stage and energy-dispersive X-ray spectrometer (JEOL). XAFS were collected at 1W1B station in Beijing Synchrotron Radiation Facility (BSRF) with fluorescence mode. 1H NMR were recorded on AVANCE III HD 400 MHz (Bruker).
Data analysis	Statistical significance was determined with GraphPad Prism 8 software. The acquired EXAFS data were processed using the ATHENA module implemented in the IFEFFIT software packages. All the simulated EPR spectra were generated using EasySpin 5.2.35.

For manuscripts utilizing custom algorithms or software that are central to the research but not yet described in published literature, software must be made available to editors and reviewers. We strongly encourage code deposition in a community repository (e.g. GitHub). See the Nature Portfolio guidelines for submitting code & software for further information.

Data

Policy information about availability of data

All manuscripts must include a data availability statement. This statement should provide the following information, where applicable:

- Accession codes, unique identifiers, or web links for publicly available datasets
- A description of any restrictions on data availability
- For clinical datasets or third party data, please ensure that the statement adheres to our policy

All data are available within the main text or the Supplementary information. PDB: 1GYC, https://www.rcsb.org/structure/1GYC PDB: 1AOZ, https://www.rcsb.org/structure/1AOZ. All other relevant data are available from the corresponding authors upon request.

Research involving human participants, their data, or biological material

Policy information about studies with human participants or human data. See also policy information about sex, gender (identity/presentation), and sexual orientation and race, ethnicity and racism.

Reporting on sex and gender	N/A
Reporting on race, ethnicity, or other socially relevant groupings	N/A
Population characteristics	N/A
Recruitment	N/A
Ethics oversight	N/A

Note that full information on the approval of the study protocol must also be provided in the manuscript.

Field-specific reporting

Please select the one below that is the best fit for your research. If you are not sure, read the appropriate sections before making your selection.

☒ Life sciences ☐ Behavioural & social sciences ☐ Ecological, evolutionary & environmental sciences

For a reference copy of the document with all sections, see nature.com/documents/nr-reporting-summary-flat.pdf

Life sciences study design

All studies must disclose on these points even when the disclosure is negative.

Sample size	All experiments were performed in at least triplicate from independently assembled samples unless otherwise indicated in the text.
Data exclusions	No data excluded.
Replication	All attempts at replication were successful.
Randomization	Not applicable
Blinding	Not applicable

Reporting for specific materials, systems and methods

We require information from authors about some types of materials, experimental systems and methods used in many studies. Here, indicate whether each material, system or method listed is relevant to your study. If you are not sure if a list item applies to your research, read the appropriate section before selecting a response.

Materials & experimental systems

n/a	Involved in the study
☒	☐ Antibodies
☒	☐ Eukaryotic cell lines
☒	☐ Palaeontology and archaeology
☒	☐ Animals and other organisms
☒	☐ Clinical data
☒	☐ Dual use research of concern
☒	☐ Plants

Methods

n/a	Involved in the study
☒	☐ ChIP-seq
☒	☐ Flow cytometry
☒	☐ MRI-based neuroimaging
