# Supplementary material for: A supramolecular metalloenzyme possessing robust oxidase-mimetic catalytic function
Source: Nat Commun. 2023 Jul 7;14:4040. doi: 10.1038/s41467-023-39779-6 (PMC10328989; doi:10.1038/s41467-023-39779-6)
Supplement: Supplementary file 4 — Description of Additional Supplementary files [file 41467_2023_39779_MOESM4_ESM.pdf]

Description of Additional Supplementary Information

File name: Supplementary Data 1
Description: Crystallographic Data for simulated Fmoc-K crystal

File name: Supplementary Data 2
Description: Crystallographic Data for simulated Fmoc-H crystal
